# Variation in the mineral element concentration of *Moringa oleifera* Lam. and *M*. *stenopetala* (Bak. f.) Cuf.: Role in human nutrition

**DOI:** 10.1371/journal.pone.0175503

**Published:** 2017-04-07

**Authors:** Diriba B Kumssa, Edward JM Joy, Scott D Young, David W Odee, E Louise Ander, Martin R Broadley

**Affiliations:** 1 School of Biosciences, University of Nottingham, Sutton Bonington, Loughborough, United Kingdom; 2 Centre for Environmental Geochemistry, British Geological Survey, Keyworth, Nottingham, United Kingdom; 3 Crops For the Future, The University of Nottingham Malaysia Campus, Semenyih, Selangor, Malaysia; 4 Faculty of Epidemiology and Population Health, London School of Hygiene & Tropical Medicine, London, United Kingdom; 5 Kenya Forestry Research Institute, Nairobi, Kenya; 6 Centre for Ecology and Hydrology, Bush Estate, Penicuik, Midlothian, United Kingdom; Sun Yat-Sen University, CHINA

## Abstract

**Background:**

*Moringa oleifera* (MO) and *M*. *stenopetala* (MS) (family Moringaceae; order Brassicales) are multipurpose tree/shrub species. They thrive under marginal environmental conditions and produce nutritious edible parts. The aim of this study was to determine the mineral composition of different parts of MO and MS growing in their natural environments and their potential role in alleviating human mineral micronutrient deficiencies (MND) in sub-Saharan Africa.

**Methods:**

Edible parts of MO (n = 146) and MS (n = 50), co-occurring cereals/vegetables and soils (n = 95) underneath their canopy were sampled from localities in southern Ethiopia and Kenya. The concentrations of seven mineral elements, namely, calcium (Ca), copper (Cu), iodine (I), iron (Fe), magnesium (Mg), selenium (Se), and zinc (Zn) in edible parts and soils were determined using inductively coupled plasma-mass spectrometry.

**Results:**

In Ethiopian crops, MS leaves contained the highest median concentrations of all elements except Cu and Zn, which were greater in Enset (a.k.a., *false banana*). In Kenya, Mo flowers and MS leaves had the highest median Se concentration of 1.56 mg kg^-1^ and 3.96 mg kg^-1^, respectively. The median concentration of Se in MS leaves was 7-fold, 10-fold, 23-fold, 117-fold and 147-fold more than that in brassica leaves, amaranth leaves, baobab fruits, sorghum grain and maize grain, respectively. The median Se concentration was 78-fold and 98-fold greater in MO seeds than in sorghum and maize grain, respectively. There was a strong relationship between soil total Se and potassium dihydrogen phosphate (KH_2_PO_4_)-extractable Se, and Se concentration in the leaves of MO and MS.

**Conclusion:**

This study confirms previous studies that *Moringa* is a good source of several of the measured mineral nutrients, and it includes the first wide assessment of Se and I concentrations in edible parts of MO and MS grown in various localities. Increasing the consumption of MO and MS, especially the leaves as a fresh vegetable or in powdered form, could reduce the prevalence of MNDs, most notably Se deficiency.

## Introduction

Human micronutrient deficiencies (MNDs) are widespread in sub-Saharan Africa [1–3]. There is increasing interest in the potential role of underutilised crops to address MNDs and *Moringa* is one example [4]. *Moringa* is the sole genus of the flowering plant family Moringaceae, order Brassicales; [5]. It comprises 13 species of trees and shrubs (Table 1), namely, *M*. *arborea*, *M*. *borziana*, *M*. *concanensis*, *M*. *drouhardii*, *M*. *hildebrandtii*, *M*. *longituba*, *M*. *oleifera*, *M*. *ovalifolia*, *M*. *peregrine*, *M*. *pygmaea*, *M*. *rivae*, *M*. *ruspoliana*, and *M*. *stenopetala* [6]. Nine of the 13 species in the genus *Moringa* are native to lowlands of eastern Africa (i.e., south-eastern Ethiopia, Kenya and Somalia), of which, eight are considered endemic [7, 8]. The Horn of Africa is considered to be the centre of diversity of *Moringa* genus, but *Moringa oleifera* (MO) is the only species thought to originate outside Africa [8, 9]. *Moringa oleifera* and *M*. *stenopetala* (MS) are the two cultivated and most studied species [4, 10–19].

**Table 1:** Species in the Moringaceae family order Brassicales, current and synonymous binomial names, and species distribution [7].

Accepted binomial name	Synonym	Distribution
*Moringa arborea*		NE-Kenya
*Moringa borziana*	*Hyperanthera borziana*	S-Somalia, E-Kenya
*Moringa concanensis*	*Moringa concanensis*	SE-Pakistan (Baluchistan, Sind), India (widespread), W-Bangladesh
*Moringa drouhardii*		S-Madagascar
*Moringa hildebrandtii*		Madagascar (extinct in the wild, but frequently planted)
*Moringa longituba*	*Hyperanthera longituba*	NE-Kenya, SE-Ethiopia, Somalia
*Moringa oleifera*	*Anoma moringa*	Indigenous to N-India, Nepal, E-Pakistan; and Introduced in Costa Rica, Australia (Queensland), trop. Africa, Java, Malesia, Jamaica, Lesser Antilles (St. Martin, St. Barts, Antigua, Saba, St. Eustatius, St. Kitts, Montserrat, Guadeloupe, Martinique, St. Lucia, St. Vincent, Grenadines, Grenada, Barbados), Panama, Belize, Aruba, Bonaire, Curacao, Haiti, Dominican Republic, Bahamas, Cuba, Nicaragua, Mexico, Venezuela, Brazil (c), Seychelles, Somalia, New Caledonia, Fiji, Christmas Isl. (Austr.), Palau Isl. (Koror, Namoluk, Pohnpei), Society Isl. (Tahiti, Raiatea), Southern Marianas (Saipan, Rota, Guam), Niue, Mauritius, Réunion, Rodrigues, Madagascar, Yemen, Oman, Cape Verde Isl. (Santo Antao Isl., Sal Isl., Ilha de Maio, Ilha de Sao Tiago, Fogo Isl.), Ryukyu Isl., Andamans, Nicobars, Myanmar [Burma], Vietnam, Bhutan, Sikkim, Sri Lanka, Laos, Philippines, USA (Florida), U.S. Virgin Isl.
	*Guilandia moringa*	
	*Hyperanthera arborea*	
	*Hyperanthera decandra*	
	*Hyperanthera moringa*	
	*Hyperanthera pterygosperma*	
	*Moringa domestica*	
	*Moringa edulis*	
	*Moringa erecta*	
	*Moringa moringa*	
	*Moringa nux-eben*	
	*Moringa octogona*	
	*Moringa parvifolia*	
	*Moringa polygona*	
	*Moringa pterygosperma*	
	*Moringa robusta*	
	*Moringa sylvestris*	
	*Moringa zeylanica*	
*Moringa ovalifolia*		South Africa (Transvaal), Namibia, SW-Angola
*Moringa peregrina*	*Gymnocladus arabica*	Egypt (Eastern Desert, SE-Egypt), Israel (E-Israel: Rift Valley, SC-Israel: Judean Desert, S-Negev Desert), Jordania (S-Jordania), Oman (Dhofar, Mascat & Oman), Saudi Arabia (C-Saudi Arabia, N-Saudi Arabia, NW-Saudi Arabia: Hejaz, SW-Saudi Arabia: Asir), Sinai peninsula (Southern Sinai), Yemen (Aden Desert, coastal Hadhramaut, NE-Yemen: Inner Hadhramaut, SW-Yemen, Tihama), United Arab Emirates, N-Sudan, N-Ethiopia, Eritrea, Somalia, India
	*Hyperanthera aptera*	
	*Hyperanthera arborea*	
	*Hyperanthera monodynama*	
	*Hyperanthera peregrina*	
	*Hyperanthera semidecandra*	
	*Moringa aptera*	
	*Moringa arabica*	
*Moringa pygmaea*		NE-Somalia
*Moringa rivae* subsp. *longisiliqua*		S-Ethiopia
*Moringa rivae* subsp. *rivae*	*Hyperanthera rivae*	S-Somalia, S-Ethiopia, Kenya
*Moringa ruspoliana*	*Hyperanthera ruspoliana*	Somalia, SE-Ethiopia, NE-Kenya
*Moringa stenopetala*	*Donaldsonia stenopetala*	
	*Moringa streptocarpa*	SW-Ethiopia, N-Kenya

**Fig 1:**
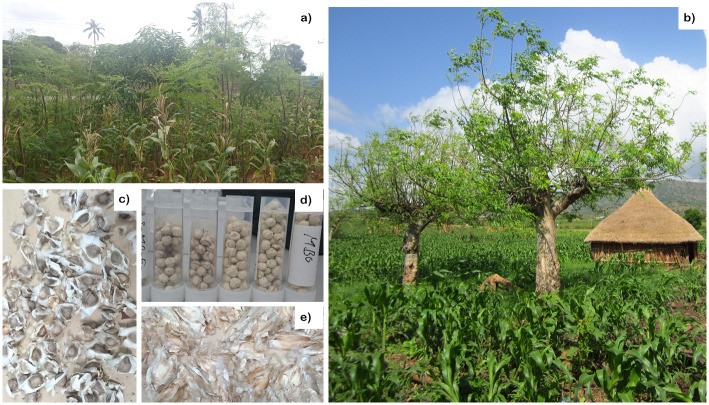
*M*. *oleifera* tree intercropped with maize at Malindi, Kenya (a); *M*. *stenopetala* trees intercropped with maize, southern Ethiopia (b); Husked *M*.*oleifera* seeds (c); husked *M*. *stenopetala* seeds (d); and kernel of *M*. *oleifera* (e).

*Moringa oleifera* (Fig 1) is indigenous to the Himalayan foothills of south India [20]. It has been naturalized to tropical and sub-tropical Asia; Middle East; Africa; and America [8, 21–24]. This pantropical species is known by various names. In English, it is known as *drumstick* tree due to the shape of its pods, *never die tree* due to its ability to thrive under marginal environmental conditions, and *mother’s best friend* due to its nutritious edible parts that help revive malnourished children [25]. It is known as *Mlonge/ Mzunze/ Mjungu moto/ Mboga chungu/ Shingo* in Kenya [8]. *Moringa stenopetala* (Fig 1) is native to southern Ethiopia and northern Kenya [26, 27]. In southern Ethiopia, it is locally known as *Haleko* in Walayita and Konso languages.

*Moringa oleifera* and MS are fast growing multipurpose woody plants which grow in diverse ecosystems [8, 21, 22, 28, 29], from very dry marginal lowland tropical climates to moist high altitude regions. They shed their leaves during long dry seasons. Their tuberous roots enable them to store water and withstand very long dry seasons. The MO tree can grow up to 5–15 m in height, with a diameter at breast height up to 25 cm [8, 21, 22]. A mature MS tree is usually larger in overall size and more drought tolerant than MO, with larger leaves, seeds and trunk. However, MS is slower-growing compared to MO. In experiments conducted in the Sudan, MS flowered after 2.5 years as compared to 11 months for MO [30].

### Nutritional uses

Dietary diversification using underutilized crops/trees, such as *Moringa* spp. is one of the many alternative strategies to fight MNDs [2, 3, 31–33]. However, data on nutritional contents of such under-utilised vegetables and understanding of environmental/genetic variation in trace elements concentration are limited. Ethnobotanical and biochemical studies carried out in various countries where *Moringa* grow show that these species are multipurpose. They are used for food, medicine, fodder, fencing, firewood, gum and as a coagulant to treat dirty water [21, 23, 34–38]. The foliage, immature pods, seeds, and roots are used both as food and medicine. Young shoots are also cooked and eaten [23, 25]. Leaves are either cooked or consumed raw as vegetables. *Moringa* leaves are used in a similar way as a cabbage and spinach thereby nicknamed ‘cabbage tree’ [39]. As a food or forage source, *Moringa* spp. can supply a wide range of essential macro and micro nutrients [4, 25, 40, 41]. The mean concentration of Ca, Cu, Fe, Mg, and Zn in MO leaves collected from a garden in Jalisco State of Mexico were 16100, 9.6, 97.9, 2830 and 29.1 mg kg^-1^ dry weight (dw), respectively. Similarly, the concentrations of these elements in MS leaves were 12700, 9.1, 69.9, 3690 and 33.7 mg kg^-1^ dw, respectively [4]. A mean Se concentration of 0.877 mg kg^-1^ dw was reported in MO leaves grown at six locations ranging from 0.455 mg kg^-1^ dw in Rwanda to 2.00 mg kg^-1^ dw in the Solomon Islands [42]. However, systematic analysis of Se has not been conducted at multiple sites within a country and concentrations of other elements such as iodine have not been reported.

### Impact of environment on mineral element concentration in *Moringa* edible parts

The mineral element concentrations in different edible parts of *Moringa* spp. are affected by the environment in which they grow. For example, the effect of elevation and season on mineral micronutrient concentration of leaves and immature pods of MO and MS was studied in Ethiopia [40]. Concentrations of Ca, Fe, and Zn in *Moringa* leaves grown in mid-altitude areas during the rainy season were 24800, 578 and 24.3 mg kg^-1^ dw for MO and 14900, 700 and 24.7 mg kg^-1^ dw for MS, respectively. In low altitude areas, the Ca, Fe, and Zn concentration in MO leaves during rainy season were 25700, 564 and 26 mg kg^-1^ dw, while in MS leaves, the concentrations were 24000, 581 and 28.1 mg kg^-1^ dw, respectively [40]. Other studies have compared MO samples collected from various sites without specifying environmental variables. For example, in their study on the mineral concentration of MO edible parts in two regions of Nigeria, it was reported that Ca, Mg, Fe and Cu concentration in the leaves, pods and seeds were higher in tissues collected from Sheda region than Kuje, Abuja [43]. Similarly, a study conducted in the Punjab province of Pakistan indicated that the Ca, Mg, and Zn concentration in the leaves and pods of MO varied significantly by region [44]. For example, the Ca concentration in MO leaves in Bahawalnagar and Sadiqabad were 22900 and 19000 mg kg^-1^ dw respectively. A study conducted by Olson *et al*. [4] indicated variation in leaf elemental concentration between 12 *Moringa* species grown in a common garden experiment.

### Study aims

To our knowledge, no studies have explored the association between plant tissue element concentration of *Moringa* spp., and the site-specific physico-chemical properties of the soil. Previous studies assessing the variation in the elemental concentration in edible parts of *Moringa* spp. in various agro-ecological zones have typically been based on generic classifications, e.g., elevation [40]. Furthermore, there is some evidence that *Moringa* accumulates Se [42] but this has not been widely confirmed in leaves or for other plant parts. Iodine concentrations have not previously been reported in *Moringa* leaves. The objectives of this study were to:

- determine the multi-elemental concentration in the flowers, immature pods, leaves, and seed kernels of MO and MS grown in different agro-ecological zones in Ethiopia and Kenya;
- explore the association between MO and MS edible parts mineral element concentration and soil physico-chemical properties;
- assess the potential of consumption of MO and MS leaves in alleviating dietary micronutrient deficiencies in sub-Saharan Africa; and
- compare the mineral element concentrations in MS and MO edible parts with locally grown cereal and vegetable crops.

## Materials and methods

**Fig 2:**
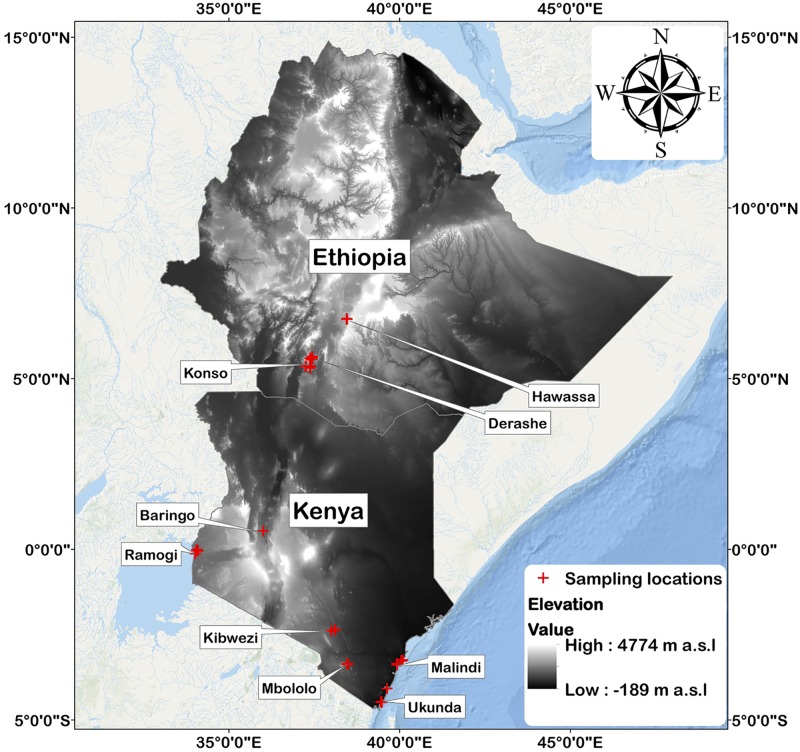
Sample collection localities in Ethiopia and Kenya, and grey scale altitudinal range for Ethiopia and Kenya. Country boundaries shape files were downloaded from the Global Administrative Areas database (http://gadm.org/, Version 2, January 2012), and digital elevation data were downloaded from http://www.diva-gis.org/Data. Map was created using ESRI ArcMap^™^ 10.3.1 software.

This study was conducted in southern Ethiopia and Kenya. Sample collections from localities in southern Ethiopia were carried out in December 2014 and April 2015, and in July 2015 from localities in Kenya (Fig 2). Edible parts of MO and MS were sampled from plants that were cultivated by *Moringa* growing households after receiving their consent. The study was carried out on private/communal land with the owners’ permission, and it did not involve endangered or protected species. The study received ethical approval from the University of Nottingham, School of Biosciences Research Ethics Committee (SB REC), approval number: SBREC140117A.

### Study sites

Edible parts of MO, MS, other food crops, and soil samples, were collected from localities in southern Ethiopia and Kenya (Fig 2 and S1 Table). Site selection was conducted by the guidance of local agricultural development agents who knew about the localities and households that cultivate *Moringa* trees. In addition, different sites with varying soil types were surveyed. The altitude of the locations ranged from 13 m a.s.l. in Malindi, Kenya to 1700 m a.s.l. in Hawassa, Ethiopia.

### Plant multi-elemental analyses

#### Sample collection and preparation

A total of 196 *Moringa* plant edible parts with ≥ 3 samples per site for each tissue (i.e., flowers, leaves, immature pods, seeds and roots) were collected from southern Ethiopia and Kenya (). The edible parts collected from MS were limited to leaves due to unavailability of other tissues during the sampling campaign. Cereal grains and vegetable crops were also collected from some of the farmers’ fields that grew those crops in combination with *Moringa* trees in Kenya. Similarly, various cereal and pulse grains were acquired from households that took part in the survey from Ethiopia. Fresh *Moringa* leaves were washed in the field by using either tap or bottled water. Fresh edible plant samples collected from Ethiopia were air dried and those from Kenya were oven-dried at 40–50°C at Kenyan Forestry Research Institute (KEFRI) headquarters in Nairobi and transferred to the University of Nottingham, UK, for further processing and chemical analyses. The dried edible parts, and grains were milled using an ultra-centrifugal mill to pass through a 1 mm screen (ZM 200, Retsch GmbH, Haan, Germany).

#### Nitric acid digestion of plant samples

Subsamples (c. 0.2 g) of the milled plant samples were weighed in triplicate for nitric acid (HNO_3_) digestion and subsequent multi-elemental analysis. Samples were mixed with 6 mL of HNO_3_ (PrimarPlus—Trace Analysis Grade (TAG), Fisher Scientific, Loughborough, UK) in microwave digestion tubes and digested at 140°C for 20 min (Multiwave PRO, Anton Paar, St. Albans, UK). After cooling, the samples were diluted with 14 mL of Milli-Q water (MQW) (18.2 MΩ cm; Merck Millipore Milli-Q, Darmstadt, Germany) prior to multi-elemental analysis by inductively coupled plasma-mass spectrometry (ICP-MS; iCAP-Q, Thermo-Scientific, Loughborough, UK) following a further 1-in-10 dilution with MQW.

#### TMAH-extractable plant Iodine (I)

Iodine was extracted from 0.2 g milled plant material in 5 mL of 5% tetramethylammonium hydroxide (TMAH) solution (25% w/w aq. Soln., Electronic Grade, 99.9999% [metal basis] Alfa Aesar, Ward Hill, MA, USA), with microwave heating at 110°C, for 20 min. The digested samples were diluted to 25 mL with MQW and centrifuged at 3000 rpm for 30 min (Heraeus Megafuge 40 Centrifuge, Thermo Scientific, Osterode am Harz, Germany) in a single use 50 mL centrifuge tubes (SUCT) (Fisherbrand, Fisher Scientific, Pittsburgh, USA). Supernatant solutions were then filtered using a 0.22 μm syringe filter (SF) (Millex PES, Merck Millipore Darmstadt, Germany) and transferred to sample tubes for ICP-MS analysis. Due to the high viscosity of the digestates from starchy seeds and grains which blocked the ICP-MS auto sampler needle, plant iodine analyses were conducted on *Moringa* leaves only.

### Soil multi-elemental analysis

#### Sample collection and preparation

Thirty-three and 62 soil samples were collected from southern Ethiopia and Kenya, respectively (S2 Table). Each sample comprised soil pooled from five locations underneath the canopy of a *Moringa* tree spp. Bulked samples were air dried and sieved to pass through < 2 mm screen. A subsample of 30 g was taken to the University of Nottingham. From each sample, a 10 g subsample was Agate ball-milled (PM 400, Retsch, Haan, Germany) for multi-elemental analyses.

#### Multi-acid digestion of soils

Triplicate finely ground soil samples (c. 0.2 g) were digested for two days with 2.5 mL hydrofluoric acid (HF) (40% AR), 2 mL HNO_3_ (70% TAG), 1 mL perchloric acid (HClO_4_) (70% AR) and 2.5 mL MQW in PFA tubes on a Teflon-coated graphite block digester (Model A3, Analysco Ltd, Chipping Norton, UK). On the third day, the hot plate heating was turned off and 2.5 mL concentrated HNO_3_ (70% TAG) and MQW were added and heated for 1 h at 50°C. After cooling, the digestates were made up to 50 mL in plastic volumetric flasks. Multi-elemental analyses were undertaken by ICP-MS following a further 1-in-10 dilution.

#### Phosphate-extractable soil Se (Se-P)

Duplicate soil samples (< 2 mm; c. 2 g) were shaken in SUCT for 1 h on a rotary shaker with 20 mL of 0.016 M potassium dihydrogen phosphate (KH_2_PO_4_) [45]. The soil suspensions were centrifuged at 2200 rpm for 20 min and 10 mL of supernatant solution was filtered through a SF prior to Se-P analyses by ICP-MS.

#### TMAH-extractable soil iodine

Finely milled duplicate 2 g soil samples were mixed with 10 mL of 10% TMAH in a SUCT. The soil suspensions were heated in an oven at 70°C (Memmert GmbH + Co, D 06061, Model 500, Schwabach, Germany) for 3 h and then centrifuged at c. 3000 rpm for 20 min. The supernatant solution was diluted 1-in-10 with MQW prior to analysis for iodine by ICP-MS.

#### Soil pH

The < 2 mm sieved soil was mixed with deionized water at a ratio of 5 g:12.5 mL in SUCT and shaken for 30 min on a rotary shaker. The pH of the mixture was measured using combined pH meter and electrode (HI-209 pH/mV pH Meter, Hanna Instruments Ltd., Leighton Buzzard, UK). Prior to taking the pH readings, the electrode was calibrated using buffers at pH of 4.01 and 7.00. After each reading, the glass electrode was rinsed by deionized water before measuring the pH of the next sample.

### Analytical quality control

For analytical quality control, blanks, duplicates, internal standards and certified reference materials were analysed in all instances of plant and soil analyses. The certified reference materials were tomato leaves (1573A), wheat flour (1567B), and Montana soil II (2711A) from the National Institute of Standards and Technology, Gaithersburg, MD, USA (S3 and S4 Tables). Raw data of the plant and soil sample analytical results is presented as supplementary tables (S34–S39 Tables).

### Data analyses

Research data compilation and management were carried out using Microsoft Excel and Access 2016 (Microsoft, Redmond, USA). Statistical analyses of the elemental concentration in edible plant parts and the soils were conducted using IBM^®^ SPSS^®^ Statistics version 22 (IBM Corp., New York, USA). The Shapiro-Wilk test for normality of the distribution of the data and Levene’s test for homogeneity of variance were run to select between parametric and non-parametric analyses of variance (ANOVA) (S5–S16 Tables). In addition, visual assessments of the data distributions were made. Due to the small sample size at each locality, most of the plant and soil elemental concentration data did not meet the assumptions of parametric ANOVA; logarithmic transformation did not improve the non-normal distribution and heteroscedasticity of the elemental concentration data. Hence, Welch’s robust test for equality of means was applied to test the variation in elemental concentration by locality. Spearman’s rank correlation analysis was conducted using GenStat^®^ version 17 (VSN International, Hemel Hempstead, UK) to assess the association between soil physico-chemical properties and *Moringa* leaves elemental concentration, and relationships between elemental concentrations in edible parts of various vegetables. Box plots of plant and soil elemental concentration and pH were drawn using Tableau^®^ Desktop Professional Edition version 10.0.0 (Tableau Software Inc., Seattle, Washington, USA). Outliers were not included in the box plots. Plant edible parts with sample size < 3 per locality were excluded from statistical analyses. For instance, there was only one MO and MS sample at Baringo and Ramogi, respectively. These were not included in the data analyses.

## Results

Plant edible parts and soil elemental concentration analytical results for calcium (Ca), copper (Cu), iodine (I), iron (Fe), magnesium (Mg), selenium (Se), and zinc (Zn); and soil pH are reported. The association between plant edible parts elemental concentration and soil properties, and variation in elemental concentration by location are also reported. Furthermore, comparisons are made among *Moringa* spp. edible parts, maize and sorghum grains, beans, amaranth leaves, baobab fruit, brassica leaves, and enset (*Ensete ventricosum* a.k.a., *false banana*), mineral element concentrations.

### *Moringa* elemental concentration

The concentrations of mineral elements in *Moringa* leaves, immature pods, seeds and flowers and variations by localities are presented below, and summarised in Figs 3—7, S17—S21 Tables and Table 2.

#### *Moringa oleifera* leaf elemental concentration

**Fig 3:**
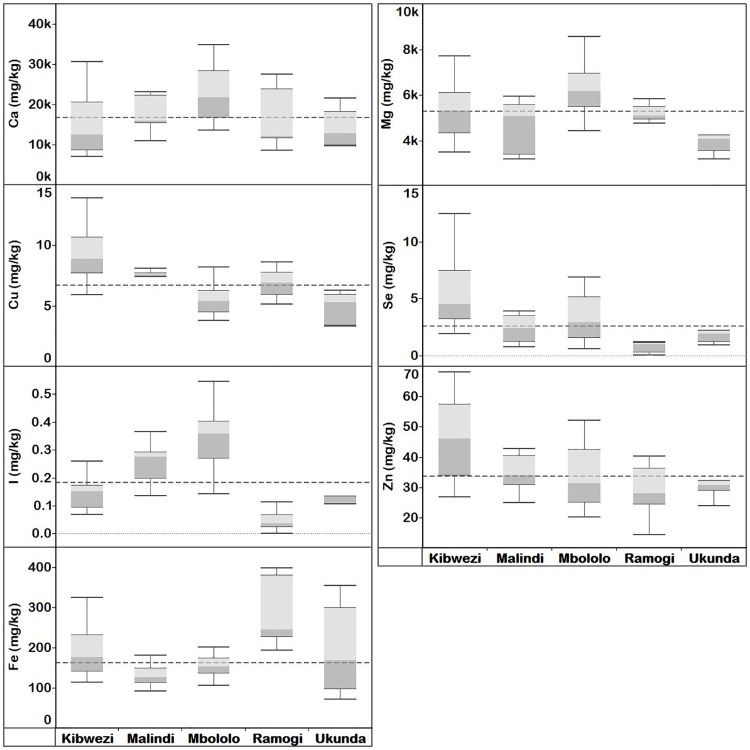
Quartiles of elemental concentration (mg kg^-1^ dw) in *M*. *oleifera* leaves collected from Kenya (Kibwezi, Malindi, Mbololo, Ramogi and Ukunda) localities. Median elemental concentration for each locality is where the light and dark grey shading boxes meet. The horizontal broken lines depict the overall median concentration for each element across localities.

The overall mean concentrations of Ca, Cu, I, Fe, Mg, Se and Zn in MO leaves were 18300, 6.92, 0.218, 202, 5390, 4.25 and 35.6 mg kg^-1^ dw, respectively (S17 Table). Mineral element concentration of the MO leaves varied significantly (*p* < 0.05) between localities, except for Ca (Fig 3 and S27 Table). There was no systematic variation in the relative concentration at a given location for these elements, although Kibwezi had the highest values of the trace elements (Cu, Se, Zn).

#### *Moringa stenopetala* leaf elemental concentration

**Fig 4:**
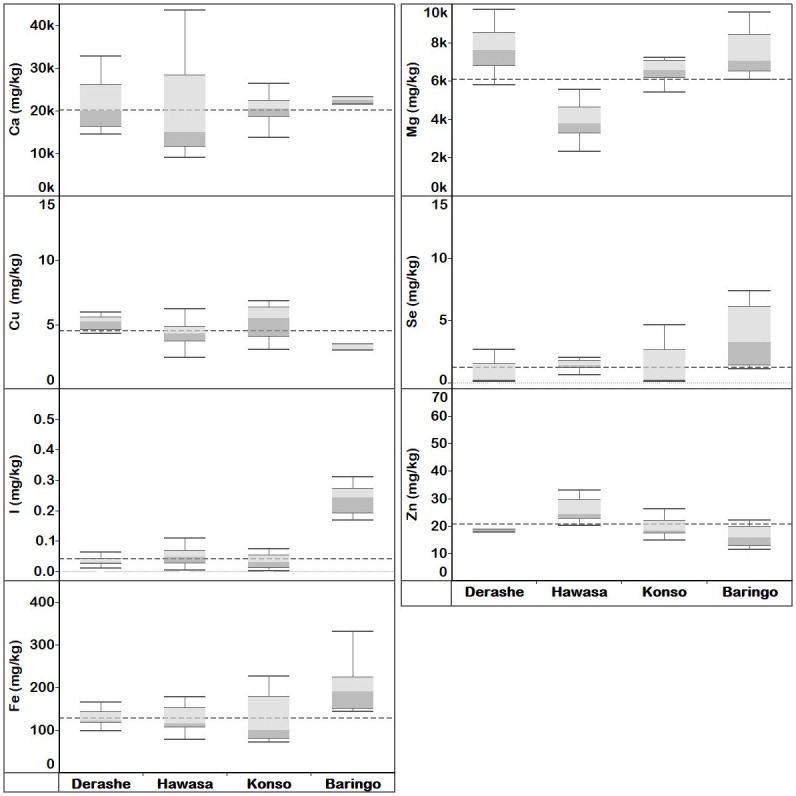
Quartiles of elemental concentration (mg kg^-1^ dw) in *M*. *stenopetala* leaves collected from Ethiopia localities (Derashe, Hawassa and Konso), and Kenya locality (Baringo). Median elemental concentration for each locality is where the light and dark grey shading boxes coincide. The horizontal broken lines depict the overall median concentration for each element across localities.

The overall mean concentrations of Ca, Cu, I, Fe, Mg, Se and Zn in MS leaves were 21100, 4.53, 0.07, 162, 6440, 1.66 and 22.2 mg kg^-1^ dw, respectively (S18 Table). Mean Cu, I, Mg and Zn differed significantly between localities (*p* < 0.05), while Ca, Fe, and Se concentrations of MS leaves did not differ significantly between localities (S28 Table). *Moringa stenopetala* leaves collected from Kenya (n = 5) had higher median concentrations of mineral elements than those from Ethiopia (n = 36) except Cu and Zn (Fig 4 and S18 Table). MS leaves from Hawassa, southern Ethiopia had significantly (*p* < 0.05) higher concentration of Zn and lower Mg than those from Baringo Island, Kenya. On the contrary, MS leaves collected from Baringo island contained significantly (*p* < 0.05) higher concentrations of Se and I than all samples from localities in Ethiopia. The concentration of Cu in MS leaves collected from Baringo island were significantly (*p* < 0.05) lower than samples from Derashe, southern Ethiopia.

#### *Moringa oleifera* immature pods elemental concentration

**Fig 5:**
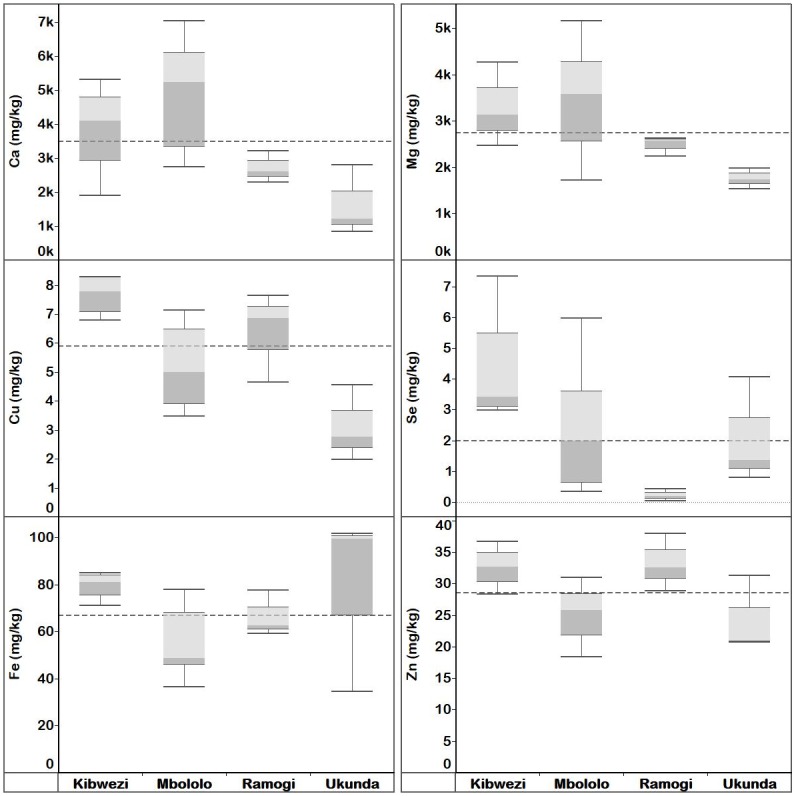
Quartiles of elemental concentration (mg kg^-1^ dw) in *M*. *oleifera* immature pods collected from Kenya localities. Median elemental concentration for each locality is where the light and dark grey shading boxes coincide. The horizontal broken lines depict the overall median concentration for each element across localities.

The overall mean concentrations of Ca, Cu, Fe, Mg, Se and Zn in MO immature pods were 3600, 5.42, 65.4, 2860, 2.36 and 27.6 mg kg^-1^ dw, respectively (S19 Table). The distribution of MO immature pods elemental concentration in comparison with the overall median value varied between the elements and locations (Fig 5). For example, the median elemental concentration in the immature pods collected from Kibwezi were generally higher than the overall median concentration in Kenya. The median Ca and Mg concentration in immature pods collected from Ramogi and Ukunda were below the overall median concentration in Kenya. There were significant differences (*p* < 0.05) in the Cu, Fe and Mg but not (*p* ≥ 0.05) Ca, Se and Zn mean concentrations of MO immature pods collected from different localities (S30 Table).

#### *Moringa oleifera* seeds elemental concentration

**Fig 6:**
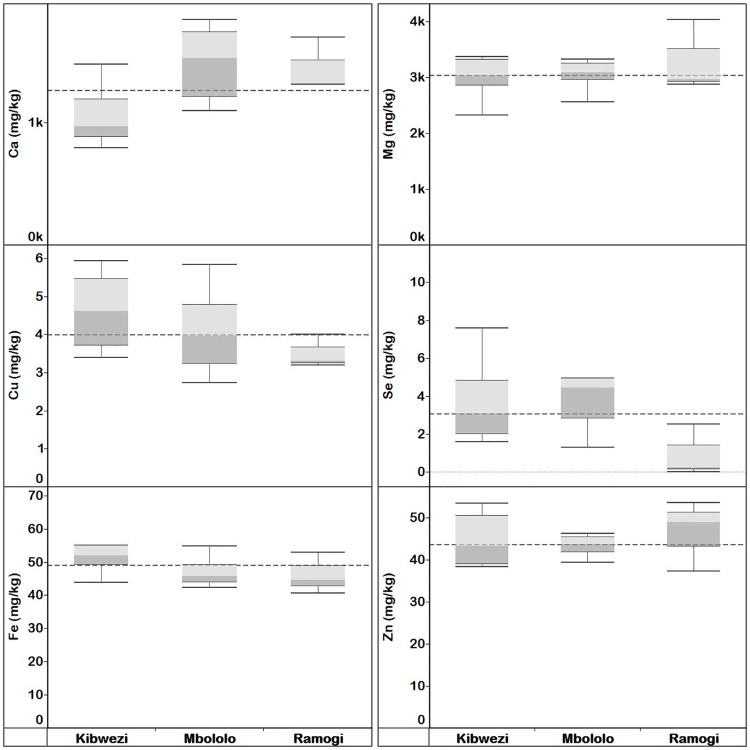
Quartiles of elemental concentrations in *M*. *oleifera* seeds (mg kg^-1^ dw) collected from Kenya localities. Median elemental concentration for each locality is where the light and dark grey shading boxes coincide. The horizontal broken lines depict the overall median concentration for each element across localities.

The overall mean concentrations of Ca, Cu, Fe, Mg, Se and Zn in MO seeds were 1310, 4.18, 49.2, 3080, 3.59 and 44.8 mg kg^-1^ dw, respectively (S20 Table). Overall median elemental concentration in MO seeds varied between the elements and locations (Fig 6). There was no significant difference (*p* ≥ 0.05) in the mean elemental concentration of MO seeds collected from different localities (S31 Table).

#### *Moringa oleifera* flowers elemental concentration

**Fig 7:**
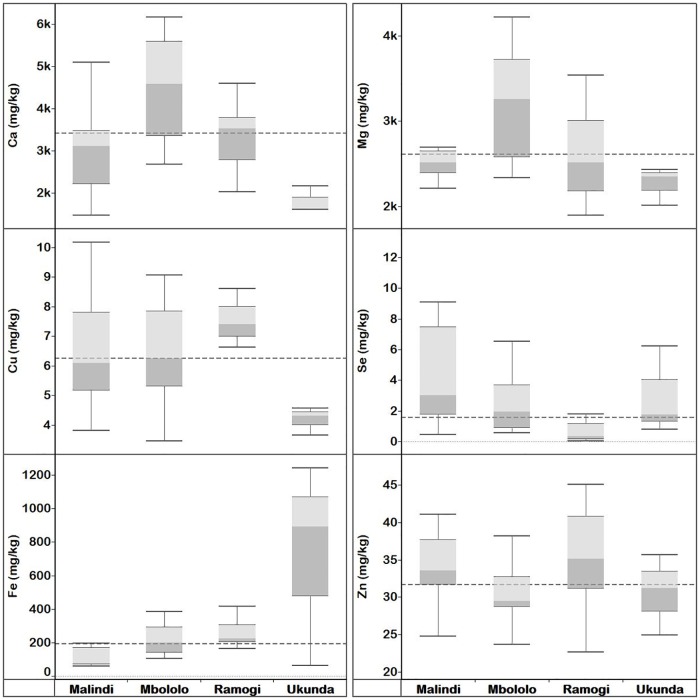
Quartiles of elemental concentrations (mg kg^-1^ dw) in *M*. *oleifera* flowers collected from Kenya localities. Median elemental concentration for each locality is where the light and dark grey shading boxes coincide. The horizontal broken lines depict the overall median concentration for each element across localities.

The overall mean concentrations of Ca, Cu, Fe, Mg, Se and Zn in MO flowers were 3650, 6.40, 253, 2830, 2.81 and 32.7 mg kg^-1^ dw, respectively (S21 Table). The distribution of MO flowers elemental concentration in comparison with the overall median value varied between elements and locations (Fig 7). There were significant differences (*p* < 0.05) in the mean Ca, Cu, Fe, Mg and Se, but not Zn concentrations of MO flowers collected from different localities (S32 Table).

### Comparison of elemental concentration between crops

For comparison, elemental concentration of *Moringa* spp. edible parts and other vegetables, fruits, and staple cereal crops are presented in Table 2. On a weight-for-weight basis, in Ethiopia, MS leaves contained the highest median concentrations of all elements except Cu and Zn. Median concentrations of Cu and Zn were highest in enset and beans, respectively (Table 2).

**Table 2:** Median elemental concentrations in cereals, vegetables, fruits and seeds grown in various parts of Ethiopia and Kenya, and the number of samples (n).

Crop	n	Median concentration (mg kg^-1^ dw)						
		Ca	Cu	I	Fe	Mg	Se	Zn
**Ethiopia**								
MS leaves	36	19400	4.71	0.093	117	6070	1.12	21.0
Maize grain	17	55.1	0.943		28.2	918	0.182	20.4
Enset	5	2,190	1.30		71.3	260	0.060	34.2
Sorghum grain	8	176	1.74		51.5	1350	0.097	16.1
Beans	4	1500	9.41		88.5	1760	0.150	24.9
**Kenya**								
Amaranth leaves	6	26700	6.88		339	12900	0.399	33.9
Baobab fruits	4	2660	7.63		9.40	1180	0.169	11.9
Brassica leaves	4	32000	9.25		104	7880	0.597	17.4
Maize grain	9	51.2	2.80		16.5	912	0.027	21.5
MO flowers	33	3420	6.25		4.73	2610	1.56	31.7
MO immature pods	25	3060	5.05		62.6	2610	1.99	27.8
MO leaves	56	16700	6.83	0.201	160	5400	2.73	33.3
MO seeds	32	1250	4.02		49.8	3100	2.64	46.0
MS leaves	5	22500	3.07	0.231	190	7210	3.96	15.7
Sorghum grain	6	103	6.24		33.7	1220	0.034	22.6

In Kenya, on weight-for-weight basis, *Moringa* edible parts had the highest median Se concentration ranging from 1.56 mg kg^-1^ in MO flowers to 3.96 mg kg^-1^ in MS leaves. The median concentration of Se in MS leaves was 7-fold, 10-fold, 23-fold, 117-fold and 147-fold more than that in brassica leaves, amaranth leaves, baobab fruits, sorghum grain and maize grain, respectively. The median Se concentration in MO seeds was 78-fold and 98-fold greater than sorghum and maize grain, respectively. Seeds of MO had the highest median Zn concentration while amaranth leaves contained comparable quantities of Zn with MO flowers and leaves. The median Zn concentration in MO seeds was 2-fold greater than in maize and sorghum grain. (Table 2).

### Soil pH and elemental concentrations

**Fig 8:**
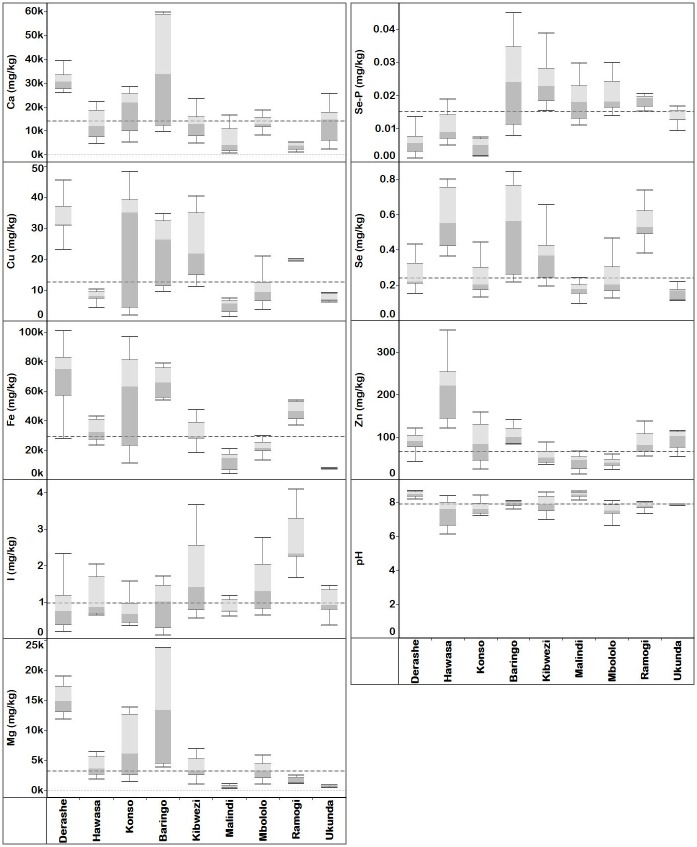
Quartiles of soil elemental concentration (mg kg^-1^ dw) and pH collected from southern Ethiopia (Derashe, Hawassa and Konso), and Kenya (Baringo, Kibwezi, Malindi, Mbololo, Ramogi and Ukunda) localities. Median elemental concentration for each locality is where the light and dark grey shading boxes coincide. The horizontal broken lines depict the overall median of each property across localities. Total soil elemental concentrations except for Se-P, which is KH_2_PO_4_-extractable fraction.

Ninety percent of the soil samples from Ethiopia and 97% of those from Kenya had pH >7 (S37 Table). The soil pH at the three localities in Ethiopia ranged from 6.12 in Hawassa to 8.67 in Derashe, with overall mean and median of 7.84 and 7.98, respectively. In Kenya, soil pH ranged from 6.63 in Mbololo to 8.65 in Malindi with overall mean and median of 7.88 and 7.85, respectively (Fig 8, Table 3 and S22 Table). Welch’s robust test of equality of means showed that the soil pH varied significantly between localities (*p* < 0.05) (S33 Table). Similarly, Welch’s robust tests of equality of mean soil elemental concentrations showed that there was significant difference (*p* < 0.05) between soils collected from various localities (S33 Table). Descriptive statistics of the soil physico-chemical properties across all localities in Ethiopia and Kenya are summarized in Table 3. Soil samples from Baringo, Kibwezi and Ramogi localities were the three with highest median phosphate-extractable Se concentration. Total Se concentration was highest in soils from Baringo, Hawassa and Ramogi. With respect to total soil iodine, Ramogi, Kibwezi and Mbololo soil samples had the highest concentrations. Total Zn concentration in soil samples from Hawassa were 2-fold, 4-fold and 3-fold more than the median Zn concentration from soils in Ethiopia, Kenya and overall median Zn concentrations.

**Table 3:** Descriptive statistics of soil pH, and elemental concentrations by locality.

Locality	Statistic	Concentration (mg kg^-1^ dw)								
		Ca	Cu	I	Fe	Mg	Se	Se-P	Zn	pH
**Baringo**	**n**	5	5	5	5	5	5	5	5	5
	**Mean**	38400	23.7	0.923	66200	15000	0.550	0.027	97.7	7.88
	**Median**	52000	31.1	1.06	67000	19500	0.664	0.035	92.9	7.96
**Derashe**	**n**	12	12	12	12	12	12	12	12	12
	**Mean**	28400	33.4	0.851	69200	13700	0.255	0.006	88.0	8.40
	**Median**	30600	31.2	0.748	74900	14800	0.222	0.006	90.7	8.48
**Hawassa**	**n**	9	9	9	9	9	9	9	9	9
	**Mean**	15800	13.0	1.13	35400	4800	0.599	0.011	260	7.43
	**Median**	14100	8.62	0.941	32900	3800	0.622	0.010	229	7.66
**Kibwezi**	**n**	14	14	14	14	14	14	14	14	14
	**Mean**	14200	24.0	1.72	33000	4020	0.362	0.024	54.0	7.88
	**Median**	12800	21.7	1.41	29300	3260	0.366	0.023	51.5	7.89
**Konso**	**n**	12	12	12	12	12	12	12	12	12
	**Mean**	18700	26.2	0.771	56000	7120	0.239	0.004	85.725	7.595
	**Median**	21800	35.0	0.676	63100	6100	0.204	0.005	83.1	7.59
**Malindi**	**n**	11	11	11	11	11	11	11	11	11
	**Mean**	7290	4.72	0.955	12200	566	0.179	0.019	39.9	8.40
	**Median**	4000	5.63	0.768	14500	591	0.177	0.018	45.4	8.44
**Mbololo**	**n**	16	16	16	16	16	16	16	16	16
	**Mean**	13400	10.3	1.43	22300	3210	0.241	0.020	39.8	7.53
	**Median**	12900	9.38	1.30	21300	3260	0.203	0.018	39.7	7.50
**Ramogi**	**n**	8	8	8	8	8	8	8	8	8
	**Mean**	3550	35.5	3.24	68900	2210	0.550	0.018	87.8	7.81
	**Median**	3670	21.3	2.81	53600	2020	0.529	0.019	81.6	7.81
**Ukunda**	**n**	7	7	7	7	7	7	7	7	7
	**Mean**	12900	7.55	1.00	7690	715.3	0.155	0.014	92.3	7.94
	**Median**	14600	7.07	0.923	8040	839	0.169	0.013	102	7.83
**Ethiopia**	**n**	33	33	33	33	33	33	33	33	33
	**Mean**	21400	25.2	0.898	55200	8870	0.343	0.007	134	7.84
	**Median**	22300	30.7	0.773	51200	6700	0.281	0.006	101	7.98
**Kenya**	**n**	61	61	61	61	61	61	61	61	61
	**Mean**	13200	16.5	1.56	31000	3470	0.313	0.020	60.2	7.88
	**Median**	11800	11.3	1.20	23500	2300	0.239	0.018	53.5	7.85
**Total**	**n**	94	94	94	94	94	94	94	94	94
	**Mean**	16100	19.6	1.33	39500	5360	0.324	0.016	86.1	7.87
	**Median**	13700	13.0	0.972	29700	3130	0.240	0.015	66.0	7.88

### Relationships between MO edible parts and other vegetables

The association between elemental concentrations of the edible parts of MO are presented in S40 Table. Calcium concentration in MO flowers showed highly significant (*p* < 0.01) positive correlation with the Ca, Cu, Mg, and Se concentration in MO immature pods. Contrary to this, the Cu concentration in MO flowers had a significant (*p* < 0.01) negative correlation with the Ca in the seeds and leaves. The Se in the MO flower was highly significantly (*p* < 0.01) correlated with the Se in leaves and immature pods. The association between the elemental concentrations in MO edible parts and other vegetables cultivated on the same location are presented in S41–S43 Tables. The Fe concentration in MO leaves showed significant (*p* < 0.05) positive correlation with the Fe, and negative correlation with the Se concentration in amaranth leaves (S41 Table). Calcium concentration in MO leaves showed significant (*p* < 0.01) negative correlation with the Ca, Cu, and Mg concentration in brassica leaves (S42 Table). Similarly, Ca concentration in amaranth leaves showed a significant (*p* < 0.01) negative correlation with the Ca, Cu, and Mg concentrations in brassica leaves (S43 Table).

### Relationships between *Moringa* leaves elemental concentration and soil properties

The concentration of Cu in MO leaves was significantly (*p* < 0.05) positively associated with total soil Cu and Fe concentrations. Similarly, MO leaf Fe concentration also showed a statistically significant (*p* < 0.05) positive correlation with soil Fe, Se and Zn. Selenium concentration in MO leaves showed a stronger significant (*p* < 0.05) positive correlation with phosphate extractable (Se-P) soil Se than the total soil Se (S23 and S24 Tables). The Ca and Zn concentration in MO leaves showed no statistically significant (*p* ≥ 0.05) correlation with any of the reported soil properties.

*Moringa stenopetala* leaf Fe concentration was significantly (*p* < 0.05) positively correlated with soil Mg, Se and Se-P. Similarly, MS leaves Mg concentration showed a statistically significant (*p* <0.05) negative correlation with soil Zn concentration. The Se content of MS leaves indicated a strong and significant positive correlation with the soil Se-P (S25 and S26 Tables). However, the Ca, Cu and I concentration of MS leaves did not show significant (*p* ≥ 0.05) correlation with any of the soil properties.

## Discussion

### Elemental concentration in edible parts of *Moringa* spp.

This study is the first comprehensive analysis of Se concentrations in different edible parts of MO and MS grown in various localities. Four previous studies reported Se concentrations in MO leaves, from Niger (27.1 mg kg^-1^ dw) [46], Solomon Islands (2 mg kg^-1^ dw) [42], South Africa (363 mg kg^-1^ dw) [41], and Mexico, Lombardia (0.096 mg kg^-1^ dw) and San Pedro (1.07 mg kg^-1^ dw) [47]. Our MO leaf Se concentration (mean = 4.25 and median = 2.73 mg kg^-1^ dw) is consistent with results from Solomon Islands and Mexico, but differ markedly from the South African data. The results of the analyses from Niger were based on two samples. Taking this into account, and the fact that a MO leaf sample (L-MO-29-MBO) from Mbololo, Kenya, had a mean Se concentration of 21.2 mg kg^-1^ dw from triplicate analyses (S34 Table), the findings from Niger were reasonably consistent with ours. Our attempt to verify the very high reported concentrations from the South Africa study by contacting the authors was not successful. Iodine concentrations in MO and MS leaves or other parts have not been reported previously to our knowledge.

Table 4 summarises 11 previous studies of MO leaf elemental concentrations alongside data from the present study. Allowing for differences in analytical method and likely inter-study variation in leaf maturity there are many broad similarities. For example, the mean Ca concentration in MO leaves in the present study is the fourth lowest following MO leaf samples collected from Kuje, Nigeria [43], Hawassa, Ethiopia [48] and Jalisco state, Mexico [4], while the mean Zn concentration is the second highest following MO leaves collected from Thailand [49]. Similarly, mean elemental concentrations in MS leaves collected from Ethiopia and Kenya in the current study indicated inconsistent variation when compared with previous studies. For instance, the mean concentration of Ca in MS leaves of 21 g kg^-1^ dw in the present study is comparable with that reported from Ethiopia [40] (19.8 g kg^-1^ dw), and greater than the 12.7 g kg^-1^ dw reported from Mexico [4]. However, the Fe concentration (162 mg kg^-1^ dw) in the present study is far lower than that reported from Ethiopia (666 mg kg^-1^ dw) [40].

**Table 4:** Comparison of reported mineral element concentrations in MO leaf, sources, number of observation (n) and locations. The concentration values in the first row in bold are the result from current study.

Ca	Cu	I	Fe	Mg	Se	Zn	Source	n	Location
mean (mg kg^-1^ dw)									
**18300**	**6.92**	**0.218**	**202**	**5370**	**4.25**	**35.6**	**Current study**	**56**	**Various localities, Kenya**
16000	9.6	__	97.9	2800	__	29.1	[4]	23	Mexico, Jalisco State
25800	9.44	__	591	5520	__	24.7	[40] ^†^	6	Hawassa, Ethiopia
26200	9.58	__	561	5550	__	25.2	[40]^†^	6	Arbaminch, Ethiopia
36500	8.25	__	490	5000	363	31	[41]		Limpopo, South Africa
20000	7	__	___	3700	2.0	31	[42]		Honaiara, Solomon Islands
3463	44	__	41	725	__	__	[43]	2	Kuje, Abuja, Nigeria
38270	43.6	__	78.8	806	__	__	[43]	2	Sheda, Abuja, Nigeria
22900	9.5	__	205	100	__	25.9	[44]	2	Bahawalnager, Pakistan
19000	11.2	__	397	98.2	__	20.9	[44]	2	Sadiqabad, Pakistan
26400	7.3	__	573	109	__	34.1	[44]	2	Chenabnager, Pakistan
24000	8.85	__	226	4340	27.1	< 5	[46]	2	Zinger, Niger
20200	10.3	__	194	3230	0.096	10	[47]	5	Lombardia, Mexico
26200	4.1	__	70.7	3400	1.07	16	[47]	5	San Pedro, Mexico
12900	17.7	__	391	1800	__	28.2	[48]		Hawassa, Ethiopia
__	7.6	__	82.2	__	__	92.8	[49]^†^	5	Thailand
18400		__	173	5630	__	24.8	[50]		Chad
27400	12.2	__	417	4900	__	30.9	[50]		Sahrawi camps, Algeria
21500	6.6	__	119	5340	__	21.8	[50]		Haiti

^†^Values were averaged

Mean elemental concentrations in MS leaves collected from Ethiopia and Kenya in the current study indicated inconsistent variation when compared with previous studies. For instance, the mean concentration of Ca in MS leaves of 21 g kg^-1^ dw in the present study is comparable with that reported from Ethiopia [40] (19.8 g kg^-1^ dw), and greater than the 12.7 g kg^-1^ dw reported from Mexico [4]. However, the Fe concentration (162 mg kg^-1^ dw) in the present study is far lower than that reported from Ethiopia (666 mg kg^-1^ dw) [40].

The mean concentration of Ca (3,600 mg kg^-1^ dw) in the immature pods of MO in the current study was higher than that reported from Ethiopia (2,740 mg kg^-1^ dw) [40] and Pakistan (2,740 mg kg^-1^ dw) [44]. However, the Fe concentration (65.4 mg kg^-1^ dw) in the MO immature pods in this study was much lower than that reported from Ethiopia (510 mg kg^-1^ dw) [40] and Pakistan (510 mg kg^-1^ dw) [44]. The Cu, Mg and Zn concentrations in the immature pods of MO reported from Ethiopia [40] are comparable to our findings. However, the concentration of Cu in the immature pods of MO reported from Pakistan (26.6 mg kg^-1^ dw) [44] was higher than the results in this study (5.42 mg kg^-1^ dw). Elemental concentration in MO seed kernel in the present study showed inconsistent variation as compared to previous studies in two regions of Nigeria [43]. For example, the mean concentration of Cu (4.2 mg kg^-1^ dw) and Fe (49.2 mg kg^-1^ dw) in MO seed kernel in the present study is lower than the MO seed kernel samples collected from Sheda region of Nigeria with Cu and Fe concentration of 34.2 mg kg^-1^ dw and 118.5 mg kg^-1^ dw, respectively. Calcium concentration of 1310 mg kg^-1^ dw in the present study is higher than the concentration of Ca (1029 mg kg^-1^) in MO seed kernel collected from Kuje region of Nigeria [43]. The variation in elemental concentration in immature pods and seeds of MO this study and others can be attributed to the variation in environment in which MO grew, intra-specific variation in the MO, the variation in maturity levels of immature pods, and the difference in analytical method pursued. There are no studies we are aware of that report the elemental concentrations in the flowers of *Moringa* species.

### Variation in *Moringa* spp. element concentration

Variation in the elemental concentrations of the different edible parts of MO and MS can be due to the impact of the environment/management, the effect of intra- and inter-specific genetic variation [4], and the interactions between the genetics and environment [40]. The seeds and/or planting materials sources of the *Moringa* trees from which the edible parts were sampled were not traceable and so this discussion is limited to environment/management factors. *S*amples were collected from trees that were grown and managed by households in various localities of Ethiopia and Kenya. Different households pursue various tree management regimes, such as, lone trees, hedgerow, woodlot, pollarding, lopping, watering, fertilizing, intercropping, etc. For example, some households place household wastes and manure that can supply nutrients to the trees and some may water their plants during dry season. Stages of growth, for instance, of the leaves at the time of surveying varies due to variation in management regime, and climate and soil type may all contribute further to variation of the elemental concentration in the edible parts.

The positive correlation between some of the *Moringa* edible parts elemental concentration and soil chemical properties indicate the significance of the soil environment in which the plants grow besides the inherent genetic ability of these species to absorb and translocate mineral elements to edible parts. In addition, it is not only the quantity of the mineral element available in the soil which impacts on the *Moringa* spp. edible parts elemental concentration but also the chemical form in which the element exists in the soil [51]. The stronger positive correlation of *Moringa* leaves Se concentration with KH_2_PO_4_ extractable soil Se than the total soil Se was an indication of the association between *Moringa* edible parts and phyto-available soil elemental concentration.

### *Moringa* spp. role in human Se nutrition

Selenium deficiencies are widespread in sub-Saharan Africa [3, 33]. For instance, based on 2009 food supply data from the Food and Agriculture Organization, national level Se deficiency risks in Ethiopia and Kenya were estimated to be 35.5% and 58.3%, respectively [33]. Based on seven day dietary recall survey conducted in the year 2010–2011, Joy, Kumssa *et al*., [3] estimated that 81% of Malawian households had insufficient Se to meet dietary requirements. Similarly, in northwest Ethiopia, Gonder town, a cross-sectional study on school children (n = 100) using blood serum concentration of mineral nutrients reported 62% of the children were deficient in Se [52]. Gashu *et al*. [53] reported Se deficiency risk in school children in the Amhara region of Ethiopia to be 58% (n = 349).

*Moringa* spp. edible parts contain high concentrations of Se and the leaves have similar levels of the 6 other reported mineral elements to other leafy vegetables grown in the same localities. Table 5 summarizes the Recommended Daily Allowances (RDA) for an adult male of Ca, Cu, I, Fe, Mg, Se and Zn, concentrations of these mineral elements in MO and MS leaves collected from various localities of Ethiopia and Kenya, and percentage of RDA fulfilled by consuming 100 g of fresh *Moringa* leaves per day. The RDA is a daily nutrient intake level that fulfils the nutrient requirements of ~ 98% of the healthy individuals in an age- and sex-specific population [54]. *Moringa oleifera* grown without Se fertilizer can provide 100% of the RDA of a healthy adult man which is comparable with Se obtained from a similar quantity of carrots biofortified with 1 kg ha^-1^ of Se fertilizer [55], and maize biofortified with 5 g of Se ha^-1^ at the level of the Malawian population maize consumption [56]. A daily consumption of 100 g fresh leaves of MS grown in Ethiopia can fulfil 41% of the Se RDA, while MS grown in Kenya can provide 144% of the Se RDA for a healthy adult man. Consumption of fresh leaves or leaf powders of MO and MS, for example, can help at least to reduce the many MNDs and alleviate Se deficiency if interventions target vulnerable populations living in localities where these *Moringa* species grow vigorously. Besides, *Moringa* leaf powders can be stored for use during the dry season and transported and traded with areas where *Moringa* is not cultivated to fight against MNDs. In areas where rain fed agriculture is practiced, other vegetables, for example, *Brassica* can be used to diversify sources of dietary mineral elements and *Moringa* leaf powders can be stored and used when they are needed most during the dry season.

**Table 5:** Recommended Daily Allowance (RDA) [54] for 19–70 yrs. old adult males (mg capita^-1^ d^-1^), median elemental concentration in 100 g fresh *Moringa* leaves (mg) from Kenya and Ethiopia and percentage of RDA fulfilled by consuming 100 g fresh *Moringa* leaves.

	Ca	Cu	I	Fe	Mg	Se	Zn
**RDA**	1000	0.9	0.15	18	320	0.055	8
**MO Kenya**	334	0.137	0.004	3.18	108	0.055	0.665
**% of RDA fulfilled**	33	15	3	18	34	100	8
**MS Ethiopia**	387	0.094	0.002	2.34	121	0.022	0.419
**% of RDA fulfilled**	39	10	1	13	38	41	5
**MS Kenya**	450	0.061	0.005	3.80	144	0.079	0.314
**% of RDA fulfilled**	45	7	3	21	45	144	4

## Conclusion

In addition to the high selenium concentration, *Moringa* spp. leaves are rich in proteins and β-carotene [4, 50], possess anti-oxidant properties [17], contain low concentrations of anti-nutrients [57–60], may be used in treating ailments [18, 61], the seeds are used as water coagulant [62], and they grow under marginal environmental conditions providing much needed ecological services (for example, shade, wind break, etc.). *Moringa oleifera* is naturalized while MS is indigenous to Kenya and Ethiopia. Where these species grow, the population have indigenous knowledge of their multiple uses including the high nutritive values [63]. Nonetheless, the utilization of these species as food is limited to specific localities and communities [8], they are neglected in terms of research and development, and the trees can be classed as underutilized crops [64, 65]. Agricultural and health extension work to popularise the production and consumption of MO and MS may be a useful strategy to complement efforts to alleviate dietary MNDs through dietary diversification, and use of *Moringa* leaf powders to fortify meals in the dry season when other leafy green vegetables are not available. In addition, variations in mineral micronutrient concentrations suggest that breeding efforts to increase the nutritional value of *Moringa* foliage may be successful. However, research is also required to determine the bioavailability of nutrients from *Moringa* edible parts. The Moringaceae belongs to the same order (Brassicales) as Brassicaceae [5] which are known to be Se accumulators [66, 67]. Hence further studies on the Se concentrations in edible portions of the other *Moringa* species are important to understand and exploit the potential of the family in the fight against human Se undernutrition.

## Supporting information

S1 Table Number of *Moringa* edible part samples collected from Ethiopia and Kenya by locality and species. MO, *M*. *oleifera*, MS, *M*. *stenopetala*.
(PDF)

S2 Table Number of soil samples (n) collected from the different localities in Ethiopia and Kenya. (PDF)

S3 Table Descriptive statistics on elemental concentration (mg kg^-1^) of plant Certified Reference Materials (CRM). (PDF)

S4 Table Descriptive statistics on elemental concentration (mg kg^-1^) of soil Certified Reference Materials (CRM) (2711A). (PDF)

S5 Table Shapiro-Wilk test of normality of the distribution of soil elemental concentration by locality. (PDF)

S6 Table Levene’s test of homogeneity of variances of soil elemental concentration based on mean and median. D.f. 1 is the degree of freedom of the numerator, and d.f. 2 is the degree of freedom of the denominator.
(PDF)

S7 Table Test of normality of the distribution of MO leaves elemental concentration by locality in Kenya. (PDF)

S8 Table Levene’s test of homogeneity of variances of MO leaves elemental concentration by localities in Kenya based on mean and median. D.f. 1 is the degree of freedom of the numerator, and d.f. 2 is the degree of freedom of the denominator.
(PDF)

S9 Table Test of normality of the distribution of MS leaves elemental concentration by locality. (PDF)

S10 Table Levene’s test of homogeneity of variances of MS leaves elemental concentration by localities based on mean and median. D.f. 1 is the degree of freedom of the numerator, and d.f. 2 is the degree of freedom of the denominator.
(PDF)

S11 Table Test of normality of the distribution of MO immature pods elemental concentration by locality. (PDF)

S12 Table Levene’s test of homogeneity of variances of MO immature pods elemental concentration by localities. (PDF)

S13 Table Test of normality of the distribution of MO seeds elemental concentration by locality. (PDF)

S14 Table Levene’s test of homogeneity of variances of MO seeds elemental concentration by localities. (PDF)

S15 Table Test of normality of the distribution of MO flowers elemental concentration by locality. (PDF)

S16 Table Levene’s test of homogeneity of variances of MO flowers elemental concentration by localities. (PDF)

S17 Table Descriptive statistics of MO leaves elemental concentration (mg kg-1) by locality. (PDF)

S18 Table Descriptive statistics for MS leaves elemental concentration (mg kg-1) by locality. (PDF)

S19 Table Descriptive statistics for MO immature pods elemental concentration (mg kg-1) by locality. (PDF)

S20 Table Descriptive statistics for MO seeds elemental concentration (mg kg-1) by locality. (PDF)

S21 Table Descriptive statistics for MO flowers elemental concentration (mg kg-1) by locality. (PDF)

S22 Table Descriptive statistics for soil elemental concentration (mg kg-1) and pH by locality. (PDF)

S23 Table Spearman’s rank correlation (N = 56, d.f. = 54) between the elemental concentration of MO leaves and soil properties. (PDF)

S24 Table The *t* probabilities for the Spearman’s rank correlation between the elemental concentration of MO leaves and soil properties. Significant correlations are in bold.
(PDF)

S25 Table Spearman’s rank correlation (N = 32, d.f. = 30) between the elemental concentration of MS leaves and soil properties. (PDF)

S26 Table The t probabilities for the Spearman’s rank correlation between the elemental concentration of MS leaves and soil properties. Significant correlations are in bold.
(PDF)

S27 Table Welch’s robust test of equality of mean elemental concentrations in MO leaves across localities in Kenya. d.f. 1 (degrees of freedom of the numerator), d.f. 2 (degrees of freedom of the denominator), and the *p* probability value.
(PDF)

S28 Table Welch’s robust test of equality of mean elemental concentrations in MS leaves across localities. d.f. 1 (degrees of freedom of the numerator), d.f. 2 (degrees of freedom of the denominator), and the *p* (probability value).
(PDF)

S29 Table Welch’s robust test of equality of mean elemental concentrations in MO and MS leaves. d.f. 1 (degrees of freedom of the numerator), d.f. 2 (degrees of freedom of the denominator), and the *p* (probability value).
(PDF)

S30 Table Welch’s robust test of equality of mean elemental concentrations in MO immature pods across localities. d.f. 1 (degrees of freedom of the numerator), d.f. 2 (degrees of freedom of the denominator), and the *p* (probability value).
(PDF)

S31 Table Welch’s robust test of equality of mean elemental concentrations in MO seeds across localities. d.f. 1 (degrees of freedom of the numerator), d.f. 2 (degrees of freedom of the denominator), and the *p* (probability value).
(PDF)

S32 Table Welch’s robust test of equality of mean elemental concentrations in MO flowers across localities. d.f. 1 (degrees of freedom of the numerator), d.f. 2 (degrees of freedom of the denominator), and the *p* (probability value).
(PDF)

S33 Table Welch’s robust tests of equality of mean soil elemental concentrations across localities. d.f. 1 (degrees of freedom of the numerator), d.f. 2 (degrees of freedom of the denominator), and the *p* (probability value).
(PDF)

S34 Table Raw data on MO and MS edible parts elemental concentration (mg kg^-1^), and sample details. (PDF)

S35 Table Raw data on MO and MS leaves iodine concentration (mg kg^-1^) and sample details. (PDF)

S36 Table Raw data on elemental concentrations (mg kg^-1^) in various crops and sample details. ND = not detectable.
(PDF)

S37 Table Raw data on soil elemental concentration (mg kg^-1^) and pH, and sample details. (PDF)

S38 Table Raw data on soil iodine concentration (mg kg^-1^) and sample details. (PDF)

S39 Table Raw data on phosphate-extractable soil selenium (Se-P) concentration (mg kg-1) and sample details. (PDF)

S40 Table Correlation between elemental concentrations of MO edible parts (flower, immature pod, leaf and seed). The figures below the yellow diagonal are correlation coefficients and those above the diagonal are p values. ** Correlation is significant at the 0.01 level (2-tailed). * Correlation is significant at the 0.05 level (2-tailed). N = 18
(PDF)

S41 Table Correlation between the elemental composition of MO and amaranth leaves. * Correlation is significant at the 0.05 level (2-tailed). N = 6.
(PDF)

S42 Table Correlation between the elemental composition of MO and brassica (BO) leaves. ** Correlation is significant at the 0.05 level (2-tailed). N = 4.
(PDF)

S43 Table Correlation between the elemental composition of MO and brassica (BO) leaves. ** Correlation is significant at the 0.05 level (2-tailed). N = 3.
(PDF)
