# Supplementary material for: Variation in the mineral element concentration of Moringa oleifera Lam. and M. stenopetala (Bak. f.) Cuf.: Role in human nutrition
Source: PLoS One. 2017 Apr 7;12(4):e0175503. doi: 10.1371/journal.pone.0175503 (PMC5384779; doi:10.1371/journal.pone.0175503)
Supplement: S1 Table — MO, M. oleifera, MS, M. stenopetala. (PDF) [file pone.0175503.s001.pdf]

S1 Table. Number of *Moringa* edible part samples collected from Ethiopia and Kenya by locality and species. MO, *M. oleifera*, MS, *M. stenopetala.*

Species	Country	Locality	Flower	Immature pod	Leaf	Root	Seed
MO	Kenya	Baringo	1				
	Kenya	Kibwezi	1	4	14		9
	Kenya	Malindi	7	2	11		2
	Kenya	Mbololo	16	13	16		16
	Kenya	Ramogi	7	5	8		7
	Kenya	Ukunda	3	3	7		1
MS	Kenya	Baringo	3	1	5	1	2
	Ethiopia	Derashe	1	1	12		
	Ethiopia	Hawasa			14		
	Ethiopia	Konso			16		1
	Kenya	Ramogi			1		
