# Supplementary material for: Variation in the mineral element concentration of Moringa oleifera Lam. and M. stenopetala (Bak. f.) Cuf.: Role in human nutrition
Source: PLoS One. 2017 Apr 7;12(4):e0175503. doi: 10.1371/journal.pone.0175503 (PMC5384779; doi:10.1371/journal.pone.0175503)
Supplement: S2 Table — (PDF) [file pone.0175503.s002.pdf]

S2 Table. Number of soil samples (n) collected from the different localities in Ethiopia and Kenya.

Country	Locality	n
Ethiopia	Derashe	12
	Hawasa	9
	Konso	12
Kenya	Baringo	6
	Kibwezi	14
	Malindi	11
	Mbololo	16
	Ramogi	8
	Ukunda	7
