# Supplementary material for: Variation in the mineral element concentration of Moringa oleifera Lam. and M. stenopetala (Bak. f.) Cuf.: Role in human nutrition
Source: PLoS One. 2017 Apr 7;12(4):e0175503. doi: 10.1371/journal.pone.0175503 (PMC5384779; doi:10.1371/journal.pone.0175503)
Supplement: S3 Table — (PDF) [file pone.0175503.s003.pdf]

S3 Table. Descriptive statistics on elemental concentration (mg kg^{-1}) of plant Certified Reference Materials (CRM).

	1567B						1573A					
**Element**	**Ca**	**Cu**	**Fe**	**Mg**	**Se**	**Zn**	**Ca**	**Cu**	**Fe**	**Mg**	**Se**	**Zn**
**N**	8	8	8	8	8	8	7	7	7	7	7	7
**Mean**	189.700	1.700	12.910	333.400	1.130	10.340	51066.000	4.350	333.400	11162.000	0.080	30.460
**Median**	189.400	1.870	12.810	327.500	1.100	10.050	53182.000	4.470	348.300	11600.000	0.080	31.600
**Minimum**	169.200	0.000	11.920	302.800	1.060	9.270	38332.000	3.330	246.700	8443.000	0.060	23.630
**Maximum**	205.100	2.260	14.840	362.800	1.230	11.530	55428.000	4.940	373.100	12177.000	0.090	33.640
**Lower quartile**	182.700	1.690	12.150	317.800	1.070	9.670	50338.000	4.240	320.000	11018.000	0.070	29.310
**Upper quartile**	199.700	2.100	13.290	355.600	1.200	11.250	54173.000	4.580	360.900	11836.000	0.090	32.740
**Standard deviation**	11.990	0.720	0.950	22.220	0.070	0.890	5916.000	0.500	42.690	1266.000	0.010	3.400
**Standard error of mean**	4.240	0.250	0.340	7.860	0.020	0.310	2236.000	0.190	16.130	478.500	0.000	1.280
**Coefficient of variation**	6.320	42.290	7.360	6.670	6.170	8.560	11.590	11.540	12.800	11.340	13.070	11.150
