# Supplementary material for: Variation in the mineral element concentration of Moringa oleifera Lam. and M. stenopetala (Bak. f.) Cuf.: Role in human nutrition
Source: PLoS One. 2017 Apr 7;12(4):e0175503. doi: 10.1371/journal.pone.0175503 (PMC5384779; doi:10.1371/journal.pone.0175503)
Supplement: S4 Table — (PDF) [file pone.0175503.s004.pdf]

S4 Table. Descriptive statistics on elemental concentration (mg kg^{-1}) of soil Certified Reference Materials (CRM) (2711A)

Element	Ca	Cu	I	Fe	Mg	Se	Se-P	Zn
N	3	3	3	3	3	3	3	3
Mean	19,501.000	100.700	1.060	25,678.000	7,928.000	1.800	0.140	334.900
Median	19,934.000	99.700	1.060	26,340.000	8,182.000	1.840	0.140	337.200
Minimum	17,684.000	93.870	1.050	23,376.000	7,096.000	1.620	0.130	308.900
Maximum	20,885.000	108.400	1.060	27,319.000	8,506.000	1.920	0.140	358.700
Lower quartile	18,246.000	95.330	1.050	24,117.000	7,368.000	1.680	0.130	316.000
Upper quartile	20,648.000	106.200	1.060	27,075.000	8,425.000	1.900	0.140	353.300
Standard deviation	1,644.000	7.310	0.010	2,053.000	738.100	0.160	0.000	24.960
Standard error of mean	949.200	4.220	0.010	1,186.000	426.200	0.090	0.000	14.410
Coefficient of variation	8.430	7.260	0.890	8.000	9.310	8.670	1.940	7.450
