# Supplementary material for: Variation in the mineral element concentration of Moringa oleifera Lam. and M. stenopetala (Bak. f.) Cuf.: Role in human nutrition
Source: PLoS One. 2017 Apr 7;12(4):e0175503. doi: 10.1371/journal.pone.0175503 (PMC5384779; doi:10.1371/journal.pone.0175503)
Supplement: S5 Table — (PDF) [file pone.0175503.s005.pdf]

S5 Table. Shapiro-Wilk test of normality of the distribution of soil elemental concentration by locality.

Element	Locality	Shapiro-Wilk Statistic	d.f.	*p*
Ca	Derashe	0.83	12	0.021
	Hawassa	0.886	9	0.18
	Konso	0.842	12	0.03
	Baringo	0.783	6	0.041
	Kibwezi	0.859	14	0.029
	Malindi	0.788	11	0.007
	Mbololo	0.982	16	0.977
	Ramogi	0.966	8	0.862
	Ukunda	0.941	7	0.644
Cu	Derashe	0.899	12	0.156
	Hawassa	0.502	9	0
	Konso	0.805	12	0.011
	Baringo	0.864	6	0.203
	Kibwezi	0.903	14	0.125
	Malindi	0.903	11	0.199
	Mbololo	0.946	16	0.423
	Ramogi	0.749	8	0.008
	Ukunda	0.855	7	0.137
I	Derashe	0.892	12	0.126
	Hawassa	0.823	9	0.037
	Konso	0.909	12	0.207
	Baringo	0.938	6	0.646
	Kibwezi	0.889	14	0.078
	Malindi	0.768	11	0.004
	Mbololo	0.926	16	0.207
	Ramogi	0.956	8	0.768
	Ukunda	0.941	7	0.649
	Derashe	0.921	12	0.297
	Hawassa	0.864	9	0.105

Element	Locality	Shapiro-Wilk Statistic	d.f.	*p*
Fe	Konso	0.895	12	0.137
	Baringo	0.933	6	0.603
	Kibwezi	0.908	14	0.146
	Malindi	0.861	11	0.058
	Mbololo	0.944	16	0.407
	Ramogi	0.762	8	0.011
	Ukunda	0.884	7	0.244
Mg	Derashe	0.817	12	0.015
	Hawassa	0.852	9	0.078
	Konso	0.869	12	0.063
	Baringo	0.812	6	0.075
	Kibwezi	0.847	14	0.02
	Malindi	0.952	11	0.673
	Mbololo	0.966	16	0.768
	Ramogi	0.871	8	0.155
	Ukunda	0.719	7	0.006
Se	Derashe	0.886	12	0.104
	Hawassa	0.862	9	0.102
	Konso	0.891	12	0.123
	Baringo	0.911	6	0.444
	Kibwezi	0.945	14	0.487
	Malindi	0.973	11	0.914
	Mbololo	0.846	16	0.012
	Ramogi	0.978	8	0.953
	Ukunda	0.874	7	0.2
Se-P	Derashe	0.948	12	0.602
	Hawassa	0.922	9	0.411
	Konso	0.839	12	0.027
	Baringo	0.869	6	0.222
	Kibwezi	0.938	14	0.391
	Malindi	0.874	11	0.089
	Mbololo	0.875	16	0.033

Element	Locality	Shapiro-Wilk Statistic	d.f.	*p*
	**Ramogi**	0.724	8	0.004
	**Ukunda**	0.948	7	0.714
**Zn**	**Derashe**	0.951	12	0.648
	**Hawassa**	0.801	9	0.021
	**Konso**	0.938	12	0.472
	**Baringo**	0.911	6	0.446
	**Kibwezi**	0.92	14	0.219
	**Malindi**	0.901	11	0.191
	**Mbololo**	0.971	16	0.849
	**Ramogi**	0.942	8	0.633
	**Ukunda**	0.877	7	0.212
**pH**	**Derashe**	0.809	12	0.012
	**Hawassa**	0.868	9	0.118
	**Konso**	0.922	12	0.299
	**Baringo**	0.88	6	0.269
	**Kibwezi**	0.955	14	0.645
	**Malindi**	0.905	11	0.215
	**Mbololo**	0.959	16	0.642
	**Ramogi**	0.954	8	0.747
	**Ukunda**	0.668	7	0.002
