# Supplementary material for: Variation in the mineral element concentration of Moringa oleifera Lam. and M. stenopetala (Bak. f.) Cuf.: Role in human nutrition
Source: PLoS One. 2017 Apr 7;12(4):e0175503. doi: 10.1371/journal.pone.0175503 (PMC5384779; doi:10.1371/journal.pone.0175503)
Supplement: S6 Table — D.f. 1 is the degree of freedom of the numerator, and d.f. 2 is the degree of freedom of the denominator. (PDF) [file pone.0175503.s006.pdf]

S6 Table. Levene's test of homogeneity of variances of soil elemental concentration based on mean and median. D.f. 1 is the degree of freedom of the numerator, and d.f. 2 is the degree of freedom of the denominator.

Element		Levene Statistic	d.f. 1	d.f. 2	*p*
Ca	Based on Mean	11.233	8	86	0
	Based on Median	7.967	8	86	0
	Based on Median and with adjusted d.f.	7.967	8	60	0
	Based on trimmed mean	10.324	8	86	0
Cu	Based on Mean	8.078	8	86	0
	Based on Median	2.696	8	86	0.011
	Based on Median and with adjusted d.f.	2.696	8	24	0.029
	Based on trimmed mean	7.008	8	86	0
I	Based on Mean	6.125	8	86	0
	Based on Median	4.875	8	86	0
	Based on Median and with adjusted d.f.	4.875	8	29	0.001
	Based on trimmed mean	5.955	8	86	0
Fe	Based on Mean	10.434	8	86	0
	Based on Median	4.888	8	86	0
	Based on Median and with adjusted d.f.	4.888	8	21	0.002
	Based on trimmed mean	9.136	8	86	0
Mg	Based on Mean	14.734	8	86	0
	Based on Median	11.067	8	86	0
	Based on Median and with adjusted d.f.	11.067	8	38	0
	Based on trimmed mean	13.41	8	86	0
Se	Based on Mean	7.215	8	86	0
	Based on Median	5.379	8	86	0
	Based on Median and with adjusted d.f.	5.379	8	69	0
	Based on trimmed mean	6.984	8	86	0
Se-P	Based on Mean	11.445	8	86	0
	Based on Median	8.481	8	86	0
	Based on Median and with adjusted d.f.	8.481	8	66	0
	Based on trimmed mean	10.997	8	86	0
	Based on Mean	6.206	8	86	0

Element		Levene Statistic	d.f. 1	d.f. 2	*p*
Zn	**Based on Median**	4.393	8	86	0
	**Based on Median and with adjusted d.f.**	4.393	8	10	0.015
	**Based on trimmed mean**	5.216	8	86	0
pH	**Based on Mean**	3.463	8	86	0.002
	**Based on Median**	2.658	8	86	0.012
	**Based on Median and with adjusted d.f.**	2.658	8	46	0.017
	**Based on trimmed mean**	3.389	8	86	0.002
