# Supplementary material for: Variation in the mineral element concentration of Moringa oleifera Lam. and M. stenopetala (Bak. f.) Cuf.: Role in human nutrition
Source: PLoS One. 2017 Apr 7;12(4):e0175503. doi: 10.1371/journal.pone.0175503 (PMC5384779; doi:10.1371/journal.pone.0175503)
Supplement: S7 Table — (PDF) [file pone.0175503.s007.pdf]

S7 Table. Test of normality of the distribution of MO leaves elemental concentration by locality in Kenya.

Element	Locality	Shapiro-Wilk Statistic	d.f.	*p*
Ca	Kibwezi	0.906	14	0.136
	Malindi	0.895	11	0.162
	Mbololo	0.918	16	0.156
	Ramogi	0.815	8	0.041
	Ukunda	0.74	7	0.010
Cu	Kibwezi	0.972	14	0.906
	Malindi	0.866	11	0.068
	Mbololo	0.94	16	0.344
	Ramogi	0.961	8	0.817
	Ukunda	0.886	7	0.256
I	Kibwezi	0.702	14	0.000
	Malindi	0.486	11	0.000
	Mbololo	0.919	16	0.160
	Ramogi	0.708	8	0.003
	Ukunda	0.762	7	0.017
Fe	Kibwezi	0.916	14	0.192
	Malindi	0.929	11	0.397
	Mbololo	0.79	16	0.002
	Ramogi	0.643	8	0.000
	Ukunda	0.881	7	0.229
Mg	Kibwezi	0.968	14	0.852
	Malindi	0.81	11	0.013
	Mbololo	0.884	16	0.045
	Ramogi	0.944	8	0.650
	Ukunda	0.867	7	0.175
Zn	Kibwezi	0.904	14	0.129
	Malindi	0.948	11	0.615
	Mbololo	0.951	16	0.502
	Ramogi	0.929	8	0.509
	Ukunda	0.957	7	0.791

Element	Locality	Shapiro-Wilk Statistic	d.f.	*p*
**Se**	**Kibwezi**	0.87	14	0.043
	**Malindi**	0.656	11	0.000
	**Mbololo**	0.777	16	0.001
	**Ramogi**	0.808	8	0.035
	**Ukunda**	0.735	7	0.009
