# Supplementary material for: Variation in the mineral element concentration of Moringa oleifera Lam. and M. stenopetala (Bak. f.) Cuf.: Role in human nutrition
Source: PLoS One. 2017 Apr 7;12(4):e0175503. doi: 10.1371/journal.pone.0175503 (PMC5384779; doi:10.1371/journal.pone.0175503)
Supplement: S8 Table — D.f. 1 is the degree of freedom of the numerator, and d.f. 2 is the degree of freedom of the denominator. (PDF) [file pone.0175503.s008.pdf]

S8 Table. Levene's test of homogeneity of variances of MO leaves elemental concentration by localities in Kenya based on mean and median. D.f. 1 is the degree of freedom of the numerator, and d.f. 2 is the degree of freedom of the denominator.

Element		Levene Statistic	d.f. 1	d.f. 2	*p*
Ca	**Based on Mean**	1.402	4	51	0.247
	**Based on Median**	0.585	4	51	0.675
	**Based on Median and with adjusted df**	0.585	4	22	0.677
	**Based on trimmed mean**	1.146	4	51	0.346
Cu	**Based on Mean**	1.18	4	51	0.331
	**Based on Median**	1.06	4	51	0.386
	**Based on Median and with adjusted df**	1.06	4	37	0.390
	**Based on trimmed mean**	1.164	4	51	0.338
I	**Based on Mean**	8.953	4	51	0.000
	**Based on Median**	1.835	4	51	0.136
	**Based on Median and with adjusted df**	1.835	4	14	0.177
	**Based on trimmed mean**	5.764	4	51	0.001
Fe	**Based on Mean**	4.08	4	51	0.006
	**Based on Median**	2.673	4	51	0.042
	**Based on Median and with adjusted df**	2.673	4	10	0.093
	**Based on trimmed mean**	3.209	4	51	0.020
Mg	**Based on Mean**	0.045	4	51	0.996
	**Based on Median**	0.089	4	51	0.985
	**Based on Median and with adjusted df**	0.089	4	40	0.985
	**Based on trimmed mean**	0.057	4	51	0.994
Zn	**Based on Mean**	4.831	4	51	0.002
	**Based on Median**	4.157	4	51	0.005
	**Based on Median and with adjusted df**	4.157	4	35	0.007
	**Based on trimmed mean**	4.729	4	51	0.003
Se	**Based on Mean**	2.582	4	51	0.048
	**Based on Median**	1.527	4	51	0.208
	**Based on Median and with adjusted df**	1.527	4	33	0.217
	**Based on trimmed mean**	2.19	4	51	0.083
