# Supplementary material for: Variation in the mineral element concentration of Moringa oleifera Lam. and M. stenopetala (Bak. f.) Cuf.: Role in human nutrition
Source: PLoS One. 2017 Apr 7;12(4):e0175503. doi: 10.1371/journal.pone.0175503 (PMC5384779; doi:10.1371/journal.pone.0175503)
Supplement: S9 Table — (PDF) [file pone.0175503.s009.pdf]

S9 Table. Test of normality of the distribution of MS leaves elemental concentration by locality.

Element	Locality	Shapiro-Wilk statistic	d.f.	*P*
Ca	Derashe	0.941	8	0.622
	Hawassa	0.846	14	0.019
	Konso	0.969	14	0.87
	Baringo	0.901	5	0.417
Cu	Derashe	0.935	8	0.558
	Hawassa	0.964	14	0.785
	Konso	0.927	14	0.274
	Baringo	0.958	5	0.795
I	Derashe	0.804	8	0.032
	Hawassa	0.896	14	0.1
	Konso	0.721	14	0.001
	Baringo	0.846	5	0.182
Fe	Derashe	0.941	8	0.619
	Hawassa	0.9	14	0.111
	Konso	0.447	14	0
	Baringo	0.873	5	0.277
Mg	Derashe	0.967	8	0.871
	Hawassa	0.963	14	0.776
	Konso	0.913	14	0.174
	Baringo	0.798	5	0.078
Se	Derashe	0.691	8	0.002
	Hawassa	0.962	14	0.758
	Konso	0.728	14	0.001
	Baringo	0.946	5	0.709
Zn	Derashe	0.65	8	0.001
	Hawassa	0.903	14	0.126
	Konso	0.975	14	0.94
	Baringo	0.98	5	0.933
