# Supplementary material for: Variation in the mineral element concentration of Moringa oleifera Lam. and M. stenopetala (Bak. f.) Cuf.: Role in human nutrition
Source: PLoS One. 2017 Apr 7;12(4):e0175503. doi: 10.1371/journal.pone.0175503 (PMC5384779; doi:10.1371/journal.pone.0175503)
Supplement: S10 Table — D.f. 1 is the degree of freedom of the numerator, and d.f. 2 is the degree of freedom of the denominator. (PDF) [file pone.0175503.s010.pdf]

S10 Table. Levene's test of homogeneity of variances of MS leaves elemental concentration by localities based on mean and median. D.f. 1 is the degree of freedom of the numerator, and d.f. 2 is the degree of freedom of the denominator.

Element		Levene Statistic	d.f. 1	d.f. 2	*P*
Ca	Based on Mean	5.154	3	37	0.004
	Based on Median	2.404	3	37	0.083
	Based on Median and with adjusted df	2.404	3	22	0.095
	Based on trimmed mean	4.466	3	37	0.009
Cu	Based on Mean	2.828	3	37	0.052
	Based on Median	2.846	3	37	0.051
	Based on Median and with adjusted df	2.846	3	32	0.053
	Based on trimmed mean	2.815	3	37	0.052
I	Based on Mean	3.927	3	37	0.016
	Based on Median	1.838	3	37	0.157
	Based on Median and with adjusted df	1.838	3	19	0.175
	Based on trimmed mean	3.202	3	37	0.034
Fe	Based on Mean	1.99	3	37	0.132
	Based on Median	0.788	3	37	0.508
	Based on Median and with adjusted df	0.788	3	14	0.521
	Based on trimmed mean	1.097	3	37	0.363
Mg	Based on Mean	5.675	3	37	0.003
	Based on Median	2.065	3	37	0.122
	Based on Median and with adjusted df	2.065	3	7	0.197
	Based on trimmed mean	5.223	3	37	0.004
Se	Based on Mean	5.771	3	37	0.002
	Based on Median	1.818	3	37	0.161
	Based on Median and with adjusted df	1.818	3	23	0.172
	Based on trimmed mean	5.01	3	37	0.005
Zn	Based on Mean	4.696	3	37	0.007
	Based on Median	0.846	3	37	0.477
	Based on Median and with adjusted df	0.846	3	11	0.497
	Based on trimmed mean	3.358	3	37	0.029
