# Supplementary material for: Variation in the mineral element concentration of Moringa oleifera Lam. and M. stenopetala (Bak. f.) Cuf.: Role in human nutrition
Source: PLoS One. 2017 Apr 7;12(4):e0175503. doi: 10.1371/journal.pone.0175503 (PMC5384779; doi:10.1371/journal.pone.0175503)
Supplement: S11 Table — (PDF) [file pone.0175503.s011.pdf]

S11 Table. Test of normality of the distribution of MO immature pods elemental concentration by locality.

Element	Locality	Shapiro-Wilk statistic	d.f.	*P*
Ca	**Kibwezi**	0.938	4	0.64
	**Mbololo**	0.933	8	0.548
	**Ramogi**	0.967	3	0.649
	**Ukunda**	0.887	3	0.345
Cu	**Kibwezi**	0.88	4	0.34
	**Mbololo**	0.909	8	0.349
	**Ramogi**	0.93	3	0.487
	**Ukunda**	0.951	3	0.573
Fe	**Kibwezi**	0.916	4	0.514
	**Mbololo**	0.9	8	0.288
	**Ramogi**	0.891	3	0.359
	**Ukunda**	0.777	3	0.062
Mg	**Kibwezi**	0.927	4	0.577
	**Mbololo**	0.956	8	0.773
	**Ramogi**	0.868	3	0.289
	**Ukunda**	0.998	3	0.914
Se	**Kibwezi**	0.743	4	0.033
	**Mbololo**	0.901	8	0.292
	**Ramogi**	0.985	3	0.765
	**Ukunda**	0.877	3	0.316
Zn	**Kibwezi**	0.982	4	0.913
	**Mbololo**	0.947	8	0.677
	**Ramogi**	0.988	3	0.789
	**Ukunda**	0.77	3	0.044
