# Supplementary material for: Variation in the mineral element concentration of Moringa oleifera Lam. and M. stenopetala (Bak. f.) Cuf.: Role in human nutrition
Source: PLoS One. 2017 Apr 7;12(4):e0175503. doi: 10.1371/journal.pone.0175503 (PMC5384779; doi:10.1371/journal.pone.0175503)
Supplement: S12 Table — (PDF) [file pone.0175503.s012.pdf]

S12 Table. Levene's test of homogeneity of variances of MO immature pods elemental concentration by localities.

Element	Levene statistic	d.f. 1	d.f. 2	*P*
**Ca**	1.531	4	15	0.244
**Cu**	1.704	4	15	0.201
**Fe**	7.541	4	15	0.002
**Mg**	4.463	4	15	0.014
**Se**	1.318	4	15	0.308
**Zn**	0.448	4	15	0.772
