# Supplementary material for: Variation in the mineral element concentration of Moringa oleifera Lam. and M. stenopetala (Bak. f.) Cuf.: Role in human nutrition
Source: PLoS One. 2017 Apr 7;12(4):e0175503. doi: 10.1371/journal.pone.0175503 (PMC5384779; doi:10.1371/journal.pone.0175503)
Supplement: S13 Table — (PDF) [file pone.0175503.s013.pdf]

S13 Table. Test of normality of the distribution of MO seeds elemental concentration by locality.

Element	Locality	Shapiro-Wilk statistic	d.f.	*p*
**Ca**	**Kibwezi**	0.909	9	0.186
	**Mbololo**	0.885	16	0.796
	**Ramogi**	0.752	7	0.805
**Cu**	**Kibwezi**	0.953	9	0.864
	**Mbololo**	0.955	16	0.744
	**Ramogi**	0.849	7	0.456
**Fe**	**Kibwezi**	0.914	9	0.752
	**Mbololo**	0.917	16	0.791
	**Ramogi**	0.958	7	0.400
**Mg**	**Kibwezi**	0.915	9	0.360
	**Mbololo**	0.912	16	0.961
	**Ramogi**	0.808	7	0.286
**Se**	**Kibwezi**	0.897	9	0.232
	**Mbololo**	0.842	16	0.007
	**Ramogi**	0.821	7	0.014
**Zn**	**Kibwezi**	0.903	9	0.288
	**Mbololo**	0.922	16	0.667
	**Ramogi**	0.942	7	0.972
