# Supplementary material for: Variation in the mineral element concentration of Moringa oleifera Lam. and M. stenopetala (Bak. f.) Cuf.: Role in human nutrition
Source: PLoS One. 2017 Apr 7;12(4):e0175503. doi: 10.1371/journal.pone.0175503 (PMC5384779; doi:10.1371/journal.pone.0175503)
Supplement: S14 Table — (PDF) [file pone.0175503.s014.pdf]

S14 Table. Levene's test of homogeneity of variances of MO seeds elemental concentration by localities.

Element	Levene statistic	d.f. 1	d.f. 2	*p*
**Ca**	0.721	2	29	0.495
**Cu**	2.598	2	29	0.092
**Fe**	0.223	2	29	0.801
**Mg**	1.630	2	29	0.213
**Se**	1.350	2	29	0.275
**Zn**	0.504	2	29	0.609
