# Supplementary material for: Variation in the mineral element concentration of Moringa oleifera Lam. and M. stenopetala (Bak. f.) Cuf.: Role in human nutrition
Source: PLoS One. 2017 Apr 7;12(4):e0175503. doi: 10.1371/journal.pone.0175503 (PMC5384779; doi:10.1371/journal.pone.0175503)
Supplement: S15 Table — (PDF) [file pone.0175503.s015.pdf]

S15 Table. Test of normality of the distribution of MO flowers elemental concentration by locality.

Element	Locality	Shapiro-Wilk statistic	d.f.	*P*
Ca	Malindi	0.956	7	0.787
	Mbololo	0.884	16	0.045
	Ramogi	0.941	7	0.646
	Ukunda	0.755	3	0.011
Cu	Malindi	0.963	7	0.843
	Mbololo	0.965	16	0.747
	Ramogi	0.936	7	0.601
	Ukunda	0.941	3	0.532
I	Malindi	0.478	7	0
	Mbololo	0.357	16	0
	Ramogi	0.857	7	0.143
	Ukunda	0.905	3	0.4
Fe	Malindi	0.788	7	0.031
	Mbololo	0.916	16	0.146
	Ramogi	0.921	7	0.477
	Ukunda	0.947	3	0.558
Mg	Malindi	0.942	7	0.655
	Mbololo	0.915	16	0.142
	Ramogi	0.959	7	0.809
	Ukunda	0.887	3	0.344
Se	Malindi	0.886	7	0.256
	Mbololo	0.742	16	0.001
	Ramogi	0.861	7	0.153
	Ukunda	0.879	3	0.321
Zn	Malindi	0.974	7	0.924
	Mbololo	0.903	16	0.091
	Ramogi	0.947	7	0.705
	Ukunda	0.991	3	0.822
