# Supplementary material for: Variation in the mineral element concentration of Moringa oleifera Lam. and M. stenopetala (Bak. f.) Cuf.: Role in human nutrition
Source: PLoS One. 2017 Apr 7;12(4):e0175503. doi: 10.1371/journal.pone.0175503 (PMC5384779; doi:10.1371/journal.pone.0175503)
Supplement: S16 Table — (PDF) [file pone.0175503.s016.pdf]

**S16 Table. Levene's test of homogeneity of variances of MO flowers elemental concentration by localities.**

| **Element** | **Levene statistic** | **d.f. 1** | **d.f. 2** | ***P*** |
|---|---|---|---|---|
| **Ca** | 2.282 | 3 | 29 | 0.1 |
| **Cu** | 2.127 | 3 | 29 | 0.118 |
| **I** | 1.687 | 3 | 29 | 0.192 |
| **Fe** | 20.373 | 3 | 29 | 0 |
| **Mg** | 6.803 | 3 | 29 | 0.001 |
| **Se** | 2.506 | 3 | 29 | 0.079 |
| **Zn** | 0.283 | 3 | 29 | 0.837 |
