# Supplementary material for: Variation in the mineral element concentration of Moringa oleifera Lam. and M. stenopetala (Bak. f.) Cuf.: Role in human nutrition
Source: PLoS One. 2017 Apr 7;12(4):e0175503. doi: 10.1371/journal.pone.0175503 (PMC5384779; doi:10.1371/journal.pone.0175503)
Supplement: S17 Table — (PDF) [file pone.0175503.s017.pdf]

S17 Table. Descriptive statistics of MO leaves elemental concentration (mg kg^{-1}) by locality.

Locality		Element						
		Ca	Cu	I	Fe	Mg	Se	Zn
Kibwezi	N	14	14	14	14	14	14	14
	Mean	14,960.160	9.179	0.152	193.383	5,400.566	5.742	45.791
	Median	12,562.529	8.873	0.152	176.566	5,316.767	4.529	46.204
	Std. Deviation	7,279.706	2.215	0.072	67.845	1,226.437	3.610	13.667
	Std. Error of Mean	1,945.583	0.592	0.019	18.132	327.779	0.965	3.653
	Minimum	7,037.418	5.902	0.069	114.234	3,509.752	1.937	26.908
	Maximum	30,580.651	13.900	0.308	325.598	7,716.198	12.502	68.153
Mbololo	N	16	16	16	16	16	16	16
	Mean	22,873.437	5.479	0.338	167.725	6,552.916	5.819	33.451
	Median	21,665.554	5.418	0.358	153.490	6,356.211	3.968	31.424
	Std. Deviation	7,280.129	1.221	0.100	56.486	1,513.341	5.941	10.356
	Std. Error of Mean	1,820.032	0.305	0.025	14.122	378.335	1.485	2.589
	Minimum	13,638.531	3.777	0.143	106.918	4,432.313	0.569	18.099
	Maximum	34,850.861	8.226	0.545	312.735	10,823.719	21.210	52.116
Ramogi	N	8	8	8	8	8	8	8
	Mean	16,316.006	6.912	0.067	402.855	4,915.632	0.839	29.592
	Median	11,908.853	7.125	0.037	310.538	5,090.188	0.620	26.224
	Std. Deviation	7,401.103	1.176	0.067	321.066	1,492.768	0.927	12.740
	Std. Error of Mean	2,616.685	0.416	0.024	113.514	527.773	0.328	4.504
	Minimum	8,551.457	5.161	0.001	193.676	2,524.808	0.031	14.399
	Maximum	27,546.755	8.590	0.204	1,171.058	7,485.904	2.851	54.391
Malindi	N	11	11	11	11	11	11	11
	Mean	17,199.049	7.268	0.247	129.044	4,470.598	3.215	34.392
	Median	15,946.089	7.559	0.254	125.327	4,750.177	2.576	33.722
	Std. Deviation	4,661.604	1.932	0.064	45.294	1,210.021	3.272	5.344
	Std. Error of Mean	1,405.526	0.583	0.019	13.657	364.835	0.987	1.611
	Minimum	10,924.482	3.005	0.135	74.934	3,094.520	0.774	25.053
	Maximum	23,153.497	10.424	0.366	227.622	5,958.689	12.537	42.851

Locality		Element						
		Ca	Cu	I	Fe	Mg	Se	Zn
Ukunda	N	7	7	7	7	7	7	7
	Mean	18,734.135	5.180	0.203	182.456	4,496.256	3.163	28.940
	Median	13,579.370	5.758	0.132	120.809	4,183.929	2.160	29.829
	Std. Deviation	13,045.504	1.391	0.160	109.738	1,292.254	3.093	6.622
	Std. Error of Mean	4,930.737	0.526	0.060	41.477	488.426	1.169	2.503
	Minimum	9,620.751	3.299	0.056	71.183	3,193.185	0.944	17.486
	Maximum	46,591.232	6.786	0.476	354.907	7,020.716	9.720	38.228
Total	N	56	56	56	56	56	56	56
	Mean	18,326.317	6.923	0.218	201.973	5,364.822	4.245	35.606
	Median	16,680.849	6.830	0.201	158.824	5,395.087	2.725	33.272
	Std. Deviation	8,138.245	2.223	0.130	155.263	1,551.893	4.376	11.958
	Std. Error of Mean	1,087.519	0.297	0.017	20.748	207.380	0.585	1.598
	Minimum	7,037.418	3.005	0.001	71.183	2,524.808	0.031	14.399
	Maximum	46,591.232	13.900	0.545	1,171.058	10,823.719	21.210	68.153
