# Supplementary material for: Variation in the mineral element concentration of Moringa oleifera Lam. and M. stenopetala (Bak. f.) Cuf.: Role in human nutrition
Source: PLoS One. 2017 Apr 7;12(4):e0175503. doi: 10.1371/journal.pone.0175503 (PMC5384779; doi:10.1371/journal.pone.0175503)
Supplement: S18 Table — (PDF) [file pone.0175503.s018.pdf]

S18 Table. Descriptive statistics for MS leaves elemental concentration (mg kg^{-1}) by locality.

Locality		Element						
		Ca	Cu	I	Fe	Mg	Se	Zn
Derashe	N	8	8	9	8	8	8	8
	Mean	22,089.115	5.156	0.029	125.665	7,991.397	1.371	24.644
	Median	21,554.019	5.342	0.029	120.825	7,969.240	0.260	18.599
	Std. Deviation	6,586.317	0.612	0.019	23.300	1,551.189	2.136	14.139
	Std. Error of Mean	2,328.615	0.216	0.006	8.238	548.428	0.755	4.999
	Minimum	14,522.685	4.282	0.001	98.536	5,806.893	0.080	15.424
	Maximum	32,888.314	5.984	0.063	164.959	10,197.398	6.175	56.923
Hawassa	N	14	14	14	14	14	14	14
	Mean	20,047.473	4.448	0.051	132.050	4,042.961	1.392	26.497
	Median	15,000.700	4.314	0.049	116.357	3,970.442	1.339	24.578
	Std. Deviation	11,523.479	1.180	0.033	43.608	1,030.168	0.412	5.318
	Std. Error of Mean	3,079.779	0.315	0.009	11.655	275.324	0.110	1.421
	Minimum	9,037.683	2.445	0.004	77.680	2,330.675	0.633	20.081
	Maximum	43,581.690	6.839	0.109	244.233	6,037.686	2.036	39.516
Konso	N	14	14	10	14	14	14	14
	Mean	20,594.913	4.759	0.050	196.409	6,953.243	1.329	18.759
	Median	19,943.419	4.721	0.031	109.520	6,699.051	0.240	18.115
	Std. Deviation	3,956.855	1.625	0.064	283.696	1,241.152	1.766	3.987
	Std. Error of Mean	1,057.514	0.434	0.020	75.821	331.712	0.472	1.066
	Minimum	13,727.290	1.290	0.001	72.034	5,415.358	0.036	10.532
	Maximum	28,905.411	6.854	0.221	1,165.833	9,147.332	4.643	26.173
Baringo	N	5	5	5	5	5	5	5
	Mean	23,544.784	3.154	0.237	207.359	9,198.380	3.783	16.135
	Median	22,494.501	3.067	0.243	189.925	7,206.049	3.962	15.696
	Std. Deviation	10,822.890	0.959	0.058	76.183	4,145.333	2.541	3.967
	Std. Error of Mean	4,840.144	0.429	0.026	34.070	1,853.849	1.136	1.774
	Minimum	10,058.043	1.759	0.169	143.434	6,069.678	1.098	11.497
	Maximum	40,319.734	4.418	0.312	330.570	16,234.149	7.396	22.001
	N	41	41	38	41	41	41	41

Locality		Element						
		Ca	Cu	I	Fe	Mg	Se	Zn
Total	Mean	21,059.274	4.534	0.070	161.965	6,435.852	1.658	22.229
	Median	20,045.246	4.464	0.038	122.286	6,178.372	1.208	20.383
	Std. Deviation	8,304.721	1.343	0.079	169.411	2,553.561	1.777	8.204
	Std. Error of Mean	1,296.980	0.210	0.013	26.458	398.799	0.277	1.281
	Minimum	9,037.683	1.290	0.001	72.034	2,330.675	0.036	10.532
	Maximum	43,581.690	6.854	0.312	1,165.833	16,234.149	7.396	56.923
Ethiopia	N	36	36	36	36	36	36	36
	Mean	20,714.064	4.726	0.191	155.660	6,052.167	1.363	23.076
	Median	19,353.013	4.705	0.093	116.937	6,064.526	1.118	20.946
	Std. Deviation	8,026.772	1.283	0.221	178.330	2,060.750	1.461	8.312
	Std. Error of Mean	1,337.795	0.214	0.037	29.722	343.458	0.244	1.385
	Minimum	9,037.683	1.290	0.004	72.034	2,330.675	0.036	10.532
	Maximum	43,581.690	6.854	0.871	1,165.833	10,197.398	6.175	56.923
Kenya	N	5	5	5	5	5	5	5
	Mean	23,544.784	3.154	0.210	207.359	9,198.380	3.783	16.135
	Median	22,494.501	3.067	0.231	189.925	7,206.049	3.962	15.696
	Std. Deviation	10,822.890	0.959	0.058	76.183	4,145.333	2.541	3.967
	Std. Error of Mean	4,840.144	0.429	0.026	34.070	1,853.849	1.136	1.774
	Minimum	10,058.043	1.759	0.146	143.434	6,069.678	1.098	11.497
	Maximum	40,319.734	4.418	0.268	330.570	16,234.149	7.396	22.001
