# Supplementary material for: Variation in the mineral element concentration of Moringa oleifera Lam. and M. stenopetala (Bak. f.) Cuf.: Role in human nutrition
Source: PLoS One. 2017 Apr 7;12(4):e0175503. doi: 10.1371/journal.pone.0175503 (PMC5384779; doi:10.1371/journal.pone.0175503)
Supplement: S19 Table — (PDF) [file pone.0175503.s019.pdf]

S19 Table. Descriptive statistics for MO immature pods elemental concentration (mg kg^{-1}) by locality.

Locality		Element					
		Ca	Cu	Fe	Mg	Se	Zn
Kibwezi	N	4	4	4	4	4	4
	Mean	3,856.707	7.668	79.559	3,252.703	4.299	32.598
	Median	4,102.595	7.793	81.074	3,137.504	3.422	32.690
	Std. Deviation	1,428.533	0.725	6.069	745.385	2.058	3.451
	Std. Error of Mea	714.266	0.362	3.034	372.693	1.029	1.725
	Minimum	1,901.944	6.792	71.075	2,470.055	2.991	28.306
	Maximum	5,319.696	8.293	85.015	4,265.749	7.360	36.708
Malindi	N	2	2	2	2	2	2
	Mean	2,168.956	3.943	57.668	1,829.595	2.216	23.703
	Median	2,168.956	3.943	57.668	1,829.595	2.216	23.703
	Std. Deviation	634.132	0.630	2.427	75.156	2.202	4.991
	Std. Error of Mea	448.399	0.445	1.717	53.144	1.557	3.529
	Minimum	1,720.558	3.497	55.952	1,776.451	0.659	20.174
	Maximum	2,617.355	4.388	59.385	1,882.738	3.773	27.232
Mbololo	N	8	8	8	8	8	8
	Mean	4,893.752	5.170	55.007	3,470.189	2.337	25.164
	Median	5,238.603	5.002	48.886	3,586.754	1.985	25.832
	Std. Deviation	1,599.666	1.405	14.666	1,172.171	2.015	4.458
	Std. Error of Mea	565.567	0.497	5.185	414.425	0.712	1.576
	Minimum	2,746.143	3.477	36.496	1,730.162	0.337	18.369
	Maximum	7,050.451	7.132	77.817	5,169.376	5.978	31.031
Ramogi	N	3	3	3	3	3	3
	Mean	2,707.043	6.378	66.491	2,485.935	0.211	33.159
	Median	2,607.266	6.852	62.760	2,575.122	0.183	32.575
	Std. Deviation	472.942	1.549	9.805	212.458	0.200	4.587
	Std. Error of Mea	273.053	0.895	5.661	122.663	0.115	2.649
	Minimum	2,291.950	4.646	59.100	2,243.421	0.027	28.891

Locality		Element					
		**Ca**	**Cu**	**Fe**	**Mg**	**Se**	**Zn**
	**Maximum**	3,221.912	7.634	77.614	2,639.264	0.424	38.010
**Ukunda**	**N**	3	3	3	3	3	3
	**Mean**	1,626.182	3.094	78.576	1,759.059	2.079	24.308
	**Median**	1,223.586	2.754	99.364	1,747.287	1.369	20.965
	**Std. Deviation**	1,037.495	1.326	38.163	226.071	1.753	6.033
	**Std. Error of Mea**	598.998	0.766	22.033	130.522	1.012	3.483
	**Minimum**	850.323	1.971	34.532	1,539.103	0.792	20.686
	**Maximum**	2,804.636	4.558	101.831	1,990.785	4.076	31.273
**Total**	**N**	20	20	20	20	20	20
	**Mean**	3,595.721	5.417	65.442	2,858.325	2.360	27.575
	**Median**	3,055.087	5.048	62.626	2,607.193	1.985	27.769
	**Std. Deviation**	1,760.177	1.894	19.245	1,057.747	2.069	5.702
	**Std. Error of Mea**	393.588	0.424	4.303	236.519	0.463	1.275
	**Minimum**	850.323	1.971	34.532	1,539.103	0.027	18.369
	**Maximum**	7,050.451	8.293	101.831	5,169.376	7.360	38.010
