# Supplementary material for: Variation in the mineral element concentration of Moringa oleifera Lam. and M. stenopetala (Bak. f.) Cuf.: Role in human nutrition
Source: PLoS One. 2017 Apr 7;12(4):e0175503. doi: 10.1371/journal.pone.0175503 (PMC5384779; doi:10.1371/journal.pone.0175503)
Supplement: S20 Table — (PDF) [file pone.0175503.s020.pdf]

S20 Table. Descriptive statistics for MO seeds elemental concentration (mg kg^{-1}) by locality.

Locality		Element					
		Ca	Cu	Fe	Mg	Se	Zn
Kibwezi	N	9	9	9	9	9	9
	Mean	1,089.027	4.782	54.838	3,005.682	3.547	45.164
	Median	1,028.429	4.669	53.515	3,112.816	3.444	43.512
	Std. Deviation	273.608	0.975	7.729	401.120	1.893	5.843
	Std. Error of Mean	91.203	0.325	2.576	133.707	0.631	1.948
	Minimum	797.616	3.403	43.892	2,332.004	1.602	38.296
	Maximum	1,557.737	6.239	67.880	3,472.875	7.586	53.427
Mbololo	N	16	16	16	16	16	16
	Mean	1,427.806	4.304	49.006	3,067.977	3.593	47.296
	Median	1,366.648	4.211	49.100	3,095.010	3.300	46.115
	Std. Deviation	357.141	0.927	6.755	255.113	2.492	7.019
	Std. Error of Mean	89.285	0.232	1.689	63.778	0.623	1.755
	Minimum	843.446	2.742	34.905	2,561.397	0.761	35.745
	Maximum	2,110.624	5.833	59.945	3,583.821	11.162	62.222
Ramogi	N	7	7	7	7	7	7
	Mean	1,224.474	3.209	47.203	3,302.894	0.699	45.544
	Median	1,254.570	3.197	44.598	3,266.416	0.341	46.074
	Std. Deviation	282.957	0.440	5.745	427.431	0.871	5.115
	Std. Error of Mean	106.948	0.166	2.171	161.554	0.329	1.933
	Minimum	769.201	2.752	40.737	2,882.802	0.015	37.228
	Maximum	1,698.394	4.003	56.128	4,035.181	2.542	53.505
Total	N	32	32	32	32	32	32
	Mean	1,288.046	4.199	50.252	3,101.845	2.947	46.313
	Median	1,253.081	4.024	49.793	3,103.926	2.636	46.015
	Std. Deviation	345.002	1.012	7.273	347.532	2.353	6.223
	Std. Error of Mean	60.988	0.179	1.286	61.435	0.416	1.100
	Minimum	769.201	2.742	34.905	2,332.004	0.015	35.745
	Maximum	2,110.624	6.239	67.880	4,035.181	11.162	62.222
