# Supplementary material for: Variation in the mineral element concentration of Moringa oleifera Lam. and M. stenopetala (Bak. f.) Cuf.: Role in human nutrition
Source: PLoS One. 2017 Apr 7;12(4):e0175503. doi: 10.1371/journal.pone.0175503 (PMC5384779; doi:10.1371/journal.pone.0175503)
Supplement: S21 Table — (PDF) [file pone.0175503.s021.pdf]

S21 Table. Descriptive statistics for MO flowers elemental concentration (mg kg^{-1}) by locality.

Locality		Element					
		Ca	Cu	Fe	Mg	Se	Zn
Malindi	N	7	7	7	7	7	7
	Mean	2,999.801	6.573	115.719	2,499.195	4.415	34.002
	Median	3,106.231	6.102	75.749	2,514.860	3.017	33.544
	Std. Deviation	1,203.615	2.156	60.699	177.194	3.532	5.391
	Std. Error of Mean	454.924	0.815	22.942	66.973	1.335	2.037
	Minimum	1,467.317	3.811	60.936	2,214.470	0.448	24.828
	Maximum	5,089.660	10.166	195.986	2,693.418	9.086	41.082
Mbololo	N	16	16	16	16	16	16
	Mean	4,436.461	6.333	219.815	3,177.500	3.027	31.311
	Median	4,579.819	6.252	198.502	3,256.363	1.938	29.478
	Std. Deviation	1,275.506	1.681	92.249	648.829	3.231	6.801
	Std. Error of Mean	318.876	0.420	23.062	162.207	0.808	1.700
	Minimum	2,674.313	3.464	105.882	2,338.926	0.570	20.798
	Maximum	6,160.185	9.066	384.560	4,219.717	12.997	47.248
Ramogi	N	7	7	7	7	7	7
	Mean	3,316.487	7.323	259.851	2,615.373	0.668	35.219
	Median	3,519.246	7.390	223.272	2,512.281	0.336	35.153
	Std. Deviation	904.368	1.100	88.747	602.025	0.702	8.069
	Std. Error of Mean	341.819	0.416	33.543	227.544	0.265	3.050
	Minimum	2,017.286	5.296	164.522	1,895.676	0.030	22.652
	Maximum	4,590.331	8.603	416.965	3,540.519	1.805	45.042
Ukunda	N	3	3	3	3	3	3
	Mean	1,788.378	4.177	731.886	2,264.732	2.930	30.605
	Median	1,607.169	4.306	891.968	2,350.993	1.768	31.181
	Std. Deviation	317.121	0.461	604.931	221.914	2.890	5.362
	Std. Error of Mean	183.090	0.266	349.257	128.122	1.669	3.096
	Minimum	1,603.414	3.666	63.014	2,012.640	0.801	24.978
	Maximum	2,154.551	4.560	1,240.676	2,430.564	6.220	35.656
	N	33	33	33	33	33	33

Locality		Element					
		Ca	Cu	Fe	Mg	Se	Zn
Total	Mean	3,653.410	6.398	252.778	2,831.399	2.812	32.647
	Median	3,415.872	6.253	194.733	2,613.325	1.561	31.676
	Std. Deviation	1,394.577	1.759	234.971	631.349	3.073	6.654
	Std. Error of Mean	242.765	0.306	40.903	109.904	0.535	1.158
	Minimum	1,467.317	3.464	60.936	1,895.676	0.030	20.798
	Maximum	6,160.185	10.166	1,240.676	4,219.717	12.997	47.248
