# Supplementary material for: Variation in the mineral element concentration of Moringa oleifera Lam. and M. stenopetala (Bak. f.) Cuf.: Role in human nutrition
Source: PLoS One. 2017 Apr 7;12(4):e0175503. doi: 10.1371/journal.pone.0175503 (PMC5384779; doi:10.1371/journal.pone.0175503)
Supplement: S22 Table — (PDF) [file pone.0175503.s022.pdf]

S22 Table. Descriptive statistics for soil elemental concentration (mg kg^{-1}) and pH by locality.

Locality		Element								
		Ca	Cu	I	Fe	Mg	Se	Se-P	Zn	pH
Baringo	N	5	5	5	5	5	5	5	5	5
	Mean	38,433.165	23.743	0.923	66,221.928	15,025.219	0.550	0.027	97.729	7.880
	Median	51,983.153	31.062	1.062	67,019.863	19,500.581	0.664	0.035	92.914	7.960
	Std. Deviation	25,302.405	12.264	0.709	11,369.686	10,132.554	0.291	0.016	15.465	0.193
	Std. Error of Mean	11,315.580	5.485	0.317	5,084.678	4,531.416	0.130	0.007	6.916	0.086
	Minimum	9,594.741	9.478	0.091	53,803.144	3,780.249	0.218	0.008	83.502	7.600
	Maximum	59,681.313	34.610	1.709	79,060.505	23,780.005	0.843	0.045	119.749	8.080
Derashe	N	12	12	12	12	12	12	12	12	12
	Mean	28,403.214	33.383	0.851	69,188.339	13,662.486	0.255	0.006	87.945	8.402
	Median	30,610.044	31.206	0.748	74,910.278	14,821.577	0.222	0.006	90.673	8.480
	Std. Deviation	9,945.190	5.987	0.607	22,443.902	5,213.568	0.083	0.004	23.943	0.281
	Std. Error of Mean	2,870.929	1.728	0.175	6,478.996	1,505.027	0.024	0.001	6.912	0.081
	Minimum	3,748.715	22.956	0.196	27,817.561	1,399.504	0.152	0.001	41.849	7.640
	Maximum	39,399.055	45.387	2.332	101,233.506	19,021.016	0.433	0.014	121.585	8.670
Hawassa	N	9	9	9	9	9	9	9	9	9
	Mean	15,752.996	12.951	1.131	35,393.976	4,796.370	0.599	0.011	259.637	7.426
	Median	14,126.779	8.624	0.941	32,902.160	3,798.195	0.622	0.010	228.647	7.660
	Std. Deviation	10,644.605	14.884	0.550	11,592.536	3,092.009	0.181	0.005	155.522	0.821
	Std. Error of Mean	3,548.202	4.961	0.183	3,864.179	1,030.670	0.060	0.002	51.841	0.274
	Minimum	4,682.805	4.373	0.646	23,331.785	1,780.680	0.363	0.005	121.331	6.120
	Maximum	39,050.078	52.372	2.049	61,432.348	11,775.353	0.799	0.019	627.064	8.380
Kibwezi	N	14	14	14	14	14	14	14	14	14
	Mean	14,159.658	23.967	1.718	33,018.946	4,020.536	0.362	0.024	53.998	7.879
	Median	12,800.030	21.693	1.406	29,296.002	3,255.077	0.366	0.023	51.488	7.890
	Std. Deviation	8,428.409	10.437	1.064	8,774.911	2,738.148	0.128	0.007	16.704	0.499
	Std. Error of Mean	2,252.587	2.789	0.284	2,345.193	731.801	0.034	0.002	4.464	0.133
	Minimum	4,803.902	11.103	0.557	18,399.321	1,003.312	0.194	0.016	35.085	6.970
	Maximum	36,880.760	40.265	3.672	47,632.026	11,591.483	0.655	0.039	88.822	8.590
	N	12	12	12	12	12	12	12	12	12

Locality		Element								
		Ca	Cu	I	Fe	Mg	Se	Se-P	Zn	pH
Konso	Mean	18,702.525	26.223	0.771	55,996.273	7,119.375	0.239	0.004	85.725	7.595
	Median	21,813.748	34.991	0.676	63,083.976	6,065.041	0.204	0.005	83.056	7.590
	Std. Deviation	8,615.910	17.638	0.379	31,041.357	4,842.516	0.097	0.002	44.051	0.582
	Std. Error of Mean	2,487.199	5.092	0.109	8,960.868	1,397.914	0.028	0.001	12.716	0.168
	Minimum	5,335.662	1.880	0.351	11,151.671	1,370.094	0.133	0.002	25.041	6.180
	Maximum	28,536.562	48.195	1.577	96,930.589	13,842.646	0.442	0.007	159.458	8.420
Malindi	N	11	11	11	11	11	11	11	11	11
	Mean	7,288.130	4.715	0.955	12,225.555	565.782	0.179	0.019	39.900	8.408
	Median	4,001.650	5.634	0.768	14,461.795	590.613	0.177	0.018	45.418	8.440
	Std. Deviation	8,384.839	2.121	0.371	6,324.562	272.922	0.044	0.007	19.006	0.217
	Std. Error of Mean	2,528.124	0.639	0.112	1,906.927	82.289	0.013	0.002	5.731	0.066
	Minimum	651.092	1.529	0.615	4,085.513	214.004	0.096	0.011	12.632	7.960
	Maximum	26,319.427	7.371	1.650	21,098.543	1,054.796	0.243	0.030	66.631	8.650
Mbololo	N	16	16	16	16	16	16	16	16	16
	Mean	13,393.396	10.247	1.434	22,315.490	3,211.001	0.241	0.020	39.843	7.531
	Median	12,898.731	9.375	1.298	21,298.290	3,256.232	0.203	0.018	39.681	7.495
	Std. Deviation	2,669.684	4.675	0.654	6,182.955	1,413.737	0.111	0.005	10.455	0.383
	Std. Error of Mean	667.421	1.169	0.164	1,545.739	353.434	0.028	0.001	2.614	0.096
	Minimum	8,158.021	3.766	0.648	13,104.357	979.712	0.127	0.014	23.871	6.630
	Maximum	18,612.468	21.015	2.773	37,236.338	5,787.440	0.467	0.030	60.408	8.100
Ramogi	N	8	8	8	8	8	8	8	8	8
	Mean	3,546.217	35.476	3.235	68,858.642	2,210.946	0.550	0.018	87.830	7.814
	Median	3,671.381	21.340	2.813	53,558.972	2,016.352	0.529	0.019	81.582	7.805
	Std. Deviation	1,440.642	25.722	2.019	40,116.229	1,237.976	0.109	0.004	28.454	0.306
	Std. Error of Mean	509.344	9.094	0.714	14,183.229	437.691	0.039	0.001	10.060	0.108
	Minimum	1,204.121	17.324	0.584	37,039.730	1,072.656	0.382	0.008	55.431	7.330
	Maximum	5,354.220	90.307	7.076	149,900.869	4,696.946	0.739	0.021	137.908	8.380
Ukunda	N	7	7	7	7	7	7	7	7	7
	Mean	12,849.236	7.551	1.001	7,687.053	715.256	0.155	0.014	92.343	7.937
	Median	14,636.954	7.073	0.923	8,035.247	839.060	0.169	0.013	102.307	7.830
	Std. Deviation	8,436.241	1.344	0.391	1,128.622	226.664	0.041	0.003	23.584	0.383

Locality		Element								
		Ca	Cu	I	Fe	Mg	Se	Se-P	Zn	pH
	**Std. Error of Mean**	3,188.599	0.508	0.148	426.579	85.671	0.015	0.001	8.914	0.145
	**Minimum**	2,407.880	6.112	0.373	5,504.539	370.677	0.113	0.009	54.502	7.600
	**Maximum**	25,606.797	9.219	1.455	9,118.297	887.839	0.220	0.017	115.146	8.780
**Ethiopia**	**N**	33	33	33	33	33	33	33	33	33
	**Mean**	21,425.631	25.207	0.898	55,174.580	8,865.141	0.343	0.007	133.963	7.842
	**Median**	22,288.739	30.729	0.773	51,189.400	6,704.319	0.281	0.006	100.715	7.980
	**Std. Deviation**	10,859.675	15.567	0.523	26,868.612	5,850.130	0.198	0.005	114.104	0.708
	**Std. Error of Mean**	1,890.427	2.710	0.091	4,677.225	1,018.377	0.034	0.001	19.863	0.123
	**Minimum**	3,748.715	1.880	0.196	11,151.671	1,370.094	0.133	0.001	25.041	6.120
	**Maximum**	39,399.055	52.372	2.332	101,233.506	19,021.016	0.799	0.019	627.064	8.670
**Kenya**	**N**	61	61	61	61	61	61	61	61	61
	**Mean**	13,166.872	16.504	1.558	30,976.760	3,470.616	0.313	0.020	60.165	7.881
	**Median**	11,783.116	11.324	1.195	23,466.670	2,297.570	0.239	0.018	53.457	7.850
	**Std. Deviation**	12,290.728	15.094	1.192	25,941.909	4,798.291	0.186	0.008	29.133	0.460
	**Std. Error of Mean**	1,573.666	1.933	0.153	3,321.521	614.358	0.024	0.001	3.730	0.059
	**Minimum**	651.092	1.529	0.091	4,085.513	214.004	0.096	0.008	12.632	6.630
	**Maximum**	59,681.313	90.307	7.076	149,900.869	23,780.005	0.843	0.045	137.908	8.780
**Total**	**N**	94	94	94	94	94	94	94	94	94
	**Mean**	16,066.223	19.559	1.326	39,471.739	5,364.439	0.324	0.016	86.072	7.867
	**Median**	13,741.433	12.950	0.972	29,692.203	3,130.932	0.240	0.015	65.989	7.875
	**Std. Deviation**	12,399.362	15.742	1.054	28,590.477	5,773.295	0.190	0.009	79.256	0.556
	**Std. Error of Mean**	1,278.897	1.624	0.109	2,948.883	595.470	0.020	0.001	8.175	0.057
	**Minimum**	651.092	1.529	0.091	4,085.513	214.004	0.096	0.001	12.632	6.120
	**Maximum**	59,681.313	90.307	7.076	149,900.869	23,780.005	0.843	0.045	627.064	8.780
