# Supplementary material for: Variation in the mineral element concentration of Moringa oleifera Lam. and M. stenopetala (Bak. f.) Cuf.: Role in human nutrition
Source: PLoS One. 2017 Apr 7;12(4):e0175503. doi: 10.1371/journal.pone.0175503 (PMC5384779; doi:10.1371/journal.pone.0175503)
Supplement: S23 Table — (PDF) [file pone.0175503.s023.pdf]

S23 Table. Spearman's rank correlation (N = 56, d.f. = 54) between the elemental concentration of MO leaves and soil properties.

**MO Leaves**	**Ca**	1															
	**Cu**	-0.383	1.000														
	**Fe**	0.101	-0.024	1.000													
	**I**	0.011	-0.124	-0.125	1.000												
	**Mg**	0.581	-0.158	0.121	-0.164	1.000											
	**Se**	0.183	-0.055	-0.253	-0.427	0.397	1.000										
	**Zn**	-0.422	0.463	-0.091	-0.195	0.001	0.227	1.000									
**Soil**	**Ca**	0.213	-0.052	-0.213	-0.080	0.078	0.235	0.103	1.000								
	**Cu**	-0.183	0.277	0.476	-0.286	0.049	-0.068	0.133	0.139	1.000							
	**Fe**	-0.077	0.281	0.498	-0.298	0.152	-0.087	0.114	0.047	0.910	1.000						
	**I**	-0.034	0.136	0.245	-0.137	0.046	-0.160	0.068	0.030	0.600	0.614	1.000					
	**Mg**	0.094	0.118	0.197	-0.324	0.284	0.232	0.148	0.539	0.702	0.721	0.442	1.000				
	**Se**	-0.127	0.216	0.464	-0.337	0.079	0.026	0.091	-0.189	0.790	0.814	0.579	0.499	1.000			
	**Se-P**	0.139	0.107	-0.022	-0.256	0.324	0.437	0.169	-0.026	0.345	0.367	0.301	0.427	0.594	1.000		
	**Zn**	-0.134	-0.001	0.306	-0.027	-0.171	-0.306	-0.061	0.083	0.457	0.328	0.273	0.045	0.330	-0.061	1.000	
	**pH**	-0.074	0.177	-0.308	0.039	-0.271	-0.005	-0.006	0.023	-0.255	-0.238	-0.234	-0.337	-0.065	-0.016	0.132	1.000
		**Ca**	**Cu**	**Fe**	**I**	**Mg**	**Se**	**Zn**	**Ca**	**Cu**	**Fe**	**I**	**Mg**	**Se**	**Se-P**	**Zn**	**pH**
		**MO Leaves**							**Soil**								
