# Supplementary material for: Variation in the mineral element concentration of Moringa oleifera Lam. and M. stenopetala (Bak. f.) Cuf.: Role in human nutrition
Source: PLoS One. 2017 Apr 7;12(4):e0175503. doi: 10.1371/journal.pone.0175503 (PMC5384779; doi:10.1371/journal.pone.0175503)
Supplement: S24 Table — Significant correlations are in bold. (PDF) [file pone.0175503.s024.pdf]

S24 Table. The *t* probabilities for the Spearman's rank correlation between the elemental concentration of MO leaves and soil properties.

**MO Leaves**	**Ca**																
	**Cu**	**0.004**															
	**Fe**	0.457	0.862														
	**I**	0.935	0.364	0.358													
	**Mg**	**0.000**	0.244	0.373	0.228												
	**Se**	0.178	0.688	0.060	**0.001**	**0.002**											
	**Zn**	**0.001**	**0.000**	0.506	0.149	0.993	0.092										
**Soil**	**Ca**	0.116	0.702	0.114	0.557	0.568	0.081	0.452									
	**Cu**	0.178	**0.039**	**0.000**	**0.033**	0.722	0.616	0.327	0.307								
	**Fe**	0.572	**0.036**	**0.000**	**0.026**	0.264	0.526	0.402	0.732	**0.000**							
	**I**	0.802	0.318	0.069	0.316	0.735	0.240	0.618	0.824	**0.000**	**0.000**						
	**Mg**	0.490	0.387	0.146	0.015	0.034	0.085	0.278	0.000	**0.000**	**0.000**	**0.001**					
	**Se**	0.351	0.110	0.000	0.011	0.562	0.847	0.503	0.162	**0.000**	**0.000**	**0.000**	**0.000**				
	**Se-P**	0.306	0.434	0.872	0.057	**0.015**	**0.001**	0.212	0.850	**0.009**	**0.005**	**0.024**	**0.001**	0.000			
	**Zn**	0.324	0.993	**0.022**	0.846	0.206	**0.022**	0.654	0.541	**0.000**	**0.014**	**0.042**	0.742	**0.013**	0.657		
	**pH**	0.589	0.191	0.021	0.774	0.044	0.973	0.964	0.869	0.057	0.078	0.083	**0.011**	0.633	0.906	0.330	
		**Ca**	**Cu**	**Fe**	**I**	**Mg**	**Se**	**Zn**	**Ca**	**Cu**	**Fe**	**I**	**Mg**	**Se**	**Se-P**	**Zn**	**pH**
		**MO Leaves**							**Soil**								
