# Supplementary material for: Variation in the mineral element concentration of Moringa oleifera Lam. and M. stenopetala (Bak. f.) Cuf.: Role in human nutrition
Source: PLoS One. 2017 Apr 7;12(4):e0175503. doi: 10.1371/journal.pone.0175503 (PMC5384779; doi:10.1371/journal.pone.0175503)
Supplement: S25 Table — (PDF) [file pone.0175503.s025.pdf]

S25 Table. Spearman's rank correlation (N = 32, d.f. = 30) between the elemental concentration of MS leaves and soil properties.

MS Leaves	**Ca**	1															
	**Cu**	0.240	1.000														
	**Fe**	0.153	-0.080	1.000													
	**I**	-0.117	-0.351	0.367	1.000												
	**Mg**	0.788	0.057	0.069	-0.073	1.000											
	**Se**	0.048	-0.221	0.340	0.235	0.025	1.000										
	**Zn**	-0.259	0.289	-0.128	-0.164	-0.458	0.219	1.000									
Soil	**Ca**	0.195	0.023	0.342	-0.123	0.267	-0.009	-0.051	1.000								
	**Cu**	0.142	0.280	0.212	-0.132	0.112	-0.304	-0.120	0.717	1.000							
	**Fe**	0.347	0.151	0.288	0.058	0.224	-0.100	-0.142	0.677	0.730	1.000						
	**I**	0.127	0.176	0.079	0.014	-0.165	0.135	0.243	-0.113	-0.001	0.187	1.000					
	**Mg**	0.348	0.133	0.410	-0.005	0.327	-0.062	-0.154	0.856	0.679	0.840	0.067	1.000				
	**Se**	-0.173	-0.129	0.441	0.240	-0.484	0.422	0.245	0.005	0.003	0.126	0.620	0.085	1.000			
	**Se-P**	-0.194	-0.239	0.444	0.283	-0.218	0.609	-0.009	-0.135	-0.214	-0.156	0.486	-0.103	0.712	1.000		
	**Zn**	-0.306	0.025	0.251	0.176	-0.687	0.147	0.442	0.046	0.093	0.200	0.443	0.087	0.803	0.274	1.000	
	**pH**	0.214	0.270	0.083	-0.333	0.297	-0.019	0.231	0.637	0.494	0.410	-0.039	0.500	-0.139	-0.204	-0.135	1.000
		**Ca**	**Cu**	**Fe**	**I**	**Mg**	**Se**	**Zn**	**Ca**	**Cu**	**Fe**	**I**	**Mg**	**Se**	**Se-P**	**Zn**	**pH**
		**MS Leaves**							**Soil**								
