# Supplementary material for: Variation in the mineral element concentration of Moringa oleifera Lam. and M. stenopetala (Bak. f.) Cuf.: Role in human nutrition
Source: PLoS One. 2017 Apr 7;12(4):e0175503. doi: 10.1371/journal.pone.0175503 (PMC5384779; doi:10.1371/journal.pone.0175503)
Supplement: S26 Table — Significant correlations are in bold. (PDF) [file pone.0175503.s026.pdf]

**S26 Table. The *t* probabilities for the Spearman's rank correlation between the elemental concentration of MS leaves and soil properties. Significant correlations are in bold.**

| | | | | | | | | | | | | | | | | | |
|---|---|---|---|---|---|---|---|---|---|---|---|---|---|---|---|---|---|
| | **Ca** | | | | | | | | | | | | | | | | |
| | **Cu** | 0.186 | | | | | | | | | | | | | | | |
| | **Fe** | 0.402 | 0.665 | | | | | | | | | | | | | | |
| **MS Leaves** | **I** | 0.523 | 0.049 | **0.039** | | | | | | | | | | | | | |
| | **Mg** | **0.000** | 0.757 | 0.709 | 0.690 | | | | | | | | | | | | |
| | **Se** | 0.793 | 0.223 | 0.057 | 0.196 | 0.894 | | | | | | | | | | | |
| | **Zn** | 0.152 | 0.108 | 0.484 | 0.370 | 0.008 | 0.229 | | | | | | | | | | |
| | **Ca** | 0.284 | 0.899 | 0.055 | 0.503 | 0.139 | 0.962 | 0.782 | | | | | | | | | |
| | **Cu** | 0.439 | 0.120 | 0.244 | 0.472 | 0.542 | 0.090 | 0.512 | **0.000** | | | | | | | | |
| | **Fe** | 0.052 | 0.409 | 0.110 | 0.754 | 0.219 | 0.584 | 0.439 | **0.000** | **0.000** | | | | | | | |
| | **I** | 0.488 | 0.336 | 0.668 | 0.938 | 0.368 | 0.462 | 0.181 | 0.538 | 0.995 | 0.307 | | | | | | |
| **Soil** | **Mg** | 0.051 | 0.469 | **0.020** | 0.979 | 0.068 | 0.738 | 0.400 | **0.000** | **0.000** | **0.000** | 0.717 | | | | | |
| | **Se** | 0.343 | 0.482 | **0.012** | 0.185 | 0.005 | 0.016 | 0.177 | 0.976 | 0.986 | 0.490 | **0.000** | 0.644 | | | | |
| | **Se-P** | 0.288 | 0.188 | **0.011** | 0.117 | 0.230 | **0.000** | 0.962 | 0.460 | 0.239 | 0.393 | **0.005** | 0.576 | **0.000** | | | |
| | **Zn** | 0.088 | 0.892 | 0.166 | 0.336 | **0.000** | 0.423 | **0.011** | 0.802 | 0.612 | 0.273 | **0.011** | 0.635 | **0.000** | 0.129 | | |
| | **pH** | 0.239 | 0.134 | 0.653 | 0.063 | 0.099 | 0.920 | 0.203 | **0.000** | **0.004** | **0.020** | 0.832 | **0.004** | 0.448 | 0.264 | 0.462 | |
| | | **Ca** | **Cu** | **Fe** | **I** | **Mg** | **Se** | **Zn** | **Ca** | **Cu** | **Fe** | **I** | **Mg** | **Se** | **Se-P** | **Zn** | **pH** |
| | | **MS leaves** | | | | | | | **Soil** | | | | | | | | |
