# Supplementary material for: Variation in the mineral element concentration of Moringa oleifera Lam. and M. stenopetala (Bak. f.) Cuf.: Role in human nutrition
Source: PLoS One. 2017 Apr 7;12(4):e0175503. doi: 10.1371/journal.pone.0175503 (PMC5384779; doi:10.1371/journal.pone.0175503)
Supplement: S27 Table — d.f. 1 (degrees of freedom of the numerator), d.f. 2 (degrees of freedom of the denominator), and the p probability value. (PDF) [file pone.0175503.s027.pdf]

S27 Table. Welch's robust test of equality of mean elemental concentrations in MO leaves across localities in Kenya, d.f. 1 (degrees of freedom of the numerator), d.f. 2 (degrees of freedom of the denominator), and the *P* value.

Element	Welch statistic	d.f. 1	d.f. 2	*P*
Ca	2.362	4	21	0.087
Cu	9.203	4	22	0.000
I	3.682	4	17	0.024
Fe	3.107	4	20	0.039
Mg	4.438	4	21	0.009
Zn	3.561	4	22	0.022
Se	8.246	4	22	0.000
