# Supplementary material for: Variation in the mineral element concentration of Moringa oleifera Lam. and M. stenopetala (Bak. f.) Cuf.: Role in human nutrition
Source: PLoS One. 2017 Apr 7;12(4):e0175503. doi: 10.1371/journal.pone.0175503 (PMC5384779; doi:10.1371/journal.pone.0175503)
Supplement: S28 Table — d.f. 1 (degrees of freedom of the numerator), d.f. 2 (degrees of freedom of the denominator), and the p (probability value). (PDF) [file pone.0175503.s028.pdf]

S28 Table. Welch's robust test of equality of mean elemental concentrations in MS leaves across localities. Refer to Supplementary Table 28 for abbreviations.

**Element**	**Welch statistic**	**d.f. 1**	**d.f. 2**	***p***
**Ca**	0.216	3	12	0.883
**Cu**	5.538	3	15	0.009
**I**	18.296	3	13	0.000
**Fe**	1.883	3	14	0.180
**Mg**	21.481	3	12	0.000
**Se**	1.315	3	11	0.319
**Zn**	8.266	3	14	0.002
