# Supplementary material for: Variation in the mineral element concentration of Moringa oleifera Lam. and M. stenopetala (Bak. f.) Cuf.: Role in human nutrition
Source: PLoS One. 2017 Apr 7;12(4):e0175503. doi: 10.1371/journal.pone.0175503 (PMC5384779; doi:10.1371/journal.pone.0175503)
Supplement: S29 Table — d.f. 1 (degrees of freedom of the numerator), d.f. 2 (degrees of freedom of the denominator), and the p (probability value). (PDF) [file pone.0175503.s029.pdf]

S29 Table. Welch's robust test of equality of mean elemental concentrations in MO and MS leaves. Refer to S Table 28 for abbreviations.

Element	Welch statistic	d.f. 1	d.f. 2	*p*
**Ca**	2.607	1	85	0.110
**Cu**	43.118	1	92	0.000
**I**	26.356	1	86	0.000
**Fe**	1.416	1	82	0.238
**Mg**	5.677	1	61	0.020
**Se**	15.975	1	77	0.000
**Zn**	42.651	1	95	0.000
