# Supplementary material for: Variation in the mineral element concentration of Moringa oleifera Lam. and M. stenopetala (Bak. f.) Cuf.: Role in human nutrition
Source: PLoS One. 2017 Apr 7;12(4):e0175503. doi: 10.1371/journal.pone.0175503 (PMC5384779; doi:10.1371/journal.pone.0175503)
Supplement: S30 Table — d.f. 1 (degrees of freedom of the numerator), d.f. 2 (degrees of freedom of the denominator), and the p (probability value). (PDF) [file pone.0175503.s030.pdf]

S30 Table. Welch's robust test of equality of mean elemental concentrations in MO immature pods across localities. Refer to S Table 28 for abbreviations.

Element	Welch statistic	d.f. 1	d.f. 2	*p*
**Ca**	3.91	4	5	0.082
**Cu**	10.37	4	5	0.013
**Fe**	7.91	4	6	0.016
**Mg**	9.81	4	6	0.008
**Se**	4.77	4	4	0.077
**Zn**	2.82	4	4	0.160
