# Supplementary material for: Variation in the mineral element concentration of Moringa oleifera Lam. and M. stenopetala (Bak. f.) Cuf.: Role in human nutrition
Source: PLoS One. 2017 Apr 7;12(4):e0175503. doi: 10.1371/journal.pone.0175503 (PMC5384779; doi:10.1371/journal.pone.0175503)
Supplement: S31 Table — d.f. 1 (degrees of freedom of the numerator), d.f. 2 (degrees of freedom of the denominator), and the p (probability value). (PDF) [file pone.0175503.s031.pdf]

S31 Table. Welch's robust test of equality of mean elemental concentrations in MO seeds across localities. Refer to S Table 28 for abbreviations.

Element	Welch statistic	d.f. 1	d.f. 2	*P*
**Ca**	4.441	2	6	0.061
**Cu**	2.767	2	8	0.119
**I**	1.448	2	9	0.288
**Fe**	1.426	2	5	0.321
**Mg**	0.256	2	5	0.785
**Se**	3.933	2	8	0.066
**Zn**	0.093	2	5	0.913
