# Supplementary material for: Variation in the mineral element concentration of Moringa oleifera Lam. and M. stenopetala (Bak. f.) Cuf.: Role in human nutrition
Source: PLoS One. 2017 Apr 7;12(4):e0175503. doi: 10.1371/journal.pone.0175503 (PMC5384779; doi:10.1371/journal.pone.0175503)
Supplement: S32 Table — d.f. 1 (degrees of freedom of the numerator), d.f. 2 (degrees of freedom of the denominator), and the p (probability value). (PDF) [file pone.0175503.s032.pdf]

S32 Table. Welch's robust test of equality of mean elemental concentrations in MO flowers across localities. Refer to S Table 28 for abbreviations.

**Element**	**Welch statistic**	**d.f. 1**	**d.f. 2**	***p***
**Ca**	17.47	3	13	0.000
**Cu**	14.88	3	12	0.000
**Fe**	5.3	3	7	0.031
**Mg**	5.96	3	9	0.016
**Se**	4.41	3	7	0.047
**Zn**	0.63	3	8	0.615
