# Supplementary material for: Variation in the mineral element concentration of Moringa oleifera Lam. and M. stenopetala (Bak. f.) Cuf.: Role in human nutrition
Source: PLoS One. 2017 Apr 7;12(4):e0175503. doi: 10.1371/journal.pone.0175503 (PMC5384779; doi:10.1371/journal.pone.0175503)
Supplement: S33 Table — d.f. 1 (degrees of freedom of the numerator), d.f. 2 (degrees of freedom of the denominator), and the p (probability value). (PDF) [file pone.0175503.s033.pdf]

S33 Table. Welch's robust tests of equality of mean soil elemental concentrations across localities. Refer to S Table 28 for abbreviations.

**Element**	**Welch's statistic**	**d.f.1**	**d.f.2**	***p***
Ca	24.956	8	29	0.000
Cu	33.272	8	30	0.000
Fe	51.82	8	29	0.000
Mg	21.974	8	29	0.000
Se	18.016	8	29	0.000
Zn	16.211	8	28	0.000
pH	10.983	8	30	0.000
I	3.126	8	29	0.011
Se_p	28.387	8	29	0.000
