# Supplementary material for: Variation in the mineral element concentration of Moringa oleifera Lam. and M. stenopetala (Bak. f.) Cuf.: Role in human nutrition
Source: PLoS One. 2017 Apr 7;12(4):e0175503. doi: 10.1371/journal.pone.0175503 (PMC5384779; doi:10.1371/journal.pone.0175503)
Supplement: S34 Table — (PDF) [file pone.0175503.s034.pdf]

S34 Table. Raw data on MO and MS edible parts elemental concentration (mg kg^{-1}), and sample details.

Sample_ID	Household_ID	Country	Locality	Edible part	Species	Ca	Cu	Fe	Mg	Se	Zn
ETF0001	KaratKonso	Ethiopia	Konso	Leaf	MS	26938.248	6.982	140.433	6976.179	3.807	20.574
ETF0001	KaratKonso	Ethiopia	Konso	Leaf	MS	26277.274	6.948	135.903	6803.033	3.697	20.421
ETF0001	KaratKonso	Ethiopia	Konso	Leaf	MS	25938.950	6.631	126.202	6715.551	3.586	20.153
ETF0002	KaratKonso	Ethiopia	Konso	Leaf	MS	23525.447	5.919	1168.674	7655.913	2.567	18.154
ETF0002	KaratKonso	Ethiopia	Konso	Leaf	MS	24217.198	6.043	1230.288	7828.621	2.559	18.145
ETF0002	KaratKonso	Ethiopia	Konso	Leaf	MS	22926.495	6.134	1098.538	7476.495	2.550	17.755
ETF0005	ETH001	Ethiopia	Derashe	Leaf	MS	33015.858	5.666	139.802	9755.571	0.211	17.716
ETF0005	ETH001	Ethiopia	Derashe	Leaf	MS	32923.972	5.524	136.317	9796.096	0.142	17.875
ETF0005	ETH001	Ethiopia	Derashe	Leaf	MS	32725.112	5.907	134.653	9660.750	0.126	17.494
ETF0009	ETH002	Ethiopia	Derashe	Leaf	MS	14388.280	4.351	117.435	5744.306	2.674	18.701
ETF0009	ETH002	Ethiopia	Derashe	Leaf	MS	14604.953	4.468	116.371	5854.149	2.654	18.814
ETF0009	ETH002	Ethiopia	Derashe	Leaf	MS	14574.821	4.384	113.404	5822.226	2.619	18.898
ETF0012	ETH003	Ethiopia	Derashe	Leaf	MS	20996.208	6.698	145.663	6904.307	0.642	23.427
ETF0012	ETH003	Ethiopia	Derashe	Leaf	MS	19778.907	6.299	130.865	6454.789	0.616	22.235
ETF0012	ETH003	Ethiopia	Derashe	Leaf	MS	20563.350	6.608	132.010	6683.433	0.602	22.744
ETF0013	ETH004	Ethiopia	Derashe	Leaf	MS	14295.638	7.297	232.877	7543.281	0.325	35.936
ETF0013	ETH004	Ethiopia	Derashe	Leaf	MS	14553.946	7.806	245.578	7609.558	0.315	36.836
ETF0013	ETH004	Ethiopia	Derashe	Leaf	MS	14457.197	7.484	244.264	7586.614	0.275	36.614
ETF0014	ETH005	Ethiopia	Derashe	Leaf	MS	15403.313	6.394	135.717	7551.240	0.262	27.691
ETF0014	ETH005	Ethiopia	Derashe	Leaf	MS	14790.810	6.362	129.935	7239.342	0.230	26.501
ETF0014	ETH005	Ethiopia	Derashe	Leaf	MS	14674.229	6.249	128.064	7145.434	0.224	26.079
ETF0015	ETH006	Ethiopia	Derashe	Leaf	MS	12341.907	7.895	141.429	6617.763	0.311	30.133
ETF0015	ETH006	Ethiopia	Derashe	Leaf	MS	12062.904	7.757	133.324	6416.182	0.308	29.210
ETF0015	ETH006	Ethiopia	Derashe	Leaf	MS	12595.631	7.987	137.261	6697.688	0.305	30.692
ETF0016	ETH007	Ethiopia	Derashe	Leaf	MS	29688.037	5.085	152.052	10349.706	1.316	56.842
ETF0016	ETH007	Ethiopia	Derashe	Leaf	MS	28715.176	4.646	148.347	10112.596	1.285	58.472
ETF0016	ETH007	Ethiopia	Derashe	Leaf	MS	28870.148	4.574	145.474	10129.894	1.258	55.455
ETF0017	ETH008	Ethiopia	Derashe	Leaf	MS	24611.694	5.432	100.731	8698.949	6.253	33.368

Sample_ID	Household_ID	Country	Locality	Edible part	Species	Ca	Cu	Fe	Mg	Se	Zn
ETF0017	ETH008	Ethiopia	Derashe	Leaf	MS	24983.254	5.540	99.105	8783.610	6.185	33.504
ETF0017	ETH008	Ethiopia	Derashe	Leaf	MS	24267.839	5.311	95.771	8573.695	6.086	32.558
ETF0019	ETH009	Ethiopia	Derashe	Leaf	MS	23209.691	5.689	166.804	8419.756	0.088	18.125
ETF0019	ETH009	Ethiopia	Derashe	Leaf	MS	23008.450	6.654	161.367	8269.719	0.077	18.126
ETF0019	ETH009	Ethiopia	Derashe	Leaf	MS	22970.236	5.608	166.705	8327.279	0.075	17.636
ETF0022	ETH010	Ethiopia	Derashe	Leaf	MS	15895.477	4.284	125.593	6401.204	0.321	15.533
ETF0022	ETH010	Ethiopia	Derashe	Leaf	MS	15297.163	4.207	119.547	6169.281	0.320	15.084
ETF0022	ETH010	Ethiopia	Derashe	Leaf	MS	15612.744	4.355	121.719	6310.494	0.263	15.656
ETF0025	ETH011	Ethiopia	Derashe	Leaf	MS	20526.978	5.413	100.177	7477.320	0.130	19.141
ETF0025	ETH011	Ethiopia	Derashe	Leaf	MS	19429.197	5.396	97.143	7026.095	0.089	18.299
ETF0025	ETH011	Ethiopia	Derashe	Leaf	MS	20179.562	5.470	99.366	7312.143	0.071	18.819
ETF0026	ETH012	Ethiopia	Derashe	Leaf	MS	16957.478	5.292	120.957	7626.004	0.229	18.601
ETF0026	ETH012	Ethiopia	Derashe	Leaf	MS	16680.040	5.182	119.571	7517.358	0.221	18.360
ETF0026	ETH012	Ethiopia	Derashe	Leaf	MS	17002.599	5.298	117.560	7655.323	0.205	18.373
ETF0032	ETH013	Ethiopia	Konso	Leaf	MS	21095.662	5.542	72.041	7622.067	0.088	18.653
ETF0032	ETH013	Ethiopia	Konso	Leaf	MS	20517.120	5.467	70.800	7460.254	0.067	18.780
ETF0032	ETH013	Ethiopia	Konso	Leaf	MS	20146.075	5.340	68.284	7330.805	0.059	18.150
ETF0033	ETH014	Ethiopia	Konso	Leaf	MS	18168.190	5.429	86.948	6054.982	0.342	19.679
ETF0033	ETH014	Ethiopia	Konso	Leaf	MS	17785.625	5.068	83.269	5853.825	0.324	18.832
ETF0033	ETH014	Ethiopia	Konso	Leaf	MS	17581.780	5.083	86.444	5839.487	0.320	18.896
ETF0035	ETH015	Ethiopia	Konso	Leaf	MS	16988.659	6.081	77.900	5359.819	0.069	25.857
ETF0035	ETH015	Ethiopia	Konso	Leaf	MS	17084.491	6.208	81.644	5415.891	0.065	26.169
ETF0035	ETH015	Ethiopia	Konso	Leaf	MS	17264.911	6.268	81.376	5470.365	0.056	26.492
ETF0036	ETH016	Ethiopia	Konso	Leaf	MS	20558.082	6.224	113.373	6967.601	0.171	18.633
ETF0036	ETH016	Ethiopia	Konso	Leaf	MS	20104.944	6.151	112.051	6855.388	0.167	18.137
ETF0036	ETH016	Ethiopia	Konso	Leaf	MS	19907.012	6.148	115.715	6776.043	0.155	17.869
ETF0039	ETH017	Ethiopia	Konso	Leaf	MS	19652.919	1.329	106.411	6558.895	0.277	17.513
ETF0039	ETH017	Ethiopia	Konso	Leaf	MS	19639.312	1.266	104.605	6548.758	0.260	17.392
ETF0039	ETH017	Ethiopia	Konso	Leaf	MS	19798.244	1.274	104.966	6591.889	0.250	17.287
ETF0042	ETH018	Ethiopia	Konso	Leaf	MS	21020.100	3.097	72.317	8607.909	0.507	21.125
ETF0042	ETH018	Ethiopia	Konso	Leaf	MS	20397.019	2.995	70.807	8332.670	0.506	21.485

Sample_ID	Household_ID	Country	Locality	Edible part	Species	Ca	Cu	Fe	Mg	Se	Zn
ETF0042	ETH018	Ethiopia	Konso	Leaf	MS	20775.942	3.067	72.977	8482.478	0.505	21.168
ETF0045	ETH019	Ethiopia	Konso	Leaf	MS	13711.701	3.484	85.553	7238.559	0.229	16.930
ETF0045	ETH019	Ethiopia	Konso	Leaf	MS	13655.293	3.483	86.015	7192.075	0.215	17.268
ETF0045	ETH019	Ethiopia	Konso	Leaf	MS	13814.874	3.496	88.061	7255.494	0.189	16.756
ETF0048	ETH020	Ethiopia	Konso	Leaf	MS	18980.014	4.846	81.087	9148.544	1.537	15.643
ETF0048	ETH020	Ethiopia	Konso	Leaf	MS	18893.696	4.849	80.141	9165.597	1.536	15.708
ETF0048	ETH020	Ethiopia	Konso	Leaf	MS	18849.012	4.810	78.832	9127.855	1.516	15.639
ETF0051	ETH021	Ethiopia	Konso	Leaf	MS	17932.417	4.402	76.210	5903.007	0.075	14.536
ETF0051	ETH021	Ethiopia	Konso	Leaf	MS	18431.480	4.780	78.308	6132.503	0.075	15.061
ETF0051	ETH021	Ethiopia	Konso	Leaf	MS	18726.541	4.636	79.409	6238.584	0.055	15.113
ETF0053	ETH022	Ethiopia	Konso	Leaf	MS	28906.285	3.373	169.644	9123.912	0.224	10.492
ETF0053	ETH022	Ethiopia	Konso	Leaf	MS	29478.003	3.385	180.376	9326.026	0.218	10.839
ETF0053	ETH022	Ethiopia	Konso	Leaf	MS	28331.946	3.228	157.242	8910.539	0.213	10.266
ETF0054	ETH023	Ethiopia	Konso	Leaf	MS	16854.850	4.102	88.956	5466.461	0.100	17.557
ETF0054	ETH023	Ethiopia	Konso	Leaf	MS	16734.620	4.117	88.913	5418.323	0.096	17.599
ETF0054	ETH023	Ethiopia	Konso	Leaf	MS	17137.274	4.184	87.124	5487.590	0.093	17.894
ETF0055	ETH024	Ethiopia	Konso	Leaf	MS	18498.617	3.958	112.925	5632.698	0.039	18.130
ETF0055	ETH024	Ethiopia	Konso	Leaf	MS	19710.679	4.178	117.326	5978.377	0.036	19.440
ETF0055	ETH024	Ethiopia	Konso	Leaf	MS	18818.304	4.050	149.883	5797.847	0.034	18.429
ETF0057	SeGen	Ethiopia	Konso	Leaf	MS	23161.463	6.396	215.927	6524.215	4.749	24.007
ETF0057	SeGen	Ethiopia	Konso	Leaf	MS	23567.206	6.452	230.554	6613.852	4.675	24.467
ETF0057	SeGen	Ethiopia	Konso	Leaf	MS	22969.115	6.549	223.426	6395.533	4.504	23.816
ETF0058	SeGen	Ethiopia	Konso	Leaf	MS	23010.039	6.424	213.468	6563.324	4.889	23.968
ETF0058	SeGen	Ethiopia	Konso	Leaf	MS	22592.963	6.323	239.516	6455.438	4.764	23.831
ETF0058	SeGen	Ethiopia	Konso	Leaf	MS	19204.694	5.608	226.094	5516.355	4.002	20.157
Eth-Haw-1	Eth-Haw-1	Ethiopia	Hawasa	Leaf	MS	12567.078	4.800	167.992	4290.476	1.177	22.555
Eth-Haw-1	Eth-Haw-1	Ethiopia	Hawasa	Leaf	MS	12847.765	4.923	197.376	4440.105	1.159	23.191
Eth-Haw-1	Eth-Haw-1	Ethiopia	Hawasa	Leaf	MS	12582.709	4.829	168.816	4338.922	1.063	22.281
Eth-Haw-10	Eth-Haw-10	Ethiopia	Hawasa	Leaf	MS	18015.133	3.378	108.260	3426.194	2.067	29.287
Eth-Haw-10	Eth-Haw-10	Ethiopia	Hawasa	Leaf	MS	17713.314	3.311	104.170	3329.763	2.052	29.662
Eth-Haw-10	Eth-Haw-10	Ethiopia	Hawasa	Leaf	MS	17634.460	3.333	107.931	3370.135	1.988	29.117

Sample_ID	Household_ID	Country	Locality	Edible part	Species	Ca	Cu	Fe	Mg	Se	Zn
Eth-Haw-11	Eth-Haw-11	Ethiopia	Hawasa	Leaf	MS	28069.378	4.779	110.082	3232.101	1.842	24.596
Eth-Haw-11	Eth-Haw-11	Ethiopia	Hawasa	Leaf	MS	28273.572	4.838	114.508	3318.170	1.785	24.958
Eth-Haw-11	Eth-Haw-11	Ethiopia	Hawasa	Leaf	MS	27878.138	4.771	112.933	3268.913	1.771	24.951
Eth-Haw-12	Eth-Haw-12	Ethiopia	Hawasa	Leaf	MS	42641.533	4.354	87.848	4085.707	1.764	29.591
Eth-Haw-12	Eth-Haw-12	Ethiopia	Hawasa	Leaf	MS	43888.151	4.569	96.839	4202.209	1.738	30.684
Eth-Haw-12	Eth-Haw-12	Ethiopia	Hawasa	Leaf	MS	44215.387	4.469	91.385	4209.731	1.738	30.286
Eth-Haw-13	Eth-Haw-13	Ethiopia	Hawasa	Leaf	MS	30488.739	5.698	116.412	4768.384	1.810	22.958
Eth-Haw-13	Eth-Haw-13	Ethiopia	Hawasa	Leaf	MS	29829.224	5.741	111.104	4648.706	1.726	22.251
Eth-Haw-13	Eth-Haw-13	Ethiopia	Hawasa	Leaf	MS	29959.228	5.551	116.213	4664.166	1.719	22.921
Eth-Haw-14	Eth-Haw-14	Ethiopia	Hawasa	Leaf	MS	38446.573	6.801	155.588	6006.539	1.002	39.489
Eth-Haw-14	Eth-Haw-14	Ethiopia	Hawasa	Leaf	MS	38906.924	6.950	181.923	6135.213	1.002	39.760
Eth-Haw-14	Eth-Haw-14	Ethiopia	Hawasa	Leaf	MS	38090.738	6.766	151.965	5971.308	0.992	39.299
Eth-Haw-2	Eth-Haw-2	Ethiopia	Hawasa	Leaf	MS	11913.196	3.778	123.698	4789.797	1.132	24.650
Eth-Haw-2	Eth-Haw-2	Ethiopia	Hawasa	Leaf	MS	11321.374	3.620	118.781	4539.757	1.092	23.491
Eth-Haw-2	Eth-Haw-2	Ethiopia	Hawasa	Leaf	MS	11346.083	3.616	111.932	4528.689	1.088	23.515
Eth-Haw-3	Eth-Haw-3	Ethiopia	Hawasa	Leaf	MS	12045.625	4.427	144.615	2988.205	0.997	29.359
Eth-Haw-3	Eth-Haw-3	Ethiopia	Hawasa	Leaf	MS	12168.879	4.460	134.394	3027.652	0.990	29.403
Eth-Haw-3	Eth-Haw-3	Ethiopia	Hawasa	Leaf	MS	12264.547	5.039	134.076	3054.605	0.952	29.937
Eth-Haw-4	Eth-Haw-4	Ethiopia	Hawasa	Leaf	MS	10083.099	3.857	154.181	3437.626	1.942	26.515
Eth-Haw-4	Eth-Haw-4	Ethiopia	Hawasa	Leaf	MS	10113.822	3.846	148.433	3385.700	1.903	26.328
Eth-Haw-4	Eth-Haw-4	Ethiopia	Hawasa	Leaf	MS	9718.859	3.662	148.236	3261.127	1.864	25.303
Eth-Haw-5	Eth-Haw-5	Ethiopia	Hawasa	Leaf	MS	17676.878	4.228	99.111	3811.284	1.543	20.455
Eth-Haw-5	Eth-Haw-5	Ethiopia	Hawasa	Leaf	MS	17369.271	4.181	87.759	3797.364	1.512	19.993
Eth-Haw-5	Eth-Haw-5	Ethiopia	Hawasa	Leaf	MS	16960.501	4.083	82.601	3716.356	1.492	19.795
Eth-Haw-6	Eth-Haw-6	Ethiopia	Hawasa	Leaf	MS	12165.973	2.463	82.282	4858.968	0.659	24.531
Eth-Haw-6	Eth-Haw-6	Ethiopia	Hawasa	Leaf	MS	12092.751	2.473	78.418	4802.818	0.640	24.477
Eth-Haw-6	Eth-Haw-6	Ethiopia	Hawasa	Leaf	MS	11591.290	2.398	72.341	4595.329	0.600	23.957
Eth-Haw-7	Eth-Haw-7	Ethiopia	Hawasa	Leaf	MS	9699.690	3.718	250.121	2339.708	1.378	23.850
Eth-Haw-7	Eth-Haw-7	Ethiopia	Hawasa	Leaf	MS	9625.449	3.487	236.572	2325.772	1.347	24.206
Eth-Haw-7	Eth-Haw-7	Ethiopia	Hawasa	Leaf	MS	9610.757	3.453	246.007	2326.544	1.338	24.339
Eth-Haw-8	Eth-Haw-8	Ethiopia	Hawasa	Leaf	MS	27623.955	5.979	146.114	5340.339	1.400	20.202

Sample_ID	Household_ID	Country	Locality	Edible part	Species	Ca	Cu	Fe	Mg	Se	Zn
Eth-Haw-8	Eth-Haw-8	Ethiopia	Hawasa	Leaf	MS	28839.577	6.318	159.026	5711.323	1.323	21.069
Eth-Haw-8	Eth-Haw-8	Ethiopia	Hawasa	Leaf	MS	28601.195	6.315	151.483	5635.167	1.245	20.628
Eth-Haw-9	Eth-Haw-9	Ethiopia	Hawasa	Leaf	MS	9112.740	3.908	116.399	3317.954	1.254	33.178
Eth-Haw-9	Eth-Haw-9	Ethiopia	Hawasa	Leaf	MS	8793.649	3.827	107.032	3210.956	1.188	32.522
Eth-Haw-9	Eth-Haw-9	Ethiopia	Hawasa	Leaf	MS	9206.66	3.811	111.149	3294.575	1.183	33.315
F-MO-15-MBO	15	Kenya	Mbololo	Flower	MO	4497.384	4.479	159.43	3490.025	3.63	28.027
F-MO-15-MBO	15	Kenya	Mbololo	Flower	MO	4579.633	6.248	187.556	3919.239	3.923	30.211
F-MO-15-MBO	15	Kenya	Mbololo	Flower	MO	5005.967	5.541	180.738	3927.649	3.906	30.218
F-MO-16-MBO	16	Kenya	Mbololo	Flower	MO	5786.655	8.386	414.631	3787.258	3.567	29.349
F-MO-16-MBO	16	Kenya	Mbololo	Flower	MO	5963.275	7.32	329.045	3946.233	3.228	27.675
F-MO-16-MBO	16	Kenya	Mbololo	Flower	MO	6687.503	7.435	410.003	4432.406	3.805	31.139
F-MO-17-MBO	17	Kenya	Mbololo	Flower	MO	2609.151	9.595	297.127	2566.498	0.896	46.939
F-MO-17-MBO	17	Kenya	Mbololo	Flower	MO	2656.036	8.881	283.154	2563.734	0.843	46.895
F-MO-17-MBO	17	Kenya	Mbololo	Flower	MO	2757.752	8.721	311.504	2646.619	0.865	47.91
F-MO-18-MBO	18	Kenya	Mbololo	Flower	MO	4693.665	7.365	170.247	2892.701	0.533	29.246
F-MO-18-MBO	18	Kenya	Mbololo	Flower	MO	4905.069	8.995	194.689	3146.367	0.6	32.647
F-MO-18-MBO	18	Kenya	Mbololo	Flower	MO	5303.981	8.49	184.895	3282.183	0.578	32.289
F-MO-19-MBO	19	Kenya	Mbololo	Flower	MO	4927.597	7.024	400.241	3657.204	2.365	28.819
F-MO-19-MBO	19	Kenya	Mbololo	Flower	MO	4952.643	6.032	334.864	3626.416	2.225	27.201
F-MO-19-MBO	19	Kenya	Mbololo	Flower	MO	5140.866	5.704	417.081	3700.293	2.357	29.798
F-MO-20-MBO	20	Kenya	Mbololo	Flower	MO	4469.452	6.075	292.099	3367.245	1.483	35.835
F-MO-20-MBO	20	Kenya	Mbololo	Flower	MO	5335.36	6.758	272.548	4009.318	1.593	39.229
F-MO-20-MBO	20	Kenya	Mbololo	Flower	MO	5335.86	5.919	293.909	4212.426	1.607	39.382
F-MO-21-MBO	21	Kenya	Mbololo	Flower	MO	6030.896	3.91	156.05	3273.687	5.567	22.697
F-MO-21-MBO	21	Kenya	Mbololo	Flower	MO	6127.414	4.345	165.477	3503.256	5.895	24.533
F-MO-21-MBO	21	Kenya	Mbololo	Flower	MO	6309.861	3.348	158.751	3439.983	5.801	23.896
F-MO-22-MBO	22	Kenya	Mbololo	Flower	MO	4424.681	9.231	328.212	4462.469	1.177	43.892
F-MO-22-MBO	22	Kenya	Mbololo	Flower	MO	4466.54	8.138	324.228	3888.692	1.07	43.506
F-MO-22-MBO	22	Kenya	Mbololo	Flower	MO	4504.712	8.909	326.348	4307.991	1.074	42.424
F-MO-23-MBO	23	Kenya	Mbololo	Flower	MO	3337.187	4.488	104.519	2493.811	6.447	31.592
F-MO-23-MBO	23	Kenya	Mbololo	Flower	MO	3354.886	5.57	105.942	2621.171	6.658	33.097

Sample_ID	Household_ID	Country	Locality	Edible part	Species	Ca	Cu	Fe	Mg	Se	Zn
F-MO-23-MBO	23	Kenya	Mbololo	Flower	MO	3416.381	6.494	107.185	2554.175	6.485	32.111
F-MO-24-MBO	24	Kenya	Mbololo	Flower	MO	3228.349	2.394	123.384	2239.241	0.849	23.214
F-MO-24-MBO	24	Kenya	Mbololo	Flower	MO	3406.74	3.495	132.615	2404.336	0.94	24.915
F-MO-24-MBO	24	Kenya	Mbololo	Flower	MO	3416.854	4.503	132.928	2373.2	0.885	24.69
F-MO-25-MBO	25	Kenya	Mbololo	Flower	MO	2962.64	5.024	120.808	2425.892	2.527	19.728
F-MO-25-MBO	25	Kenya	Mbololo	Flower	MO	3188.66	4.131	132.236	2439.599	2.49	20.885
F-MO-25-MBO	25	Kenya	Mbololo	Flower	MO	3195.855	6.333	135.163	2402.788	2.457	21.78
F-MO-26-MBO	26	Kenya	Mbololo	Flower	MO	6101.131	6.096	244.399	3616.114	0.702	30.874
F-MO-26-MBO	26	Kenya	Mbololo	Flower	MO	6186.211	5.696	237.184	3411.872	0.642	28.332
F-MO-26-MBO	26	Kenya	Mbololo	Flower	MO	6193.213	7.411	236.339	3446.835	0.656	28.877
F-MO-27-MBO	27	Kenya	Mbololo	Flower	MO	3258.68	4.16	145.507	2666.007	0.678	28.191
F-MO-27-MBO	27	Kenya	Mbololo	Flower	MO	3297.498	3.859	148.929	2648.924	0.646	29.887
F-MO-27-MBO	27	Kenya	Mbololo	Flower	MO	3468.021	5.396	154.248	2778.91	0.662	30.333
F-MO-28-MBO	28	Kenya	Mbololo	Flower	MO	5887.473	8.483	228.167	3264.125	1.086	30.094
F-MO-28-MBO	28	Kenya	Mbololo	Flower	MO	6112.017	7.048	237.279	3448.73	1.19	31.729
F-MO-28-MBO	28	Kenya	Mbololo	Flower	MO	6351.303	8.291	233.187	3919.157	1.284	33.204
F-MO-29-MBO	29	Kenya	Mbololo	Flower	MO	3233.664	5.716	194.861	2684.846	12.968	31.509
F-MO-29-MBO	29	Kenya	Mbololo	Flower	MO	3600.099	8.82	220.154	2775.762	12.768	33.562
F-MO-29-MBO	29	Kenya	Mbololo	Flower	MO	3643.255	5.264	226.165	2833.273	13.254	34.224
F-MO-30-MBO	30	Kenya	Mbololo	Flower	MO	2796.071	4.883	119.712	2243.29	3.311	28.004
F-MO-30-MBO	30	Kenya	Mbololo	Flower	MO	2867.629	5.845	113.72	2371.417	3.627	28.314
F-MO-30-MBO	30	Kenya	Mbololo	Flower	MO	2974.383	7.742	123.667	2406.609	3.512	30.032
F-MO-36-BAR	36	Kenya	Baringo	Flower	MO	3884.701	7.894	662.84	2079.869	0.427	31.356
F-MO-36-BAR	36	Kenya	Baringo	Flower	MO	4319.396	11.478	799.681	2227.566	0.439	37.057
F-MO-36-BAR	36	Kenya	Baringo	Flower	MO	4513.012	8.716	787.517	2330.41	0.463	35.336
F-MO-37-RAM	37	Kenya	Ramogi	Flower	MO	3039.266	7.531	258.917	3458.354	0.108	32.741
F-MO-37-RAM	37	Kenya	Ramogi	Flower	MO	3352.103	10.055	286.633	3548.931	0.104	36.397
F-MO-37-RAM	37	Kenya	Ramogi	Flower	MO	3470.888	8.223	287.269	3614.271	0.108	36.32
F-MO-38-RAM	38	Kenya	Ramogi	Flower	MO	4373.549	6.629	159.047	3054.727	1.797	35.207
F-MO-38-RAM	38	Kenya	Ramogi	Flower	MO	4680.753	8.829	166.642	3220.051	1.815	38.16
F-MO-38-RAM	38	Kenya	Ramogi	Flower	MO	4716.692	6.713	167.877	3236.499	1.803	37.124

Sample_ID	Household_ID	Country	Locality	Edible part	Species	Ca	Cu	Fe	Mg	Se	Zn
F-MO-39-RAM	39	Kenya	Ramogi	Flower	MO	3309.118	6.223	177.041	2184.816	1.326	30.307
F-MO-39-RAM	39	Kenya	Ramogi	Flower	MO	3669.809	8.609	186.954	2342.238	1.4	34.089
F-MO-39-RAM	39	Kenya	Ramogi	Flower	MO	3950.615	7.199	207.425	2523.729	1.481	36.358
F-MO-40-RAM	40	Kenya	Ramogi	Flower	MO	2252.02	4.468	219.008	1900.826	0.871	28.352
F-MO-40-RAM	40	Kenya	Ramogi	Flower	MO	2259.792	6.495	224.981	1897.182	0.834	29.466
F-MO-40-RAM	40	Kenya	Ramogi	Flower	MO	2273.929	4.925	225.827	1889.019	0.812	28.189
F-MO-41-RAM	41	Kenya	Ramogi	Flower	MO	3616.044	8.612	315.769	2327.455	0.137	43.235
F-MO-41-RAM	41	Kenya	Ramogi	Flower	MO	3825.624	7.25	331.504	2518.048	0.173	44.42
F-MO-41-RAM	41	Kenya	Ramogi	Flower	MO	4032.299	6.875	336.031	2578.572	0.15	45.473
F-MO-41-RAM	41	Kenya	Ramogi	Flower	MO	4110.155	8.286	339.908	2625.05	0.155	47.04
F-MO-42-RAM	42	Kenya	Ramogi	Flower	MO	3426.173	7.498	436.797	2792.441	0.341	44.096
F-MO-42-RAM	42	Kenya	Ramogi	Flower	MO	3502.505	7.799	381.73	2805.902	0.331	43.75
F-MO-42-RAM	42	Kenya	Ramogi	Flower	MO	3629.061	9.419	432.368	2886.764	0.337	45.966
F-MO-44-RAM	44	Kenya	Ramogi	Flower	MO	1941.854	5.58	209.905	1973.603	0.039	22.257
F-MO-44-RAM	44	Kenya	Ramogi	Flower	MO	1989.511	5.836	216.972	1960.587	0.024	22.04
F-MO-44-RAM	44	Kenya	Ramogi	Flower	MO	2120.492	8.477	219.069	2096.043	0.026	23.66
F-MO-45-MAL	45	Kenya	Malindi	Flower	MO	3194.935	8.744	179.867	2118.523	0.462	39.131
F-MO-45-MAL	45	Kenya	Malindi	Flower	MO	3520.536	9.805	203.559	2275.522	0.442	42.256
F-MO-45-MAL	45	Kenya	Malindi	Flower	MO	3532.144	11.949	204.531	2249.365	0.441	41.859
F-MO-47-MAL	47	Kenya	Malindi	Flower	MO	4894.77	6.411	192.594	2644.624	3.024	36.262
F-MO-47-MAL	47	Kenya	Malindi	Flower	MO	5159.593	6.941	197.263	2698.219	3.029	38.221
F-MO-47-MAL	47	Kenya	Malindi	Flower	MO	5214.618	9.115	194.343	2737.411	2.998	40.012
F-MO-48-MAL	48	Kenya	Malindi	Flower	MO	2949.51	3.712	134.216	2460.926	1.488	23.821
F-MO-48-MAL	48	Kenya	Malindi	Flower	MO	3162.054	4.63	145.436	2503.59	1.417	24.073
F-MO-48-MAL	48	Kenya	Malindi	Flower	MO	3207.128	7.072	146.505	2580.064	1.427	26.592
F-MO-4-KIB	4	Kenya	Kibwezi	Flower	MO	5972.373	6.377	788.489	3223.073	1.911	35.335
F-MO-4-KIB	4	Kenya	Kibwezi	Flower	MO	6096.664	4.886	885.793	3264.857	1.807	33.085
F-MO-4-KIB	4	Kenya	Kibwezi	Flower	MO	6545.012	5.74	965.625	3358.391	1.885	34.665
F-MO-51-MAL	51	Kenya	Malindi	Flower	MO	1746.788	4.742	67.275	2611.179	8.709	36.397
F-MO-51-MAL	51	Kenya	Malindi	Flower	MO	1888.882	5.743	71.14	2644.219	8.399	36.583
F-MO-51-MAL	51	Kenya	Malindi	Flower	MO	1889.927	7.82	72.804	2761.93	8.622	38.239

Sample_ID	Household_ID	Country	Locality	Edible part	Species	Ca	Cu	Fe	Mg	Se	Zn
F-MO-52-MAL	52	Kenya	Malindi	Flower	MO	3418.255	7.299	58.734	2254.281	6.06	29.063
F-MO-52-MAL	52	Kenya	Malindi	Flower	MO	3537.135	10.236	61.183	2353.909	6.242	30.7
F-MO-52-MAL	52	Kenya	Malindi	Flower	MO	3580.663	6.785	62.892	2441.169	6.58	32.123
F-MO-53-MAL	53	Kenya	Malindi	Flower	MO	1283.846	3.223	60.945	2176.735	1.874	29.758
F-MO-53-MAL	53	Kenya	Malindi	Flower	MO	1522.362	3.4	72.305	2497.697	2.025	34.435
F-MO-53-MAL	53	Kenya	Malindi	Flower	MO	1595.743	4.81	77.265	2633.756	2.213	36.441
F-MO-55-MAL	55	Kenya	Malindi	Flower	MO	2392.436	4.809	71.587	2525.222	9.292	31.653
F-MO-55-MAL	55	Kenya	Malindi	Flower	MO	2612.513	4.678	76.575	2619.234	8.773	31.96
F-MO-55-MAL	55	Kenya	Malindi	Flower	MO	2691.98	6.121	79.084	2695.519	9.194	34.464
F-MO-57-UKU	57	Kenya	Ukunda	Flower	MO	2066.826	4.321	61.232	1990.889	0.822	24.408
F-MO-57-UKU	57	Kenya	Ukunda	Flower	MO	2140.625	4.04	62.283	2009.72	0.814	25.094
F-MO-57-UKU	57	Kenya	Ukunda	Flower	MO	2256.202	5.317	65.526	2037.311	0.765	25.433
F-MO-61-UKU	61	Kenya	Ukunda	Flower	MO	1531.179	3.664	882.844	2297.115	1.642	33.722
F-MO-61-UKU	61	Kenya	Ukunda	Flower	MO	1596.162	4.295	826.178	2489.915	1.858	36.752
F-MO-61-UKU	61	Kenya	Ukunda	Flower	MO	1682.9	4.96	966.881	2504.662	1.806	36.494
F-MO-62-UKU	62	Kenya	Ukunda	Flower	MO	1570.196	3.54	1192.202	2345.73	6.335	30.552
F-MO-62-UKU	62	Kenya	Ukunda	Flower	MO	1574.91	3.053	1228.793	2284.673	6.026	29.499
F-MO-62-UKU	62	Kenya	Ukunda	Flower	MO	1676.4	4.405	1301.033	2422.574	6.299	33.493
GP-MO-10-KIB	10	Kenya	Kibwezi	Immature pod	MO	3940.505	7.036	68.31	3056.588	3.635	32.224
GP-MO-10-KIB	10	Kenya	Kibwezi	Immature pod	MO	4307.266	7.168	73.007	3120.337	3.634	33.569
GP-MO-10-KIB	10	Kenya	Kibwezi	Immature pod	MO	4483.001	7.855	71.907	3154.624	3.621	33.614
GP-MO-15-MBO	15	Kenya	Mbololo	Immature pod	MO	3680.379	3.878	47.512	2831.627	2.325	18.969
GP-MO-15-MBO	15	Kenya	Mbololo	Immature pod	MO	3781.111	3.85	49.707	2811.776	2.256	19.699
GP-MO-15-MBO	15	Kenya	Mbololo	Immature pod	MO	3889.057	3.926	51.714	2959.566	2.316	20.471
GP-MO-16-MBO	16	Kenya	Mbololo	Immature pod	MO	6517.384	6.166	70.861	4914.723	5.77	29.412
GP-MO-16-MBO	16	Kenya	Mbololo	Immature pod	MO	7273.459	6.437	80.876	5283.501	6.119	30.974
GP-MO-16-MBO	16	Kenya	Mbololo	Immature pod	MO	7360.51	7.149	81.713	5309.903	6.044	32.708
GP-MO-17-MBO	17	Kenya	Mbololo	Immature pod	MO	2855.301	4.548	44.248	2186.079	0.32	24.266
GP-MO-17-MBO	17	Kenya	Mbololo	Immature pod	MO	2893.922	4.344	45.904	2231.906	0.328	24.318
GP-MO-17-MBO	17	Kenya	Mbololo	Immature pod	MO	2915.564	4.773	46.101	2371.146	0.364	26.394
GP-MO-18-MBO	18	Kenya	Mbololo	Immature pod	MO	5883.989	6.2	44.308	4122.099	0.457	29.453

Sample_ID	Household_ID	Country	Locality	Edible part	Species	Ca	Cu	Fe	Mg	Se	Zn
GP-MO-18-MBO	18	Kenya	Mbololo	Immature pod	MO	6400.98	6.098	47.505	4327.608	0.438	28.876
GP-MO-18-MBO	18	Kenya	Mbololo	Immature pod	MO	6794.051	6.783	52.568	4559.589	0.469	31.369
GP-MO-19-MBO	19	Kenya	Mbololo	Immature pod	MO	5624.602	6.965	68.385	3877.212	1.583	22.661
GP-MO-19-MBO	19	Kenya	Mbololo	Immature pod	MO	5895.772	7.221	75.841	4427.159	1.724	24.65
GP-MO-19-MBO	19	Kenya	Mbololo	Immature pod	MO	6013.911	7.209	76.567	4359.635	1.708	24.371
GP-MO-20-MBO	20	Kenya	Mbololo	Immature pod	MO	3779.923	2.983	34.121	2363.784	2.846	14.641
GP-MO-20-MBO	20	Kenya	Mbololo	Immature pod	MO	3934.589	3.199	36.151	2574.755	3.176	15.456
GP-MO-20-MBO	20	Kenya	Mbololo	Immature pod	MO	4065.939	2.761	37.149	2549.391	2.989	14.984
GP-MO-21-MBO	21	Kenya	Mbololo	Immature pod	MO	4965.59	3.379	33.364	2911.327	4.409	17.951
GP-MO-21-MBO	21	Kenya	Mbololo	Immature pod	MO	5662.303	3.687	37.137	3099.08	4.533	18.396
GP-MO-21-MBO	21	Kenya	Mbololo	Immature pod	MO	5769.227	3.365	38.987	3189.64	4.518	18.759
GP-MO-22-MBO	22	Kenya	Mbololo	Immature pod	MO	3956.287	8.411	51.634	3957.079	1.092	37.229
GP-MO-22-MBO	22	Kenya	Mbololo	Immature pod	MO	4425.356	8.972	57.311	4379.186	1.106	40.25
GP-MO-22-MBO	22	Kenya	Mbololo	Immature pod	MO	4510.85	9.247	58.24	4317.309	1.169	39.56
GP-MO-23-MBO	23	Kenya	Mbololo	Immature pod	MO	3305.965	4.22	39.986	2521.178	4.967	23.8
GP-MO-23-MBO	23	Kenya	Mbololo	Immature pod	MO	3870.96	4.077	48.285	2727.446	5.189	25.749
GP-MO-23-MBO	23	Kenya	Mbololo	Immature pod	MO	4153.358	4.752	50.519	2899.868	5.636	27.366
GP-MO-24-MBO	24	Kenya	Mbololo	Immature pod	MO	4940.486	4.283	62.271	4160.242	0.819	28.003
GP-MO-24-MBO	24	Kenya	Mbololo	Immature pod	MO	4985.572	3.66	63.015	3968.115	0.743	25.534
GP-MO-24-MBO	24	Kenya	Mbololo	Immature pod	MO	5108.442	3.809	62.192	4192.118	0.771	26.68
GP-MO-27-MBO	27	Kenya	Mbololo	Immature pod	MO	3058.468	2.265	47.692	2691.849	0.564	23.715
GP-MO-27-MBO	27	Kenya	Mbololo	Immature pod	MO	3305.826	2.63	53.372	2942.246	0.617	27.211
GP-MO-27-MBO	27	Kenya	Mbololo	Immature pod	MO	3547.6	3.005	55.374	3360.046	0.716	29.781
GP-MO-29-MBO	29	Kenya	Mbololo	Immature pod	MO	4434.898	3.833	53.353	2740.778	17.828	24.393
GP-MO-29-MBO	29	Kenya	Mbololo	Immature pod	MO	4842.355	3.866	58.064	2858.372	17.673	25.483
GP-MO-29-MBO	29	Kenya	Mbololo	Immature pod	MO	4928.844	3.778	58.364	2863.721	17.881	24.612
GP-MO-2-KIB	2	Kenya	Kibwezi	Immature pod	MO	3786.676	6.744	77.62	4295.933	2.913	35.198
GP-MO-2-KIB	2	Kenya	Kibwezi	Immature pod	MO	4044.542	6.86	85.771	4210.662	3.073	38.202
GP-MO-2-KIB	2	Kenya	Kibwezi	Immature pod	MO	4053.58	6.773	84.153	4290.651	2.988	36.724
GP-MO-30-MBO	30	Kenya	Mbololo	Immature pod	MO	2581.518	5.366	43.153	1675.169	2.684	25.58
GP-MO-30-MBO	30	Kenya	Mbololo	Immature pod	MO	2819.222	5.377	47.821	1728.152	2.676	26.879

Sample_ID	Household_ID	Country	Locality	Edible part	Species	Ca	Cu	Fe	Mg	Se	Zn
GP-MO-30-MBO	30	Kenya	Mbololo	Immature pod	MO	2837.689	5.604	48.422	1787.165	2.725	27.557
GP-MO-38-RAM	38	Kenya	Ramogi	Immature pod	MO	4068.127	7.511	68.99	3462.641	1.811	35.539
GP-MO-38-RAM	38	Kenya	Ramogi	Immature pod	MO	4199.811	7.532	70.617	3305.587	1.722	36.32
GP-MO-38-RAM	38	Kenya	Ramogi	Immature pod	MO	4246	7.675	71.322	3441.914	1.744	36.178
GP-MO-39-RAM	39	Kenya	Ramogi	Immature pod	MO	5800.388	7.483	72.741	3016.222	1.009	40.648
GP-MO-39-RAM	39	Kenya	Ramogi	Immature pod	MO	6106.585	7.66	76.624	3191.195	1.072	42.858
GP-MO-39-RAM	39	Kenya	Ramogi	Immature pod	MO	6133.851	8.087	75.793	3396.619	1.177	45.193
GP-MO-3-KIB	3	Kenya	Kibwezi	Immature pod	MO	1804.426	8.088	79.224	2411.571	3.246	31.5
GP-MO-3-KIB	3	Kenya	Kibwezi	Immature pod	MO	1928.365	8.364	83.958	2452.24	3.156	31.497
GP-MO-3-KIB	3	Kenya	Kibwezi	Immature pod	MO	1973.04	8.428	91.862	2546.354	3.238	33.734
GP-MO-40-RAM	40	Kenya	Ramogi	Immature pod	MO	2582.393	4.603	58.236	2183.328	0.405	28.158
GP-MO-40-RAM	40	Kenya	Ramogi	Immature pod	MO	2618.799	4.66	59.546	2270.836	0.423	29.795
GP-MO-40-RAM	40	Kenya	Ramogi	Immature pod	MO	2620.607	4.676	59.516	2276.098	0.443	28.719
GP-MO-41-RAM	41	Kenya	Ramogi	Immature pod	MO	3104.288	6.761	76.762	2563.099	0.194	37.491
GP-MO-41-RAM	41	Kenya	Ramogi	Immature pod	MO	3198.656	6.681	75.978	2545.285	0.176	37.91
GP-MO-41-RAM	41	Kenya	Ramogi	Immature pod	MO	3362.792	7.114	80.103	2616.981	0.179	38.628
GP-MO-44-RAM	44	Kenya	Ramogi	Immature pod	MO	2212.502	7.525	60.757	2605.419	0.035	32.307
GP-MO-44-RAM	44	Kenya	Ramogi	Immature pod	MO	2275.047	7.39	62.268	2608.284	0.023	32.049
GP-MO-44-RAM	44	Kenya	Ramogi	Immature pod	MO	2388.302	7.988	65.255	2704.087	0.024	33.37
GP-MO-45-MAL	45	Kenya	Malindi	Immature pod	MO	2254.769	3.963	48.076	1681.705	0.604	17.84
GP-MO-45-MAL	45	Kenya	Malindi	Immature pod	MO	2777.787	4.543	59.202	1977.639	0.673	21.417
GP-MO-45-MAL	45	Kenya	Malindi	Immature pod	MO	2819.509	4.659	60.577	1988.871	0.7	21.263
GP-MO-47-MAL	47	Kenya	Malindi	Immature pod	MO	1526.943	3.19	53.01	1570.784	3.343	24.783
GP-MO-47-MAL	47	Kenya	Malindi	Immature pod	MO	1755.799	3.666	59.6	1852.936	4.013	27.291
GP-MO-47-MAL	47	Kenya	Malindi	Immature pod	MO	1760.121	3.387	60.561	1807.133	3.852	27.498
GP-MO-47-MAL	47	Kenya	Malindi	Immature pod	MO	1839.367	3.747	64.368	1874.951	3.883	29.355
GP-MO-60-UKU	60	Kenya	Ukunda	Immature pod	MO	2673.113	1.775	31.934	1645.451	0.732	19.545
GP-MO-60-UKU	60	Kenya	Ukunda	Immature pod	MO	2835.335	2.028	35.422	1744.218	0.784	20.751
GP-MO-60-UKU	60	Kenya	Ukunda	Immature pod	MO	2905.459	2.11	36.241	1852.194	0.86	22.598
GP-MO-61-UKU	61	Kenya	Ukunda	Immature pod	MO	1188.528	4.323	98.501	1938.945	1.318	30.359
GP-MO-61-UKU	61	Kenya	Ukunda	Immature pod	MO	1231.637	4.754	101.386	2032.62	1.416	31.815

Sample_ID	Household_ID	Country	Locality	Edible part	Species	Ca	Cu	Fe	Mg	Se	Zn
GP-MO-61-UKU	61	Kenya	Ukunda	Immature pod	MO	1250.593	4.596	105.606	2000.79	1.374	31.643
GP-MO-62-UKU	62	Kenya	Ukunda	Immature pod	MO	814.293	2.882	99.683	1525.816	4.109	20.531
GP-MO-62-UKU	62	Kenya	Ukunda	Immature pod	MO	858.333	2.588	96.214	1533.65	4.032	20.802
GP-MO-62-UKU	62	Kenya	Ukunda	Immature pod	MO	878.344	2.793	102.195	1557.844	4.088	20.724
GP-MO-7-KIB	7	Kenya	Kibwezi	Immature pod	MO	5188.329	8.13	75.74	3157.484	7.591	27.954
GP-MO-7-KIB	7	Kenya	Kibwezi	Immature pod	MO	5310.338	8.437	78.773	3187.649	7.227	28.513
GP-MO-7-KIB	7	Kenya	Kibwezi	Immature pod	MO	5460.419	8.132	84.385	3148.342	7.262	28.452
L-MO-10-KIB	10	Kenya	Kibwezi	Leaf	MO	31673.099	7.179	166.366	6741.849	11.436	36.074
L-MO-10-KIB	10	Kenya	Kibwezi	Leaf	MO	29372.903	6.795	161.272	6416.253	11.417	33.495
L-MO-10-KIB	10	Kenya	Kibwezi	Leaf	MO	30695.952	6.631	163.86	6512.32	11.077	32.656
L-MO-11-KIB	11	Kenya	Kibwezi	Leaf	MO	18882.689	9.615	180.384	4473.365	3.274	28.141
L-MO-11-KIB	11	Kenya	Kibwezi	Leaf	MO	18423.372	9.706	178.853	4217.944	3.183	28.157
L-MO-11-KIB	11	Kenya	Kibwezi	Leaf	MO	15840.822	8.38	153.034	3837.27	3.081	24.427
L-MO-12-KIB	12	Kenya	Kibwezi	Leaf	MO	7823.938	12.485	155.711	5259.802	2.094	60.576
L-MO-12-KIB	12	Kenya	Kibwezi	Leaf	MO	7663.545	12.352	155.44	5138.666	2.089	60.613
L-MO-12-KIB	12	Kenya	Kibwezi	Leaf	MO	7076.591	11.237	135.523	4779.002	2.065	54.96
L-MO-13-KIB	13	Kenya	Kibwezi	Leaf	MO	7485.394	8.981	251.299	4902.862	2.073	60.827
L-MO-13-KIB	13	Kenya	Kibwezi	Leaf	MO	6742.092	8.168	215.499	4418.04	2.027	53.965
L-MO-13-KIB	13	Kenya	Kibwezi	Leaf	MO	6884.77	8.37	229.255	3981.241	2.024	55.736
L-MO-14-KIB	14	Kenya	Kibwezi	Leaf	MO	12832.067	10.275	269.69	4034.907	2.005	42.797
L-MO-14-KIB	14	Kenya	Kibwezi	Leaf	MO	11332.353	9.184	233.972	4057.159	1.92	37.38
L-MO-14-KIB	14	Kenya	Kibwezi	Leaf	MO	11794.425	9.579	248.422	4018.704	1.887	39.709
L-MO-15-MBO	15	Kenya	Mbololo	Leaf	MO	36058.669	5.533	186.197	11166.134	4.544	29.731
L-MO-15-MBO	15	Kenya	Mbololo	Leaf	MO	35507.026	5.381	187.277	10978.547	4.494	29.31
L-MO-15-MBO	15	Kenya	Mbololo	Leaf	MO	32306.801	5.21	167.772	10326.476	4.447	27.232
L-MO-16-MBO	16	Kenya	Mbololo	Leaf	MO	14695.689	6.762	132.431	6146.202	7.217	39.102
L-MO-16-MBO	16	Kenya	Mbololo	Leaf	MO	13417.963	6.163	117.961	5558.353	6.755	33.855
L-MO-16-MBO	16	Kenya	Mbololo	Leaf	MO	14068.036	6.775	126.869	5901.688	6.715	37.466
L-MO-17-MBO	17	Kenya	Mbololo	Leaf	MO	14078.721	6.862	135.277	5680.373	1.473	51.867
L-MO-17-MBO	17	Kenya	Mbololo	Leaf	MO	14047.255	6.835	141.782	5629.676	1.36	52.502
L-MO-17-MBO	17	Kenya	Mbololo	Leaf	MO	13652.088	6.678	135.999	5516.217	1.321	51.981

Sample_ID	Household_ID	Country	Locality	Edible part	Species	Ca	Cu	Fe	Mg	Se	Zn
L-MO-18-MBO	18	Kenya	Mbololo	Leaf	MO	26809.023	5.892	166.363	5296.809	0.595	23.876
L-MO-18-MBO	18	Kenya	Mbololo	Leaf	MO	28404.179	6.081	179.696	5518.177	0.583	24.91
L-MO-18-MBO	18	Kenya	Mbololo	Leaf	MO	26904.411	5.688	150.4	5323.625	0.528	24.007
L-MO-19-MBO	19	Kenya	Mbololo	Leaf	MO	25590.146	6.918	309.299	8261.019	3.219	42.051
L-MO-19-MBO	19	Kenya	Mbololo	Leaf	MO	27155.243	7.217	306.213	8548.082	3.214	44.891
L-MO-19-MBO	19	Kenya	Mbololo	Leaf	MO	27958.073	7.504	322.693	8866.215	3.176	45.863
L-MO-1-KIB	1	Kenya	Kibwezi	Leaf	MO	26758.893	8.347	238.808	6252.532	3.462	30.425
L-MO-1-KIB	1	Kenya	Kibwezi	Leaf	MO	24803.956	7.839	215.784	5871.934	3.357	28.427
L-MO-1-KIB	1	Kenya	Kibwezi	Leaf	MO	26412.475	8.608	235.683	6152.89	3.311	30.384
L-MO-20-MBO	20	Kenya	Mbololo	Leaf	MO	23175.515	4.485	159.517	6935.062	5.837	39.999
L-MO-20-MBO	20	Kenya	Mbololo	Leaf	MO	24468.387	4.801	171.737	7352.189	5.751	43.33
L-MO-20-MBO	20	Kenya	Mbololo	Leaf	MO	22460.353	4.293	154.93	6684.767	5.491	38.261
L-MO-21-MBO	21	Kenya	Mbololo	Leaf	MO	29474.734	5.765	156.844	6361.921	11.7	18.32
L-MO-21-MBO	21	Kenya	Mbololo	Leaf	MO	28795.116	5.621	148.479	6230.118	11.359	18.213
L-MO-21-MBO	21	Kenya	Mbololo	Leaf	MO	29336.899	5.444	144.701	6177.276	10.711	17.764
L-MO-22-MBO	22	Kenya	Mbololo	Leaf	MO	20150.016	8.839	142.354	7263.003	2.645	55.759
L-MO-22-MBO	22	Kenya	Mbololo	Leaf	MO	18605.413	8.286	131.712	6770.412	2.579	52.514
L-MO-22-MBO	22	Kenya	Mbololo	Leaf	MO	17594.471	7.552	125.547	6258.216	2.489	46.901
L-MO-23-MBO	23	Kenya	Mbololo	Leaf	MO	16387.661	5.535	110.815	6561.418	17.771	38.071
L-MO-23-MBO	23	Kenya	Mbololo	Leaf	MO	17252.677	5.767	117.843	6881.083	17.633	38.85
L-MO-23-MBO	23	Kenya	Mbololo	Leaf	MO	16661.318	5.698	112.409	6734.62	16.591	38.631
L-MO-24-MBO	24	Kenya	Mbololo	Leaf	MO	31176.152	3.895	207.629	7567.828	1.648	39.458
L-MO-24-MBO	24	Kenya	Mbololo	Leaf	MO	31131.415	3.806	202.59	7665.506	1.602	18.315
L-MO-24-MBO	24	Kenya	Mbololo	Leaf	MO	28370.703	3.629	192.191	6747.332	1.598	15.951
L-MO-25-MBO	25	Kenya	Mbololo	Leaf	MO	13388.092	4.263	105.015	4450.013	6.065	27.263
L-MO-25-MBO	25	Kenya	Mbololo	Leaf	MO	13665.378	4.263	107.916	4201.148	6.026	29.568
L-MO-25-MBO	25	Kenya	Mbololo	Leaf	MO	13862.124	4.35	107.823	4645.779	5.797	28.943
L-MO-26-MBO	26	Kenya	Mbololo	Leaf	MO	19490.247	4.273	152.199	6361.622	3.545	27.45
L-MO-26-MBO	26	Kenya	Mbololo	Leaf	MO	20614.309	4.664	162.471	6660.295	3.448	29.558
L-MO-26-MBO	26	Kenya	Mbololo	Leaf	MO	19784.515	4.155	152.089	6346.033	3.33	30.753
L-MO-27-MBO	27	Kenya	Mbololo	Leaf	MO	37636.253	5.121	171.279	7420.598	1.226	22.858

Sample_ID	Household_ID	Country	Locality	Edible part	Species	Ca	Cu	Fe	Mg	Se	Zn
L-MO-27-MBO	27	Kenya	Mbololo	Leaf	MO	33191.225	4.642	142.73	6568.991	1.192	18.413
L-MO-27-MBO	27	Kenya	Mbololo	Leaf	MO	33725.106	4.706	152.331	6619.201	1.101	19.527
L-MO-28-MBO	28	Kenya	Mbololo	Leaf	MO	19390.266	4.844	297.77	6041.412	1.822	26.835
L-MO-28-MBO	28	Kenya	Mbololo	Leaf	MO	17490.805	4.431	269.554	5454.831	1.756	23.951
L-MO-28-MBO	28	Kenya	Mbololo	Leaf	MO	18635.09	4.647	287.904	5820.109	1.734	25.837
L-MO-29-MBO	29	Kenya	Mbololo	Leaf	MO	17027.639	4.581	155.422	6373.392	21.605	40.782
L-MO-29-MBO	29	Kenya	Mbololo	Leaf	MO	17234.336	4.525	156.821	6399.831	21.384	39.195
L-MO-29-MBO	29	Kenya	Mbololo	Leaf	MO	15521.466	4.229	142.354	5866.166	20.642	34.972
L-MO-2-KIB	2	Kenya	Kibwezi	Leaf	MO	16590.851	6.643	144.093	6844.481	8.189	44.834
L-MO-2-KIB	2	Kenya	Kibwezi	Leaf	MO	16177.71	6.477	137.68	6793.574	8.133	43.498
L-MO-2-KIB	2	Kenya	Kibwezi	Leaf	MO	10978.114	4.586	95.153	4622.684	5.29	31.853
L-MO-30-MBO	30	Kenya	Mbololo	Leaf	MO	29087.542	5.84	162.196	5100.494	4.75	34.75
L-MO-30-MBO	30	Kenya	Mbololo	Leaf	MO	26137.275	5.295	136.883	4628.913	4.569	31.368
L-MO-30-MBO	30	Kenya	Mbololo	Leaf	MO	26349.176	5.253	142.495	4672.79	4.271	34.667
L-MO-36-BAR	36	Kenya	Baringo	Leaf	MO	25208.653	6.003	587.88	4724.039	0.56	27.431
L-MO-36-BAR	36	Kenya	Baringo	Leaf	MO	27257.12	6.518	643.795	5128.312	0.557	30.258
L-MO-36-BAR	36	Kenya	Baringo	Leaf	MO	27065.564	6.618	666.808	5125.841	0.552	31.516
L-MO-37-RAM	37	Kenya	Ramogi	Leaf	MO	28388.398	6.319	442.806	5277.644	1.078	14.346
L-MO-37-RAM	37	Kenya	Ramogi	Leaf	MO	27024.156	6.091	387.01	4994.048	1.003	15.058
L-MO-37-RAM	37	Kenya	Ramogi	Leaf	MO	27227.711	6.029	367.532	5067.926	0.99	13.794
L-MO-38-RAM	38	Kenya	Ramogi	Leaf	MO	25608.13	7.094	249.712	7651.259	1.296	25.113
L-MO-38-RAM	38	Kenya	Ramogi	Leaf	MO	24546.166	6.896	244.902	7434.003	1.163	25.823
L-MO-38-RAM	38	Kenya	Ramogi	Leaf	MO	24796.91	6.711	240.474	7372.451	1.045	22.263
L-MO-39-RAM	39	Kenya	Ramogi	Leaf	MO	23825.317	5.915	241.564	5398.831	2.899	29.573
L-MO-39-RAM	39	Kenya	Ramogi	Leaf	MO	21775.871	5.458	218.76	4928.419	2.88	26.396
L-MO-39-RAM	39	Kenya	Ramogi	Leaf	MO	21839.815	5.56	210.991	4947.731	2.773	28.179
L-MO-3-KIB	3	Kenya	Kibwezi	Leaf	MO	20695.288	8.559	321.239	7003.731	7.593	34.169
L-MO-3-KIB	3	Kenya	Kibwezi	Leaf	MO	19989.18	8.212	299.311	6768.676	7.534	33.057
L-MO-3-KIB	3	Kenya	Kibwezi	Leaf	MO	20613.943	8.768	308.912	6993.867	7.281	34.449
L-MO-40-RAM	40	Kenya	Ramogi	Leaf	MO	8817.621	5.327	237.878	2581.811	1.003	32.381
L-MO-40-RAM	40	Kenya	Ramogi	Leaf	MO	8296.569	5.02	218.502	2496.802	0.975	29.978

Sample_ID	Household_ID	Country	Locality	Edible part	Species	Ca	Cu	Fe	Mg	Se	Zn
L-MO-40-RAM	40	Kenya	Ramogi	Leaf	MO	8540.18	5.136	236.037	2495.811	0.945	33.528
L-MO-41-RAM	41	Kenya	Ramogi	Leaf	MO	11929.95	8.673	391.262	4788.809	0.258	53.857
L-MO-41-RAM	41	Kenya	Ramogi	Leaf	MO	11683.003	8.486	390.24	4686.331	0.251	53.133
L-MO-41-RAM	41	Kenya	Ramogi	Leaf	MO	11743.022	8.612	368.521	4803.953	0.24	56.182
L-MO-42-RAM	42	Kenya	Ramogi	Leaf	MO	12357.403	7.775	415.031	5332.121	0.275	39.976
L-MO-42-RAM	42	Kenya	Ramogi	Leaf	MO	12180.555	7.756	376.518	5175.873	0.268	39.84
L-MO-42-RAM	42	Kenya	Ramogi	Leaf	MO	11559.187	7.747	336.588	4758.152	0.253	41.106
L-MO-43-RAM	43	Kenya	Ramogi	Leaf	MO	11946.515	7.414	1163.159	3451.372	0.153	21.694
L-MO-43-RAM	43	Kenya	Ramogi	Leaf	MO	12018.036	7.491	1191.06	3494.62	0.144	18.721
L-MO-43-RAM	43	Kenya	Ramogi	Leaf	MO	11174.245	7.146	1158.953	3277.931	0.14	16.666
L-MO-44-RAM	44	Kenya	Ramogi	Leaf	MO	11628.277	7.936	199.186	5962.548	0.033	24.612
L-MO-44-RAM	44	Kenya	Ramogi	Leaf	MO	11411.335	7.675	190.19	5820.412	0.032	24.439
L-MO-44-RAM	44	Kenya	Ramogi	Leaf	MO	11265.764	7.613	191.651	5776.307	0.029	23.555
L-MO-45-MAL	45	Kenya	Malindi	Leaf	MO	24245.928	8.034	159.647	5024.747	1.412	39.205
L-MO-45-MAL	45	Kenya	Malindi	Leaf	MO	21965.591	7.258	137.437	4572.19	1.379	35.458
L-MO-45-MAL	45	Kenya	Malindi	Leaf	MO	23248.971	7.769	148.741	4653.595	1.334	39.127
L-MO-46-MAL	46	Kenya	Malindi	Leaf	MO	15373.698	7.521	141.359	3733.575	0.796	42.145
L-MO-46-MAL	46	Kenya	Malindi	Leaf	MO	15241.794	7.414	143.69	3460.443	0.762	42.677
L-MO-46-MAL	46	Kenya	Malindi	Leaf	MO	15299.046	7.742	145.83	3454.804	0.762	43.197
L-MO-47-MAL	47	Kenya	Malindi	Leaf	MO	23969.371	8.282	204.89	6301.562	4.16	42.923
L-MO-47-MAL	47	Kenya	Malindi	Leaf	MO	22434.107	8.275	176.33	5887.946	3.804	45.015
L-MO-47-MAL	47	Kenya	Malindi	Leaf	MO	21356.205	7.756	164.2	5686.559	3.656	40.617
L-MO-48-MAL	48	Kenya	Malindi	Leaf	MO	16440.519	7.47	127.432	5824.229	2.239	35.279
L-MO-48-MAL	48	Kenya	Malindi	Leaf	MO	15253.828	7.128	121.522	5504.042	2.233	32.89
L-MO-48-MAL	48	Kenya	Malindi	Leaf	MO	16143.921	7.662	127.027	5723.155	2.218	35.554
L-MO-49-MAL	49	Kenya	Malindi	Leaf	MO	23040.086	4.905	239.187	3283.906	2.687	25.567
L-MO-49-MAL	49	Kenya	Malindi	Leaf	MO	21629.49	4.658	220.597	3279.483	2.62	23.552
L-MO-49-MAL	49	Kenya	Malindi	Leaf	MO	21798.865	4.835	223.082	3066.156	2.422	26.041
L-MO-4-KIB	4	Kenya	Kibwezi	Leaf	MO	20301.808	6.266	323.749	7574.741	4.178	55.966
L-MO-4-KIB	4	Kenya	Kibwezi	Leaf	MO	21120.693	6.539	332.692	7835.677	4.162	58.425
L-MO-4-KIB	4	Kenya	Kibwezi	Leaf	MO	21172.809	6.718	320.351	7738.175	4.061	57.888

Sample_ID	Household_ID	Country	Locality	Edible part	Species	Ca	Cu	Fe	Mg	Se	Zn
L-MO-50-MAL	50	Kenya	Malindi	Leaf	MO	12053.558	7.306	125.204	3258.178	1.014	33.083
L-MO-50-MAL	50	Kenya	Malindi	Leaf	MO	12298.212	7.637	129.746	3212.134	0.985	35.187
L-MO-50-MAL	50	Kenya	Malindi	Leaf	MO	11832.952	7.269	126.352	3097.351	0.933	32.897
L-MO-51-MAL	51	Kenya	Malindi	Leaf	MO	22145.524	8.065	118.331	5836.477	12.734	32.918
L-MO-51-MAL	51	Kenya	Malindi	Leaf	MO	20797.817	7.219	108.405	5523.445	12.459	29.162
L-MO-51-MAL	51	Kenya	Malindi	Leaf	MO	21783.366	7.507	112.684	5739.848	12.417	30.399
L-MO-52-MAL	52	Kenya	Malindi	Leaf	MO	18579.372	7.1	73.712	3219.619	2.65	34.832
L-MO-52-MAL	52	Kenya	Malindi	Leaf	MO	18504.765	7.481	75.723	3040.213	2.593	37.744
L-MO-52-MAL	52	Kenya	Malindi	Leaf	MO	18357.333	7.205	75.366	3023.73	2.556	36.273
L-MO-53-MAL	53	Kenya	Malindi	Leaf	MO	11176.777	8.678	92.353	5581.979	3.291	30.539
L-MO-53-MAL	53	Kenya	Malindi	Leaf	MO	10770.024	8.753	91.678	5298.165	3.034	30.42
L-MO-53-MAL	53	Kenya	Malindi	Leaf	MO	10826.645	8.615	93.745	5351.767	2.989	31.199
L-MO-54-MAL	54	Kenya	Malindi	Leaf	MO	11430.389	10.672	84.603	3160.936	1.447	32.591
L-MO-54-MAL	54	Kenya	Malindi	Leaf	MO	11065.215	10.006	78.096	3234.751	1.435	32.316
L-MO-54-MAL	54	Kenya	Malindi	Leaf	MO	11580.925	10.593	84.394	3222.011	1.429	33.94
L-MO-55-MAL	55	Kenya	Malindi	Leaf	MO	15826.143	3.053	102.435	5515.519	4.003	30.49
L-MO-55-MAL	55	Kenya	Malindi	Leaf	MO	15830.911	2.954	104.735	5495.125	3.883	31.704
L-MO-55-MAL	55	Kenya	Malindi	Leaf	MO	15267.267	3.01	99.907	5262.097	3.772	30.008
L-MO-56-UKU	56	Kenya	Ukunda	Leaf	MO	49042.497	7.048	125.051	7449.799	4.697	18.125
L-MO-56-UKU	56	Kenya	Ukunda	Leaf	MO	46154.569	6.876	120.508	6900.131	4.169	17.472
L-MO-56-UKU	56	Kenya	Ukunda	Leaf	MO	44576.629	6.435	116.867	6712.217	4.007	16.862
L-MO-57-UKU	57	Kenya	Ukunda	Leaf	MO	9888.276	5.847	98.573	4123.383	1.179	39.497
L-MO-57-UKU	57	Kenya	Ukunda	Leaf	MO	9221.689	5.435	92.817	4391.176	1.169	36.43
L-MO-57-UKU	57	Kenya	Ukunda	Leaf	MO	9752.287	5.991	97.648	4037.228	1.157	38.758
L-MO-58-UKU	58	Kenya	Ukunda	Leaf	MO	18963.579	3.236	218.765	5526.295	1.675	24.905
L-MO-58-UKU	58	Kenya	Ukunda	Leaf	MO	17417.472	3.055	193.314	5124.309	1.666	23.229
L-MO-58-UKU	58	Kenya	Ukunda	Leaf	MO	18096.447	3.31	236.412	5321.735	1.653	23.686
L-MO-58-UKU	58	Kenya	Ukunda	Leaf	MO	17900.779	3.619	237.415	5241.48	1.645	24.098
L-MO-58-UKU	58	Kenya	Ukunda	Leaf	MO	17859.238	3.274	210.156	5278.124	1.601	23.864
L-MO-59-UKU	59	Kenya	Ukunda	Leaf	MO	9993.203	4.864	117.742	3304.287	0.988	29.67
L-MO-59-UKU	59	Kenya	Ukunda	Leaf	MO	9627.644	4.969	116.638	3129.352	0.924	29.812

Sample_ID	Household_ID	Country	Locality	Edible part	Species	Ca	Cu	Fe	Mg	Se	Zn
L-MO-59-UKU	59	Kenya	Ukunda	Leaf	MO	9689.758	4.719	116.682	3145.916	0.919	30.004
L-MO-5-KIB	5	Kenya	Kibwezi	Leaf	MO	7443.766	14.39	127.442	5632.407	11.37	54.527
L-MO-5-KIB	5	Kenya	Kibwezi	Leaf	MO	6742.868	13.243	110.198	5210.831	11.093	49.797
L-MO-5-KIB	5	Kenya	Kibwezi	Leaf	MO	7115.254	14.067	119.224	5400.872	10.583	52.712
L-MO-60-UKU	60	Kenya	Ukunda	Leaf	MO	21859.247	6.284	72.707	3611.309	2.185	32.68
L-MO-60-UKU	60	Kenya	Ukunda	Leaf	MO	22163.387	5.888	73.175	3571.256	2.158	33.253
L-MO-60-UKU	60	Kenya	Ukunda	Leaf	MO	20798.021	5.576	67.666	3503.689	2.137	30.695
L-MO-61-UKU	61	Kenya	Ukunda	Leaf	MO	14299.987	6.464	399.733	4469.614	2.41	29.642
L-MO-61-UKU	61	Kenya	Ukunda	Leaf	MO	13543.05	6.246	343.096	3817.569	2.181	29.119
L-MO-61-UKU	61	Kenya	Ukunda	Leaf	MO	12895.073	6.145	321.891	3650.08	2.042	27.844
L-MO-62-UKU	62	Kenya	Ukunda	Leaf	MO	11846.729	3.379	316.688	4397.274	10.049	32.101
L-MO-62-UKU	62	Kenya	Ukunda	Leaf	MO	12395.285	3.267	296.17	4548.167	9.784	33.109
L-MO-62-UKU	62	Kenya	Ukunda	Leaf	MO	11526.998	3.462	280.297	3763.764	9.328	30.79
L-MO-6-KIB	6	Kenya	Kibwezi	Leaf	MO	12348.907	10.874	146.464	6204.793	5.098	70.263
L-MO-6-KIB	6	Kenya	Kibwezi	Leaf	MO	12444.778	11.088	141.265	6098.872	5.089	69.944
L-MO-6-KIB	6	Kenya	Kibwezi	Leaf	MO	11634.776	10.024	133.818	5857.41	5.033	64.252
L-MO-7-KIB	7	Kenya	Kibwezi	Leaf	MO	12000.354	9.735	178.72	5272.358	13.013	58.596
L-MO-7-KIB	7	Kenya	Kibwezi	Leaf	MO	12371.51	9.947	193.757	5355.475	12.485	60.743
L-MO-7-KIB	7	Kenya	Kibwezi	Leaf	MO	11475.265	9.414	174.646	5028.659	12.009	57.325
L-MO-8-KIB	8	Kenya	Kibwezi	Leaf	MO	13447.795	11.468	121.248	3566.233	4.471	31.227
L-MO-8-KIB	8	Kenya	Kibwezi	Leaf	MO	13503.855	11.531	113.783	3555.827	4.364	31.366
L-MO-8-KIB	8	Kenya	Kibwezi	Leaf	MO	11995.064	10.156	107.672	3407.194	4.268	26.683
L-MO-9-KIB	9	Kenya	Kibwezi	Leaf	MO	8379.978	7.514	186.038	4652.855	4.792	53.167
L-MO-9-KIB	9	Kenya	Kibwezi	Leaf	MO	8688.252	7.962	200.439	4202.889	4.758	54.973
L-MO-9-KIB	9	Kenya	Kibwezi	Leaf	MO	8594.58	7.603	195.34	4134.765	4.522	54.777
L-MS-31-BAR	31	Kenya	Baringo	Leaf	MS	22885.226	4.506	149.263	7017.441	7.745	21.021
L-MS-31-BAR	31	Kenya	Baringo	Leaf	MS	22486.014	4.378	146.952	6896.796	7.265	23.493
L-MS-31-BAR	31	Kenya	Baringo	Leaf	MS	22112.263	4.371	151.477	6760.564	7.179	21.488
L-MS-32-BAR	32	Kenya	Baringo	Leaf	MS	22608.058	3.002	137.13	7002.539	4.848	15.824
L-MS-32-BAR	32	Kenya	Baringo	Leaf	MS	23606.132	3.113	143.39	7276.266	4.781	19.07
L-MS-32-BAR	32	Kenya	Baringo	Leaf	MS	23781.074	3.084	149.783	7339.341	4.765	17.744

Sample_ID	Household_ID	Country	Locality	Edible part	Species	Ca	Cu	Fe	Mg	Se	Zn
L-MS-33-BAR	33	Kenya	Baringo	Leaf	MS	10325.215	1.778	193.818	6209.066	1.101	15.021
L-MS-33-BAR	33	Kenya	Baringo	Leaf	MS	9635.798	1.763	181.331	5850.921	1.098	12.721
L-MS-33-BAR	33	Kenya	Baringo	Leaf	MS	10213.116	1.736	194.626	6149.046	1.094	14.07
L-MS-34-BAR	34	Kenya	Baringo	Leaf	MS	21962.811	3.611	357.732	9731.933	1.699	12.658
L-MS-34-BAR	34	Kenya	Baringo	Leaf	MS	21278.872	3.468	318.179	9572.436	1.669	10.702
L-MS-34-BAR	34	Kenya	Baringo	Leaf	MS	21317.981	3.395	315.799	9466.901	1.62	11.129
L-MS-35-BAR	35	Kenya	Baringo	Leaf	MS	41803.31	3.197	226.22	16862.894	4.259	15.504
L-MS-35-BAR	35	Kenya	Baringo	Leaf	MS	41502.304	3.058	231.108	16609.86	3.903	15.583
L-MS-35-BAR	35	Kenya	Baringo	Leaf	MS	37653.587	2.847	213.583	15229.693	3.725	16.002
L-MS-37-RAM	37	Kenya	Ramogi	Leaf	MS	24671.76	4.639	247.094	6464.821	0.414	25.519
L-MS-37-RAM	37	Kenya	Ramogi	Leaf	MS	24422.363	4.484	233.543	6292.484	0.395	28.359
L-MS-37-RAM	37	Kenya	Ramogi	Leaf	MS	24716.864	4.402	240.793	6395.619	0.376	25.814
S-MO-10-KIB	10	Kenya	Kibwezi	Seed	MO	1088.838	6.188	61.136	3157.587	3.933	46.959
S-MO-10-KIB	10	Kenya	Kibwezi	Seed	MO	1092.824	6.16	65.18	3155.84	3.837	47.336
S-MO-10-KIB	10	Kenya	Kibwezi	Seed	MO	1121.764	6.37	65.776	3214.8	3.922	47.976
S-MO-11-KIB	11	Kenya	Kibwezi	Seed	MO	852.783	7.355	67.845	3089.534	2.601	41.318
S-MO-11-KIB	11	Kenya	Kibwezi	Seed	MO	891.237	5.125	67.625	3264.35	2.688	42.905
S-MO-11-KIB	11	Kenya	Kibwezi	Seed	MO	904.195	5.323	68.171	3771.881	2.902	44.433
S-MO-12-KIB	12	Kenya	Kibwezi	Seed	MO	888.161	5.338	54.468	2849.837	1.914	37.112
S-MO-12-KIB	12	Kenya	Kibwezi	Seed	MO	914.286	5.411	55.637	2940.267	2.017	39.104
S-MO-12-KIB	12	Kenya	Kibwezi	Seed	MO	935.027	5.623	55.041	3077.944	2.097	40.677
S-MO-13-KIB	13	Kenya	Kibwezi	Seed	MO	824.372	4.913	58.515	3259.724	1.681	50.728
S-MO-13-KIB	13	Kenya	Kibwezi	Seed	MO	837.409	4.88	59.971	3256.715	1.626	50.882
S-MO-13-KIB	13	Kenya	Kibwezi	Seed	MO	897.108	5.22	60.883	3902.186	1.79	54.555
S-MO-15-MBO	15	Kenya	Mbololo	Seed	MO	1805.367	3.506	41.366	3076.041	4.821	44.034
S-MO-15-MBO	15	Kenya	Mbololo	Seed	MO	1826.72	3.559	42.37	3136.601	4.925	46.165
S-MO-15-MBO	15	Kenya	Mbololo	Seed	MO	1898.786	3.827	43.465	3675.413	5.093	48.622
S-MO-16-MBO	16	Kenya	Mbololo	Seed	MO	1240.539	4.984	57.855	2822.81	1.869	56.694
S-MO-16-MBO	16	Kenya	Mbololo	Seed	MO	1244.152	4.826	55.733	2813.103	1.772	54.73
S-MO-16-MBO	16	Kenya	Mbololo	Seed	MO	1270.087	5.173	57.1	3135.528	1.926	59.686
S-MO-17-MBO	17	Kenya	Mbololo	Seed	MO	812.57	5.224	54.327	2991.172	0.744	56.017

Sample_ID	Household_ID	Country	Locality	Edible part	Species	Ca	Cu	Fe	Mg	Se	Zn
S-MO-17-MBO	17	Kenya	Mbololo	Seed	MO	845.224	5.526	54.516	3252.62	0.779	57.575
S-MO-17-MBO	17	Kenya	Mbololo	Seed	MO	872.544	5.449	56.843	3165.375	0.76	57.387
S-MO-18-MBO	18	Kenya	Mbololo	Seed	MO	923.881	4.145	57.109	3171.017	2.192	48.42
S-MO-18-MBO	18	Kenya	Mbololo	Seed	MO	926.461	4.432	54.867	3507.09	2.309	50.318
S-MO-18-MBO	18	Kenya	Mbololo	Seed	MO	953.732	4.388	58.64	3285.707	2.296	50.88
S-MO-19-MBO	19	Kenya	Mbololo	Seed	MO	1272.495	4.818	50.84	3579.533	4.078	55.443
S-MO-19-MBO	19	Kenya	Mbololo	Seed	MO	1492.37	3.297	38.787	2616.962	5.718	41.59
S-MO-19-MBO	19	Kenya	Mbololo	Seed	MO	1534.272	3.356	39.957	2635.168	5.562	40.834
S-MO-20-MBO	20	Kenya	Mbololo	Seed	MO	1146.108	4.174	47.284	2860.298	3.635	48.705
S-MO-20-MBO	20	Kenya	Mbololo	Seed	MO	1188.479	4.381	48.381	2946.064	3.721	49.493
S-MO-20-MBO	20	Kenya	Mbololo	Seed	MO	1606.664	3.743	41.633	2830.163	6.147	44.583
S-MO-21-MBO	21	Kenya	Mbololo	Seed	MO	1089.631	5.847	41.648	3184.864	4.455	39.504
S-MO-21-MBO	21	Kenya	Mbololo	Seed	MO	1098.564	5.728	42.92	3017.345	4.307	38.166
S-MO-21-MBO	21	Kenya	Mbololo	Seed	MO	1114.303	5.925	43.034	3082.746	4.559	40.495
S-MO-22-MBO	22	Kenya	Mbololo	Seed	MO	1156.831	4.939	54.817	2815.599	2.808	56.795
S-MO-22-MBO	22	Kenya	Mbololo	Seed	MO	1321.17	5.573	61.878	3146.605	3.057	63.284
S-MO-22-MBO	22	Kenya	Mbololo	Seed	MO	1356.367	5.795	63.139	3322.903	3.229	66.587
S-MO-23-MBO	23	Kenya	Mbololo	Seed	MO	1156.44	3.718	46.853	2803.604	4.711	41.365
S-MO-23-MBO	23	Kenya	Mbololo	Seed	MO	1202.759	3.911	49.03	2930.38	4.815	43.598
S-MO-23-MBO	23	Kenya	Mbololo	Seed	MO	1284.776	4.293	50.794	3178.404	5.211	47.877
S-MO-24-MBO	24	Kenya	Mbololo	Seed	MO	1442.278	2.599	42.117	2272.395	1.211	40.085
S-MO-24-MBO	24	Kenya	Mbololo	Seed	MO	1744.165	2.92	51.766	2658.615	1.324	44.718
S-MO-24-MBO	24	Kenya	Mbololo	Seed	MO	1760.342	2.972	54.042	2753.182	1.367	46.169
S-MO-25-MBO	25	Kenya	Mbololo	Seed	MO	1300.922	2.455	37.799	2669.703	2.936	36.994
S-MO-25-MBO	25	Kenya	Mbololo	Seed	MO	1561.054	2.723	45.629	2906.662	3.528	42.814
S-MO-25-MBO	25	Kenya	Mbololo	Seed	MO	1735.591	3.047	51.684	3258.405	3.757	48.279
S-MO-26-MBO	26	Kenya	Mbololo	Seed	MO	1397.939	4.935	48.828	3330.743	0.759	41.887
S-MO-26-MBO	26	Kenya	Mbololo	Seed	MO	1426.134	5.089	50.008	3831.824	0.782	45.242
S-MO-26-MBO	26	Kenya	Mbololo	Seed	MO	1434.565	5.057	51.255	3588.895	0.789	43.929
S-MO-27-MBO	27	Kenya	Mbololo	Seed	MO	1803.65	5.032	45.431	3442.325	2.246	40.959
S-MO-27-MBO	27	Kenya	Mbololo	Seed	MO	1816.748	4.896	44.346	3021.65	2.156	40.043

Sample_ID	Household_ID	Country	Locality	Edible part	Species	Ca	Cu	Fe	Mg	Se	Zn
S-MO-27-MBO	27	Kenya	Mbololo	Seed	MO	1865.786	5.045	47.579	3135.673	2.243	41.79
S-MO-28-MBO	28	Kenya	Mbololo	Seed	MO	1861.324	3.621	34.827	3017.235	3.102	35.108
S-MO-28-MBO	28	Kenya	Mbololo	Seed	MO	1872.384	3.884	34.729	3246.818	3.28	36.105
S-MO-28-MBO	28	Kenya	Mbololo	Seed	MO	1902.664	3.732	35.158	3084.739	3.197	36.021
S-MO-29-MBO	29	Kenya	Mbololo	Seed	MO	1171.323	4.378	53.564	3065.863	10.767	49.976
S-MO-29-MBO	29	Kenya	Mbololo	Seed	MO	1221.602	4.608	56.566	3216.686	11.145	52.516
S-MO-29-MBO	29	Kenya	Mbololo	Seed	MO	1243.07	4.748	54.42	3716.001	11.574	54.054
S-MO-2-KIB	2	Kenya	Kibwezi	Seed	MO	1464.857	4.638	48.967	2817.894	7.356	48.927
S-MO-2-KIB	2	Kenya	Kibwezi	Seed	MO	1477.417	4.756	50.376	2889.157	7.758	51.129
S-MO-2-KIB	2	Kenya	Kibwezi	Seed	MO	1489.08	4.613	47.871	2869.587	7.645	51.057
S-MO-30-MBO	30	Kenya	Mbololo	Seed	MO	2002.712	3.132	49.401	2503.683	3.352	44.886
S-MO-30-MBO	30	Kenya	Mbololo	Seed	MO	2121.303	3.613	52.667	2762.516	3.737	49.62
S-MO-30-MBO	30	Kenya	Mbololo	Seed	MO	2207.857	3.548	55.298	2796.186	3.699	50.176
S-MO-37-RAM	37	Kenya	Ramogi	Seed	MO	1652.12	3.043	39.414	2772.895	0.25	36.09
S-MO-37-RAM	37	Kenya	Ramogi	Seed	MO	1707.006	3.152	40.818	2811.262	0.254	36.937
S-MO-37-RAM	37	Kenya	Ramogi	Seed	MO	1736.055	3.397	41.979	3064.248	0.266	38.659
S-MO-38-RAM	38	Kenya	Ramogi	Seed	MO	1307.803	3.216	44.893	3740.538	2.536	48.634
S-MO-38-RAM	38	Kenya	Ramogi	Seed	MO	1311.611	3.211	44.316	4153.014	2.488	48.026
S-MO-38-RAM	38	Kenya	Ramogi	Seed	MO	1329.381	3.493	44.585	4211.991	2.602	49.915
S-MO-39-RAM	39	Kenya	Ramogi	Seed	MO	1126.811	3.274	48.511	3391.673	0.501	42.67
S-MO-39-RAM	39	Kenya	Ramogi	Seed	MO	1147.103	3.494	51	3681.416	0.529	43.914
S-MO-39-RAM	39	Kenya	Ramogi	Seed	MO	1170.267	3.663	49.507	4013.866	0.523	43.44
S-MO-3-KIB	3	Kenya	Kibwezi	Seed	MO	997.447	4.42	50.051	3036.316	3.42	51.864
S-MO-3-KIB	3	Kenya	Kibwezi	Seed	MO	1000.644	4.438	53.602	3029.94	3.361	53.474
S-MO-3-KIB	3	Kenya	Kibwezi	Seed	MO	1087.195	4.876	56.892	3272.191	3.551	54.943
S-MO-40-RAM	40	Kenya	Ramogi	Seed	MO	1233.573	2.736	43.347	2918.271	1.001	43.026
S-MO-40-RAM	40	Kenya	Ramogi	Seed	MO	1253.71	2.773	43.698	2931.093	0.99	42.977
S-MO-40-RAM	40	Kenya	Ramogi	Seed	MO	1276.426	2.998	42.75	3026.627	1.041	42.896
S-MO-41-RAM	41	Kenya	Ramogi	Seed	MO	1033.087	2.698	54.131	3030.134	0.206	45.348
S-MO-41-RAM	41	Kenya	Ramogi	Seed	MO	1067.521	2.878	57.007	3154.734	0.213	47.249
S-MO-41-RAM	41	Kenya	Ramogi	Seed	MO	1071.142	2.889	55.98	3204.202	0.199	46.586

Sample_ID	Household_ID	Country	Locality	Edible part	Species	Ca	Cu	Fe	Mg	Se	Zn
S-MO-41-RAM	41	Kenya	Ramogi	Seed	MO	1097.592	3.105	57.393	3676.595	0.226	48.165
S-MO-42-RAM	42	Kenya	Ramogi	Seed	MO	760.449	2.755	42.458	3103.747	0.332	45.234
S-MO-42-RAM	42	Kenya	Ramogi	Seed	MO	764.119	2.628	43.032	3139.964	0.322	45.625
S-MO-42-RAM	42	Kenya	Ramogi	Seed	MO	783.035	2.875	44.02	3684.23	0.368	47.363
S-MO-44-RAM	44	Kenya	Ramogi	Seed	MO	1292.997	3.882	51.72	2818.5	0.013	51.915
S-MO-44-RAM	44	Kenya	Ramogi	Seed	MO	1329.008	4.17	52.517	3202.649	0.016	54.413
S-MO-44-RAM	44	Kenya	Ramogi	Seed	MO	1330.476	3.957	54.321	2895.544	0.016	54.187
S-MO-45-MAL	45	Kenya	Malindi	Seed	MO	1769.485	9.026	63.372	3138.098	1.284	80.626
S-MO-45-MAL	45	Kenya	Malindi	Seed	MO	1774.111	9.413	64.963	3190.673	1.296	81.459
S-MO-45-MAL	45	Kenya	Malindi	Seed	MO	1858.288	10.02	66.237	3798.166	1.406	89.332
S-MO-4-KIB	4	Kenya	Kibwezi	Seed	MO	773.008	3.38	47.36	3405.61	1.558	42.432
S-MO-4-KIB	4	Kenya	Kibwezi	Seed	MO	798.121	3.414	51.024	3219.838	1.554	43.381
S-MO-4-KIB	4	Kenya	Kibwezi	Seed	MO	821.719	3.413	52.817	3340.169	1.693	44.722
S-MO-52-MAL	52	Kenya	Malindi	Seed	MO	1388.098	4.351	44.122	2003.411	5.282	41.078
S-MO-52-MAL	52	Kenya	Malindi	Seed	MO	1421.216	4.587	44.014	2120.884	5.541	42.356
S-MO-52-MAL	52	Kenya	Malindi	Seed	MO	1471.008	4.541	47.351	2111.443	5.508	43.963
S-MO-5-KIB	5	Kenya	Kibwezi	Seed	MO	1165.386	3.69	42.478	2348.48	4.916	38.445
S-MO-5-KIB	5	Kenya	Kibwezi	Seed	MO	1201.166	3.652	45.16	2314.132	4.781	38.605
S-MO-5-KIB	5	Kenya	Kibwezi	Seed	MO	1206.481	3.779	44.038	2333.402	4.785	37.839
S-MO-60-UKU	60	Kenya	Ukunda	Seed	MO	1646.564	2.541	57.57	3209.191	2.591	53
S-MO-60-UKU	60	Kenya	Ukunda	Seed	MO	1652.116	2.566	57.029	3214.483	2.595	53.232
S-MO-60-UKU	60	Kenya	Ukunda	Seed	MO	1719.219	2.824	57.904	3673.005	2.76	53.333
S-MO-6-KIB	6	Kenya	Kibwezi	Seed	MO	1530.934	4.011	48.489	2372.412	3.976	37.938
S-MO-6-KIB	6	Kenya	Kibwezi	Seed	MO	1555.53	4.104	49.857	2499.936	4.233	40.37
S-MO-6-KIB	6	Kenya	Kibwezi	Seed	MO	1586.747	4.021	51.393	2463.693	4.182	40.311
