# Supplementary material for: Variation in the mineral element concentration of Moringa oleifera Lam. and M. stenopetala (Bak. f.) Cuf.: Role in human nutrition
Source: PLoS One. 2017 Apr 7;12(4):e0175503. doi: 10.1371/journal.pone.0175503 (PMC5384779; doi:10.1371/journal.pone.0175503)
Supplement: S35 Table — (PDF) [file pone.0175503.s035.pdf]

S35 Table. Raw data on MO and MS leaves iodine concentration(mg kg^{-1}) and sample details.

Sample_ID	Household_ID	Country	Locality	Edible part	Species	Iodine
ETF0001	KaratKonso	Ethiopia	Konso	Leaf	MS	0.00805
ETF0001	KaratKonso	Ethiopia	Konso	Leaf	MS	0.01410
ETF0002	KaratKonso	Ethiopia	Konso	Leaf	MS	0.20917
ETF0002	KaratKonso	Ethiopia	Konso	Leaf	MS	0.23210
ETF0005	ETH001	Ethiopia	Derashe	Leaf	MS	0.01086
ETF0005	ETH001	Ethiopia	Derashe	Leaf	MS	0.01122
ETF0009	ETH002	Ethiopia	Derashe	Leaf	MS	0.02928
ETF0009	ETH002	Ethiopia	Derashe	Leaf	MS	0.02960
ETF0012	ETH003	Ethiopia	Derashe	Leaf	MS	0.02501
ETF0012	ETH003	Ethiopia	Derashe	Leaf	MS	0.03169
ETF0015	ETH006	Ethiopia	Derashe	Leaf	MS	0.00064
ETF0016	ETH007	Ethiopia	Derashe	Leaf	MS	0.06084
ETF0016	ETH007	Ethiopia	Derashe	Leaf	MS	0.06573
ETF0019	ETH009	Ethiopia	Derashe	Leaf	MS	0.02092
ETF0019	ETH009	Ethiopia	Derashe	Leaf	MS	0.04226
ETF0022	ETH010	Ethiopia	Derashe	Leaf	MS	0.05096
ETF0022	ETH010	Ethiopia	Derashe	Leaf	MS	0.05339
ETF0025	ETH011	Ethiopia	Derashe	Leaf	MS	0.01330
ETF0025	ETH011	Ethiopia	Derashe	Leaf	MS	0.01966
ETF0026	ETH012	Ethiopia	Derashe	Leaf	MS	0.02916
ETF0026	ETH012	Ethiopia	Derashe	Leaf	MS	0.03324
ETF0036	ETH016	Ethiopia	Konso	Leaf	MS	0.00880
ETF0042	ETH018	Ethiopia	Konso	Leaf	MS	0.00074

Sample_ID	Household_ID	Country	Locality	Edible part	Species	Iodine
ETF0042	ETH018	Ethiopia	Konso	Leaf	MS	0.00111
ETF0045	ETH019	Ethiopia	Konso	Leaf	MS	0.05797
ETF0045	ETH019	Ethiopia	Konso	Leaf	MS	0.06988
ETF0048	ETH020	Ethiopia	Konso	Leaf	MS	0.07041
ETF0048	ETH020	Ethiopia	Konso	Leaf	MS	0.07981
ETF0051	ETH021	Ethiopia	Konso	Leaf	MS	0.02388
ETF0051	ETH021	Ethiopia	Konso	Leaf	MS	0.02660
ETF0053	ETH022	Ethiopia	Konso	Leaf	MS	0.02257
ETF0053	ETH022	Ethiopia	Konso	Leaf	MS	0.02299
ETF0057	SeGen	Ethiopia	Konso	Leaf	MS	0.03353
ETF0057	SeGen	Ethiopia	Konso	Leaf	MS	0.03866
ETF0058	SeGen	Ethiopia	Konso	Leaf	MS	0.03635
ETF0058	SeGen	Ethiopia	Konso	Leaf	MS	0.04360
Eth-Haw-1	Eth-Haw-1	Ethiopia	Hawasa	Leaf	MS	0.08229
Eth-Haw-1	Eth-Haw-1	Ethiopia	Hawasa	Leaf	MS	0.10765
Eth-Haw-10	Eth-Haw-10	Ethiopia	Hawasa	Leaf	MS	0.06448
Eth-Haw-10	Eth-Haw-10	Ethiopia	Hawasa	Leaf	MS	0.07080
Eth-Haw-11	Eth-Haw-11	Ethiopia	Hawasa	Leaf	MS	0.03492
Eth-Haw-11	Eth-Haw-11	Ethiopia	Hawasa	Leaf	MS	0.04624
Eth-Haw-12	Eth-Haw-12	Ethiopia	Hawasa	Leaf	MS	0.03329
Eth-Haw-12	Eth-Haw-12	Ethiopia	Hawasa	Leaf	MS	0.03380
Eth-Haw-13	Eth-Haw-13	Ethiopia	Hawasa	Leaf	MS	0.02743
Eth-Haw-13	Eth-Haw-13	Ethiopia	Hawasa	Leaf	MS	0.03043
Eth-Haw-14	Eth-Haw-14	Ethiopia	Hawasa	Leaf	MS	0.10493
Eth-Haw-14	Eth-Haw-14	Ethiopia	Hawasa	Leaf	MS	0.11260

Sample_ID	Household_ID	Country	Locality	Edible part	Species	Iodine
Eth-Haw-2	Eth-Haw-2	Ethiopia	Hawasa	Leaf	MS	0.06470
Eth-Haw-2	Eth-Haw-2	Ethiopia	Hawasa	Leaf	MS	0.06487
Eth-Haw-3	Eth-Haw-3	Ethiopia	Hawasa	Leaf	MS	0.02251
Eth-Haw-3	Eth-Haw-3	Ethiopia	Hawasa	Leaf	MS	0.03089
Eth-Haw-4	Eth-Haw-4	Ethiopia	Hawasa	Leaf	MS	0.05237
Eth-Haw-4	Eth-Haw-4	Ethiopia	Hawasa	Leaf	MS	0.06177
Eth-Haw-5	Eth-Haw-5	Ethiopia	Hawasa	Leaf	MS	0.00403
Eth-Haw-5	Eth-Haw-5	Ethiopia	Hawasa	Leaf	MS	0.00604
Eth-Haw-6	Eth-Haw-6	Ethiopia	Hawasa	Leaf	MS	0.00376
Eth-Haw-6	Eth-Haw-6	Ethiopia	Hawasa	Leaf	MS	0.00480
Eth-Haw-7	Eth-Haw-7	Ethiopia	Hawasa	Leaf	MS	0.01722
Eth-Haw-7	Eth-Haw-7	Ethiopia	Hawasa	Leaf	MS	0.02472
Eth-Haw-8	Eth-Haw-8	Ethiopia	Hawasa	Leaf	MS	0.06392
Eth-Haw-8	Eth-Haw-8	Ethiopia	Hawasa	Leaf	MS	0.06877
Eth-Haw-9	Eth-Haw-9	Ethiopia	Hawasa	Leaf	MS	0.08967
Eth-Haw-9	Eth-Haw-9	Ethiopia	Hawasa	Leaf	MS	0.09389
L-MO-10-KIB	10	Kenya	Kibwezi	Leaf	MO	0.24785
L-MO-10-KIB	10	Kenya	Kibwezi	Leaf	MO	0.27216
L-MO-11-KIB	11	Kenya	Kibwezi	Leaf	MO	0.13559
L-MO-11-KIB	11	Kenya	Kibwezi	Leaf	MO	0.14249
L-MO-12-KIB	12	Kenya	Kibwezi	Leaf	MO	0.08083
L-MO-12-KIB	12	Kenya	Kibwezi	Leaf	MO	0.10175
L-MO-13-KIB	13	Kenya	Kibwezi	Leaf	MO	0.16351
L-MO-13-KIB	13	Kenya	Kibwezi	Leaf	MO	0.18150
L-MO-14-KIB	14	Kenya	Kibwezi	Leaf	MO	0.20803

Sample_ID	Household_ID	Country	Locality	Edible part	Species	Iodine
L-MO-14-KIB	14	Kenya	Kibwezi	Leaf	MO	0.23187
L-MO-15-MBO	15	Kenya	Mbololo	Leaf	MO	0.53443
L-MO-15-MBO	15	Kenya	Mbololo	Leaf	MO	0.55524
L-MO-16-MBO	16	Kenya	Mbololo	Leaf	MO	0.14219
L-MO-16-MBO	16	Kenya	Mbololo	Leaf	MO	0.14284
L-MO-17-MBO	17	Kenya	Mbololo	Leaf	MO	0.34624
L-MO-17-MBO	17	Kenya	Mbololo	Leaf	MO	0.35470
L-MO-18-MBO	18	Kenya	Mbololo	Leaf	MO	0.36513
L-MO-18-MBO	18	Kenya	Mbololo	Leaf	MO	0.36666
L-MO-19-MBO	19	Kenya	Mbololo	Leaf	MO	0.41365
L-MO-19-MBO	19	Kenya	Mbololo	Leaf	MO	0.42010
L-MO-1-KIB	1	Kenya	Kibwezi	Leaf	MO	0.06405
L-MO-1-KIB	1	Kenya	Kibwezi	Leaf	MO	0.07346
L-MO-20-MBO	20	Kenya	Mbololo	Leaf	MO	0.26065
L-MO-20-MBO	20	Kenya	Mbololo	Leaf	MO	0.26303
L-MO-21-MBO	21	Kenya	Mbololo	Leaf	MO	0.39073
L-MO-21-MBO	21	Kenya	Mbololo	Leaf	MO	0.40413
L-MO-22-MBO	22	Kenya	Mbololo	Leaf	MO	0.26047
L-MO-22-MBO	22	Kenya	Mbololo	Leaf	MO	0.26771
L-MO-23-MBO	23	Kenya	Mbololo	Leaf	MO	0.26169
L-MO-23-MBO	23	Kenya	Mbololo	Leaf	MO	0.28131
L-MO-24-MBO	24	Kenya	Mbololo	Leaf	MO	0.39738
L-MO-24-MBO	24	Kenya	Mbololo	Leaf	MO	0.40247
L-MO-25-MBO	25	Kenya	Mbololo	Leaf	MO	0.18750
L-MO-25-MBO	25	Kenya	Mbololo	Leaf	MO	0.19716

Sample_ID	Household_ID	Country	Locality	Edible part	Species	Iodine
L-MO-26-MBO	26	Kenya	Mbololo	Leaf	MO	0.27306
L-MO-26-MBO	26	Kenya	Mbololo	Leaf	MO	0.28856
L-MO-27-MBO	27	Kenya	Mbololo	Leaf	MO	0.41908
L-MO-27-MBO	27	Kenya	Mbololo	Leaf	MO	0.42219
L-MO-28-MBO	28	Kenya	Mbololo	Leaf	MO	0.37786
L-MO-28-MBO	28	Kenya	Mbololo	Leaf	MO	0.40110
L-MO-29-MBO	29	Kenya	Mbololo	Leaf	MO	0.39615
L-MO-29-MBO	29	Kenya	Mbololo	Leaf	MO	0.40961
L-MO-2-KIB	2	Kenya	Kibwezi	Leaf	MO	0.12812
L-MO-2-KIB	2	Kenya	Kibwezi	Leaf	MO	0.20794
L-MO-30-MBO	30	Kenya	Mbololo	Leaf	MO	0.30560
L-MO-30-MBO	30	Kenya	Mbololo	Leaf	MO	0.30750
L-MO-37-RAM	37	Kenya	Ramogi	Leaf	MO	0.03611
L-MO-37-RAM	37	Kenya	Ramogi	Leaf	MO	0.04136
L-MO-38-RAM	38	Kenya	Ramogi	Leaf	MO	0.00051
L-MO-39-RAM	39	Kenya	Ramogi	Leaf	MO	0.02747
L-MO-39-RAM	39	Kenya	Ramogi	Leaf	MO	0.03787
L-MO-3-KIB	3	Kenya	Kibwezi	Leaf	MO	0.29953
L-MO-3-KIB	3	Kenya	Kibwezi	Leaf	MO	0.31595
L-MO-40-RAM	40	Kenya	Ramogi	Leaf	MO	0.01306
L-MO-40-RAM	40	Kenya	Ramogi	Leaf	MO	0.01840
L-MO-41-RAM	41	Kenya	Ramogi	Leaf	MO	0.08942
L-MO-41-RAM	41	Kenya	Ramogi	Leaf	MO	0.09632
L-MO-42-RAM	42	Kenya	Ramogi	Leaf	MO	0.11201
L-MO-42-RAM	42	Kenya	Ramogi	Leaf	MO	0.11321

Sample_ID	Household_ID	Country	Locality	Edible part	Species	Iodine
L-MO-43-RAM	43	Kenya	Ramogi	Leaf	MO	0.20202
L-MO-43-RAM	43	Kenya	Ramogi	Leaf	MO	0.20612
L-MO-44-RAM	44	Kenya	Ramogi	Leaf	MO	0.03497
L-MO-44-RAM	44	Kenya	Ramogi	Leaf	MO	0.03697
L-MO-45-MAL	45	Kenya	Malindi	Leaf	MO	0.26393
L-MO-45-MAL	45	Kenya	Malindi	Leaf	MO	0.28577
L-MO-46-MAL	46	Kenya	Malindi	Leaf	MO	0.28825
L-MO-46-MAL	46	Kenya	Malindi	Leaf	MO	0.29255
L-MO-47-MAL	47	Kenya	Malindi	Leaf	MO	0.13250
L-MO-47-MAL	47	Kenya	Malindi	Leaf	MO	0.13781
L-MO-48-MAL	48	Kenya	Malindi	Leaf	MO	0.27251
L-MO-48-MAL	48	Kenya	Malindi	Leaf	MO	0.27886
L-MO-49-MAL	49	Kenya	Malindi	Leaf	MO	0.35482
L-MO-49-MAL	49	Kenya	Malindi	Leaf	MO	0.37624
L-MO-4-KIB	4	Kenya	Kibwezi	Leaf	MO	0.13883
L-MO-4-KIB	4	Kenya	Kibwezi	Leaf	MO	0.19260
L-MO-50-MAL	50	Kenya	Malindi	Leaf	MO	0.22113
L-MO-50-MAL	50	Kenya	Malindi	Leaf	MO	0.23907
L-MO-51-MAL	51	Kenya	Malindi	Leaf	MO	0.28819
L-MO-51-MAL	51	Kenya	Malindi	Leaf	MO	0.30157
L-MO-52-MAL	52	Kenya	Malindi	Leaf	MO	0.24702
L-MO-52-MAL	52	Kenya	Malindi	Leaf	MO	0.26086
L-MO-53-MAL	53	Kenya	Malindi	Leaf	MO	0.19479
L-MO-53-MAL	53	Kenya	Malindi	Leaf	MO	0.19933
L-MO-54-MAL	54	Kenya	Malindi	Leaf	MO	0.21324

Sample_ID	Household_ID	Country	Locality	Edible part	Species	Iodine
L-MO-54-MAL	54	Kenya	Malindi	Leaf	MO	0.22622
L-MO-55-MAL	55	Kenya	Malindi	Leaf	MO	0.17374
L-MO-55-MAL	55	Kenya	Malindi	Leaf	MO	0.17641
L-MO-56-UKU	56	Kenya	Ukunda	Leaf	MO	0.46125
L-MO-56-UKU	56	Kenya	Ukunda	Leaf	MO	0.49027
L-MO-57-UKU	57	Kenya	Ukunda	Leaf	MO	0.12914
L-MO-57-UKU	57	Kenya	Ukunda	Leaf	MO	0.13056
L-MO-58-UKU	58	Kenya	Ukunda	Leaf	MO	0.38346
L-MO-58-UKU	58	Kenya	Ukunda	Leaf	MO	0.38441
L-MO-59-UKU	59	Kenya	Ukunda	Leaf	MO	0.13451
L-MO-59-UKU	59	Kenya	Ukunda	Leaf	MO	0.13460
L-MO-5-KIB	5	Kenya	Kibwezi	Leaf	MO	0.09179
L-MO-5-KIB	5	Kenya	Kibwezi	Leaf	MO	0.09306
L-MO-60-UKU	60	Kenya	Ukunda	Leaf	MO	0.05627
L-MO-60-UKU	60	Kenya	Ukunda	Leaf	MO	0.05666
L-MO-61-UKU	61	Kenya	Ukunda	Leaf	MO	0.13129
L-MO-61-UKU	61	Kenya	Ukunda	Leaf	MO	0.13270
L-MO-62-UKU	62	Kenya	Ukunda	Leaf	MO	0.10009
L-MO-62-UKU	62	Kenya	Ukunda	Leaf	MO	0.11145
L-MO-6-KIB	6	Kenya	Kibwezi	Leaf	MO	0.09125
L-MO-6-KIB	6	Kenya	Kibwezi	Leaf	MO	0.09466
L-MO-7-KIB	7	Kenya	Kibwezi	Leaf	MO	0.10353
L-MO-7-KIB	7	Kenya	Kibwezi	Leaf	MO	0.10928
L-MO-8-KIB	8	Kenya	Kibwezi	Leaf	MO	0.07203
L-MO-8-KIB	8	Kenya	Kibwezi	Leaf	MO	0.08373

Sample_ID	Household_ID	Country	Locality	Edible part	Species	Iodine
L-MO-9-KIB	9	Kenya	Kibwezi	Leaf	MO	0.16253
L-MO-9-KIB	9	Kenya	Kibwezi	Leaf	MO	0.16791
L-MS-31-BAR	31	Kenya	Baringo	Leaf	MS	0.16760
L-MS-31-BAR	31	Kenya	Baringo	Leaf	MS	0.17085
L-MS-32-BAR	32	Kenya	Baringo	Leaf	MS	0.31108
L-MS-32-BAR	32	Kenya	Baringo	Leaf	MS	0.31197
L-MS-33-BAR	33	Kenya	Baringo	Leaf	MS	0.23818
L-MS-33-BAR	33	Kenya	Baringo	Leaf	MS	0.24822
L-MS-34-BAR	34	Kenya	Baringo	Leaf	MS	0.18610
L-MS-34-BAR	34	Kenya	Baringo	Leaf	MS	0.19281
L-MS-35-BAR	35	Kenya	Baringo	Leaf	MS	0.26194
L-MS-35-BAR	35	Kenya	Baringo	Leaf	MS	0.27920
