# Supplementary material for: Variation in the mineral element concentration of Moringa oleifera Lam. and M. stenopetala (Bak. f.) Cuf.: Role in human nutrition
Source: PLoS One. 2017 Apr 7;12(4):e0175503. doi: 10.1371/journal.pone.0175503 (PMC5384779; doi:10.1371/journal.pone.0175503)
Supplement: S36 Table — ND = not detectable. (PDF) [file pone.0175503.s036.pdf]

S36 Table. Raw data on elemental concentrations (mg kg^{-1}) in various crops and sample details. ND = not detectable.

Sample_ID	Household_ID	Country	Locality	Sample_Type	Tissue	Category	Ca	Cu	Fe	Mg	Se	Zn
Sorg-37-RAM	37	Kenya	Ramogi	Plant	Grain	Sorghum	145.120	1.506	36.011	1143.848	0.018	23.618
Sorg-37-RAM	37	Kenya	Ramogi	Plant	Grain	Sorghum	155.627	12.997	39.317	1217.202	0.023	25.973
Sorg-37-RAM	37	Kenya	Ramogi	Plant	Grain	Sorghum	162.358	1.899	41.979	1307.427	0.023	26.429
Sorg-38-RAM	38	Kenya	Ramogi	Plant	Grain	Sorghum	134.240	3.936	27.856	1125.570	0.339	17.066
Sorg-38-RAM	38	Kenya	Ramogi	Plant	Grain	Sorghum	136.626	0.777	26.708	1055.488	0.337	15.415
Sorg-38-RAM	38	Kenya	Ramogi	Plant	Grain	Sorghum	141.623	12.549	30.473	1178.028	0.398	18.264
Sorg-40-RAM	40	Kenya	Ramogi	Plant	Grain	Sorghum	102.851	5.714	30.918	1325.646	0.699	22.417
Sorg-40-RAM	40	Kenya	Ramogi	Plant	Grain	Sorghum	109.898	2.463	31.749	1323.323	0.722	22.470
Sorg-40-RAM	40	Kenya	Ramogi	Plant	Grain	Sorghum	112.026	13.800	35.305	1477.339	0.771	26.163
Sorg-41-RAM	41	Kenya	Ramogi	Plant	Grain	Sorghum	87.086	3.748	30.029	1315.816	0.043	16.728
Sorg-41-RAM	41	Kenya	Ramogi	Plant	Grain	Sorghum	91.277	0.018	28.913	1229.417	0.046	15.753
Sorg-41-RAM	41	Kenya	Ramogi	Plant	Grain	Sorghum	98.284	13.907	34.781	1481.531	0.052	19.335
Sorg-42-RAM	42	Kenya	Ramogi	Plant	Grain	Sorghum	72.598	ND	30.404	1061.981	0.004	18.482
Sorg-42-RAM	42	Kenya	Ramogi	Plant	Grain	Sorghum	80.847	4.356	32.645	1172.124	0.003	20.805
Sorg-42-RAM	42	Kenya	Ramogi	Plant	Grain	Sorghum	90.967	15.487	40.904	1435.810	0.010	25.365
Sorg-43-RAM	43	Kenya	Ramogi	Plant	Grain	Sorghum	93.860	1.328	44.586	1116.175	0.021	24.369
Sorg-43-RAM	43	Kenya	Ramogi	Plant	Grain	Sorghum	94.816	7.781	44.781	1139.437	0.014	25.471
Sorg-43-RAM	43	Kenya	Ramogi	Plant	Grain	Sorghum	105.035	15.298	54.135	1350.724	0.026	30.944
Maize-37-RAM	37	Kenya	Ramogi	Plant	Grain	WhiteMaize	43.341	8.710	18.378	811.298	0.030	20.034
Maize-37-RAM	37	Kenya	Ramogi	Plant	Grain	WhiteMaize	49.765	0.199	18.567	769.193	0.031	18.445
Maize-37-RAM	37	Kenya	Ramogi	Plant	Grain	WhiteMaize	60.384	16.044	20.029	860.076	0.040	21.211
Maize-38-RAM	38	Kenya	Ramogi	Plant	Grain	YellowMaize	39.182	2.464	14.760	819.421	0.023	20.140
Maize-38-RAM	38	Kenya	Ramogi	Plant	Grain	YellowMaize	49.475	0.586	15.536	840.369	0.025	20.613
Maize-38-RAM	38	Kenya	Ramogi	Plant	Grain	YellowMaize	54.868	17.557	17.752	922.070	0.034	23.520
Maize-39-RAM	39	Kenya	Ramogi	Plant	Grain	YellowMaize	33.567	2.769	16.198	947.484	0.214	29.641
Maize-39-RAM	39	Kenya	Ramogi	Plant	Grain	YellowMaize	37.182	ND	18.807	1103.687	0.214	32.843
Maize-39-RAM	39	Kenya	Ramogi	Plant	Grain	YellowMaize	51.576	18.001	20.603	1175.442	0.244	35.678
Maize-40-RAM	40	Kenya	Ramogi	Plant	Grain	MixedYellowWhiteMaize	25.737	2.875	16.280	776.514	0.046	24.305
Maize-40-RAM	40	Kenya	Ramogi	Plant	Grain	MixedYellowWhiteMaize	32.448	ND	22.289	1020.128	0.051	30.863
Maize-40-RAM	40	Kenya	Ramogi	Plant	Grain	MixedYellowWhiteMaize	46.043	2.877	20.813	1038.633	0.065	31.619
Maize-41-RAM	41	Kenya	Ramogi	Plant	Grain	MixedYellowWhiteMaize	44.478	3.914	15.950	818.351	ND	19.456
Maize-41-RAM	41	Kenya	Ramogi	Plant	Grain	MixedYellowWhiteMaize	48.740	0.360	16.968	857.347	0.000	19.855
Maize-41-RAM	41	Kenya	Ramogi	Plant	Grain	MixedYellowWhiteMaize	67.500	4.124	19.503	1061.290	0.008	25.102

Sample_ID	Household_ID	Country	Locality	Sample_Type	Tissue	Category	Ca	Cu	Fe	Mg	Se	Zn
Maize-42-RAM	42	Kenya	Ramogi	Plant	Grain	WhiteMaize	22.207	4.055	15.898	859.688	0.095	16.836
Maize-42-RAM	42	Kenya	Ramogi	Plant	Grain	WhiteMaize	22.532	0.183	16.734	847.862	0.092	16.648
Maize-42-RAM	42	Kenya	Ramogi	Plant	Grain	WhiteMaize	34.049	3.315	16.881	986.831	0.108	18.605
Maize-43-RAM	43	Kenya	Ramogi	Plant	Grain	WhiteMaize	37.750	5.732	13.867	794.804	0.007	20.147
Maize-43-RAM	43	Kenya	Ramogi	Plant	Grain	WhiteMaize	41.922	ND	14.814	822.610	0.011	20.160
Maize-43-RAM	43	Kenya	Ramogi	Plant	Grain	WhiteMaize	110.317	3.212	16.847	901.868	0.022	25.016
Maize-44-RAM	44	Kenya	Ramogi	Plant	Grain	YellowMaize	49.445	1.240	13.598	1025.157	ND	13.038
Maize-44-RAM	44	Kenya	Ramogi	Plant	Grain	YellowMaize	56.641	ND	13.434	1004.770	ND	12.487
Maize-44-RAM	44	Kenya	Ramogi	Plant	Grain	YellowMaize	69.421	2.635	16.427	1275.837	0.006	16.186
Maize-46-MAL	46	Kenya	Malindi	Plant	Grain	WhiteMaize	81.968	2.747	9.296	895.610	ND	27.881
Maize-46-MAL	46	Kenya	Malindi	Plant	Grain	WhiteMaize	90.977	ND	10.440	946.399	0.001	28.346
Maize-46-MAL	46	Kenya	Malindi	Plant	Grain	WhiteMaize	106.149	2.729	11.118	996.938	0.007	31.976
FR-Baob-10-KIB	10	Kenya	Kibwezi	Plant	Fruit	Baobab	2366.575	0.000	11.152	1356.255	0.098	1.370
FR-Baob-10-KIB	10	Kenya	Kibwezi	Plant	Fruit	Baobab	2618.639	0.094	10.956	1481.976	0.094	1.496
FR-Baob-10-KIB	10	Kenya	Kibwezi	Plant	Fruit	Baobab	2624.861	2.048	11.568	1590.243	0.128	2.024
FR-Baob-17-MBO	17	Kenya	Mbololo	Plant	Fruit	Baobab	2629.787	7.272	5.935	831.572	0.147	11.178
FR-Baob-17-MBO	17	Kenya	Mbololo	Plant	Fruit	Baobab	2818.050	9.379	6.164	936.048	0.177	12.924
FR-Baob-17-MBO	17	Kenya	Mbololo	Plant	Fruit	Baobab	2891.423	8.113	6.403	907.417	0.151	12.204
FR-Baob-20-MBO	20	Kenya	Mbololo	Plant	Fruit	Baobab	5908.124	6.044	6.756	4010.524	0.156	10.208
FR-Baob-20-MBO	20	Kenya	Mbololo	Plant	Fruit	Baobab	6861.348	8.595	8.242	4778.235	0.202	12.842
FR-Baob-20-MBO	20	Kenya	Mbololo	Plant	Fruit	Baobab	7045.119	7.091	7.715	4828.593	0.182	11.741
FR-Baob-21-MBO	21	Kenya	Mbololo	Plant	Fruit	Baobab	2459.075	7.234	14.353	777.905	0.217	12.100
FR-Baob-21-MBO	21	Kenya	Mbololo	Plant	Fruit	Baobab	2516.233	9.124	12.759	835.559	0.258	13.345
FR-Baob-21-MBO	21	Kenya	Mbololo	Plant	Fruit	Baobab	2542.895	7.663	15.120	793.356	0.226	12.407
L-AM-14-KIB	14	Kenya	Kibwezi	Plant	Leaf	Amaranth	26486.925	7.181	677.121	11882.148	0.243	45.826
L-AM-14-KIB	14	Kenya	Kibwezi	Plant	Leaf	Amaranth	26953.847	9.045	704.892	12965.798	0.272	50.343
L-AM-14-KIB	14	Kenya	Kibwezi	Plant	Leaf	Amaranth	28009.561	7.510	709.366	12652.727	0.251	48.158
L-AM-1-KIB	1	Kenya	Kibwezi	Plant	Leaf	Amaranth	24304.275	6.234	402.400	12524.740	0.176	32.525
L-AM-1-KIB	1	Kenya	Kibwezi	Plant	Leaf	Amaranth	25697.841	6.368	433.228	13218.353	0.177	34.174
L-AM-1-KIB	1	Kenya	Kibwezi	Plant	Leaf	Amaranth	26585.253	9.261	385.118	13884.086	0.215	37.192
L-AM-44-RAM	44	Kenya	Ramogi	Plant	Leaf	Amaranth	29299.154	7.269	1129.360	16181.735	0.018	33.852
L-AM-44-RAM	44	Kenya	Ramogi	Plant	Leaf	Amaranth	30297.389	7.367	1373.519	16206.552	0.023	34.242
L-AM-44-RAM	44	Kenya	Ramogi	Plant	Leaf	Amaranth	30407.426	4.790	138.016	11743.837	1.339	32.244
L-AM-52-MAL	52	Kenya	Malindi	Plant	Leaf	Amaranth	25926.755	2.507	113.411	9708.764	1.081	26.504
L-AM-52-MAL	52	Kenya	Malindi	Plant	Leaf	Amaranth	29486.557	3.094	124.968	11084.200	1.215	29.405

Sample_ID	Household_ID	Country	Locality	Sample_Type	Tissue	Category	Ca	Cu	Fe	Mg	Se	Zn
L-AM-52-MAL	52	Kenya	Malindi	Plant	Leaf	Amaranth	31019.665	17.359	134.588	11875.304	1.378	33.168
L-AM-55-MAL	55	Kenya	Malindi	Plant	Leaf	Amaranth	24685.083	1.029	183.043	10145.385	3.145	32.049
L-AM-55-MAL	55	Kenya	Malindi	Plant	Leaf	Amaranth	26549.082	16.297	210.003	11181.362	3.469	37.582
L-AM-55-MAL	55	Kenya	Malindi	Plant	Leaf	Amaranth	26621.838	1.348	197.243	10747.763	3.230	33.418
L-AM-8-KIB	8	Kenya	Kibwezi	Plant	Leaf	Amaranth	25343.308	5.270	249.416	14435.623	0.320	23.416
L-AM-8-KIB	8	Kenya	Kibwezi	Plant	Leaf	Amaranth	26388.947	5.674	298.771	15103.377	0.326	24.298
L-AM-8-KIB	8	Kenya	Kibwezi	Plant	Leaf	Amaranth	26796.557	7.506	264.502	15895.774	0.370	27.159
L-BO-1-KIB	1	Kenya	Kibwezi	Plant	Leaf	Brasssica	29045.793	2.210	97.100	7256.431	6.443	16.951
L-BO-1-KIB	1	Kenya	Kibwezi	Plant	Leaf	Brasssica	29686.536	20.179	105.707	7750.580	7.265	19.576
L-BO-1-KIB	1	Kenya	Kibwezi	Plant	Leaf	Brasssica	29856.034	2.388	102.632	7409.947	6.669	17.655
L-BO-24-MBO	24	Kenya	Mbololo	Plant	Leaf	Brasssica	19201.832	2.211	90.783	5485.070	0.363	22.833
L-BO-24-MBO	24	Kenya	Mbololo	Plant	Leaf	Brasssica	20935.410	2.222	96.614	6052.026	0.382	24.120
L-BO-24-MBO	24	Kenya	Mbololo	Plant	Leaf	Brasssica	23920.254	17.464	118.276	7096.018	0.457	29.283
L-BO-44-RAM	44	Kenya	Ramogi	Plant	Leaf	Brasssica	36186.174	0.871	1111.689	8241.619	0.020	14.453
L-BO-44-RAM	44	Kenya	Ramogi	Plant	Leaf	Brasssica	38349.982	28.808	1007.913	9423.164	0.024	18.102
L-BO-44-RAM	44	Kenya	Ramogi	Plant	Leaf	Brasssica	40597.156	1.423	1597.836	8924.171	0.025	15.942
L-BO-8-KIB	8	Kenya	Kibwezi	Plant	Leaf	Brasssica	32806.504	3.429	96.147	7822.819	0.737	15.814
L-BO-8-KIB	8	Kenya	Kibwezi	Plant	Leaf	Brasssica	34068.613	1.191	102.590	8018.374	0.751	15.414
L-BO-8-KIB	8	Kenya	Kibwezi	Plant	Leaf	Brasssica	36551.602	26.117	118.395	9042.885	0.893	19.246
