# Supplementary material for: Variation in the mineral element concentration of Moringa oleifera Lam. and M. stenopetala (Bak. f.) Cuf.: Role in human nutrition
Source: PLoS One. 2017 Apr 7;12(4):e0175503. doi: 10.1371/journal.pone.0175503 (PMC5384779; doi:10.1371/journal.pone.0175503)
Supplement: S37 Table — (PDF) [file pone.0175503.s037.pdf]

S37 Table. Raw data on soil elemental concentration (mg kg^{-1}) and pH, and sample details.

Sample_ID	Household_ID	Country	Locality	Category	Ca	Cu	Fe	Mg	Se	Zn	pH
Soil-1-KIB	1	Kenya	Kibwezi	Soil	7627.700	38.006	32624.812	2813.809	0.674	63.854	7.810
Soil-1-KIB	1	Kenya	Kibwezi	Soil	5957.055	33.418	28169.591	2363.301	0.654	52.176	7.810
Soil-1-KIB	1	Kenya	Kibwezi	Soil	5691.282	32.960	26834.743	2252.383	0.636	48.244	7.810
Soil-2-KIB	2	Kenya	Kibwezi	Soil	10775.004	42.335	50284.735	3866.083	0.488	75.336	7.540
Soil-2-KIB	2	Kenya	Kibwezi	Soil	9646.055	41.251	48737.605	3510.372	0.521	67.046	7.540
Soil-2-KIB	2	Kenya	Kibwezi	Soil	14928.289	37.210	43873.738	4997.417	0.326	58.971	7.540
Soil-3-KIB	3	Kenya	Kibwezi	Soil	17511.614	41.471	51215.098	5822.171	0.303	70.574	7.480
Soil-3-KIB	3	Kenya	Kibwezi	Soil	8832.546	37.382	44066.457	3176.089	0.485	62.042	7.480
Soil-3-KIB	3	Kenya	Kibwezi	Soil	14768.112	36.086	44041.421	4668.065	0.307	57.827	7.480
Soil-4-KIB	4	Kenya	Kibwezi	Soil	5599.508	11.411	29552.116	1227.775	0.332	94.906	8.460
Soil-4-KIB	4	Kenya	Kibwezi	Soil	4248.066	10.492	26176.025	968.030	0.366	82.311	8.460
Soil-4-KIB	4	Kenya	Kibwezi	Soil	4564.131	11.405	27624.965	1086.239	0.403	89.250	8.460
Soil-5-KIB	5	Kenya	Kibwezi	Soil	14277.690	13.640	32174.945	4148.170	0.261	48.351	8.290
Soil-5-KIB	5	Kenya	Kibwezi	Soil	12754.781	11.996	29040.433	3614.642	0.312	36.804	8.290
Soil-5-KIB	5	Kenya	Kibwezi	Soil	11294.073	11.876	26530.590	3447.042	0.287	36.457	8.290
Soil-6-KIB	6	Kenya	Kibwezi	Soil	38003.096	37.426	46403.859	12326.768	0.484	84.512	8.350
Soil-6-KIB	6	Kenya	Kibwezi	Soil	35480.247	35.726	44460.291	10620.176	0.509	74.739	8.350
Soil-6-KIB	6	Kenya	Kibwezi	Soil	37158.938	35.962	47771.681	11827.505	0.528	76.894	8.350
Soil-7-KIB	7	Kenya	Kibwezi	Soil	18297.825	32.196	42915.674	5996.949	0.346	60.115	8.590
Soil-7-KIB	7	Kenya	Kibwezi	Soil	14902.207	28.881	37187.043	4851.887	0.345	50.469	8.590
Soil-7-KIB	7	Kenya	Kibwezi	Soil	14246.408	27.768	35733.912	4683.336	0.329	48.207	8.590
Soil-8-KIB	8	Kenya	Kibwezi	Soil	20337.547	18.777	26873.982	5258.399	0.418	45.048	8.320
Soil-9-KIB	9	Kenya	Kibwezi	Soil	12824.545	18.609	29161.623	2773.535	0.196	35.085	7.370
Soil-10-KIB	10	Kenya	Kibwezi	Soil	14384.587	15.439	25094.235	2562.223	0.194	35.325	7.280
Soil-11-KIB	11	Kenya	Kibwezi	Soil	23534.476	14.748	29343.347	6930.595	0.240	50.046	7.970
Soil-12-KIB	12	Kenya	Kibwezi	Soil	5552.185	27.357	34714.370	2705.803	0.423	39.772	6.970
Soil-13-KIB	13	Kenya	Kibwezi	Soil	7737.936	13.034	18399.321	1003.312	0.244	38.992	8.130
Soil-14-KIB	14	Kenya	Kibwezi	Soil	11675.721	24.609	33538.459	2297.570	0.382	65.347	7.740

Sample_ID	Household_ID	Country	Locality	Category	Ca	Cu	Fe	Mg	Se	Zn	pH
Soil-15-MBO	15	Kenya	Mbololo	Soil	18612.468	12.025	22981.613	4844.905	0.305	43.773	7.750
Soil-16-MBO	16	Kenya	Mbololo	Soil	15499.327	21.015	37236.338	5787.440	0.385	53.624	7.310
Soil-17-MBO	17	Kenya	Mbololo	Soil	14556.933	9.210	20398.745	2913.523	0.127	60.408	7.110
Soil-18-MBO	18	Kenya	Mbololo	Soil	14325.584	11.348	26565.843	4209.570	0.134	40.866	7.320
Soil-19-MBO	19	Kenya	Mbololo	Soil	15662.238	11.053	21862.543	3598.940	0.218	38.497	6.630
Soil-20-MBO	20	Kenya	Mbololo	Soil	12646.395	6.373	16637.183	1593.195	0.175	33.585	7.420
Soil-21-MBO	21	Kenya	Mbololo	Soil	8158.021	3.766	13104.357	979.712	0.134	24.483	7.500
Soil-22-MBO	22	Kenya	Mbololo	Soil	10525.003	5.422	19871.656	1446.943	0.185	34.790	7.360
Soil-23-MBO	23	Kenya	Mbololo	Soil	13567.304	16.959	31351.093	4117.117	0.442	54.634	7.920
Soil-23-MBO	23	Kenya	Mbololo	Soil	11931.601	17.286	30005.257	3794.580	0.469	47.446	7.920
Soil-23-MBO	23	Kenya	Mbololo	Soil	11191.120	16.462	28196.998	3649.691	0.469	46.435	7.920
Soil-24-MBO	24	Kenya	Mbololo	Soil	14654.650	16.441	31439.132	5271.022	0.464	49.746	7.930
Soil-24-MBO	24	Kenya	Mbololo	Soil	10504.647	13.003	24367.988	3708.312	0.386	33.486	7.930
Soil-24-MBO	24	Kenya	Mbololo	Soil	14293.906	17.933	32792.750	5421.242	0.551	46.638	7.930
Soil-25-MBO	25	Kenya	Mbololo	Soil	19372.891	9.242	26661.940	5113.575	0.235	50.271	8.050
Soil-25-MBO	25	Kenya	Mbololo	Soil	15412.465	7.993	21250.927	3914.760	0.226	38.981	8.050
Soil-25-MBO	25	Kenya	Mbololo	Soil	15729.374	8.040	22487.144	4088.253	0.224	39.322	8.050
Soil-26-MBO	26	Kenya	Mbololo	Soil	12872.538	6.774	16007.051	2038.487	0.143	30.722	7.630
Soil-26-MBO	26	Kenya	Mbololo	Soil	10114.587	5.243	12075.932	1555.054	0.148	20.995	7.630
Soil-26-MBO	26	Kenya	Mbololo	Soil	10790.453	4.825	13286.474	1658.370	0.218	19.897	7.630
Soil-27-MBO	27	Kenya	Mbololo	Soil	13010.696	6.815	22403.963	2607.258	0.170	32.334	7.250
Soil-27-MBO	27	Kenya	Mbololo	Soil	10410.497	6.246	18140.718	2101.878	0.164	22.948	7.250
Soil-27-MBO	27	Kenya	Mbololo	Soil	10202.377	6.844	17913.266	1982.182	0.161	23.211	7.250
Soil-28-MBO	28	Kenya	Mbololo	Soil	13735.928	9.903	20639.401	2678.585	0.226	55.583	7.720
Soil-28-MBO	28	Kenya	Mbololo	Soil	11508.745	9.385	18230.807	2220.150	0.211	47.500	7.720
Soil-28-MBO	28	Kenya	Mbololo	Soil	11532.985	9.329	17754.618	2221.164	0.210	47.348	7.720
Soil-29-MBO	29	Kenya	Mbololo	Soil	13414.308	13.207	24659.976	3098.799	0.305	38.463	7.490
Soil-29-MBO	29	Kenya	Mbololo	Soil	10273.054	11.800	20401.930	2430.616	0.273	29.164	7.490
Soil-29-MBO	29	Kenya	Mbololo	Soil	11506.226	13.592	22899.231	2724.972	0.314	32.263	7.490
Soil-30-MBO	30	Kenya	Mbololo	Soil	17747.869	8.143	22738.170	4290.818	0.202	43.729	8.100

Sample_ID	Household_ID	Country	Locality	Category	Ca	Cu	Fe	Mg	Se	Zn	pH
Soil-30-MBO	30	Kenya	Mbololo	Soil	14817.732	7.986	19850.166	3774.205	0.189	36.326	8.100
Soil-30-MBO	30	Kenya	Mbololo	Soil	14329.127	7.758	19613.773	3544.277	0.177	34.935	8.100
Soil-31-Baringo	31	Kenya	Baringo	Soil	10852.645	12.024	60830.091	4201.053	0.661	132.570	7.600
Soil-31-Baringo	31	Kenya	Baringo	Soil	9029.732	10.873	53431.900	3577.120	0.661	113.711	7.600
Soil-31-Baringo	31	Kenya	Baringo	Soil	8901.847	11.073	52829.842	3562.573	0.671	112.965	7.600
Soil-32-Baringo	32	Kenya	Baringo	Soil	12711.410	9.959	54820.397	4525.090	0.684	115.410	7.990
Soil-32-Baringo	32	Kenya	Baringo	Soil	12860.680	9.153	56530.643	4340.336	0.867	103.998	7.990
Soil-32-Baringo	32	Kenya	Baringo	Soil	11192.131	9.321	50058.391	4334.217	0.742	102.294	7.990
Soil-33-Baringo	33	Kenya	Baringo	Soil	58651.875	32.244	75528.849	23780.005	0.260	83.502	8.080
Soil-34-Baringo	34	Kenya	Baringo	Soil	59681.313	34.610	79060.505	23665.378	0.218	85.245	7.960
Soil-35-Baringo	35	Kenya	Baringo	Soil	51983.153	31.062	67019.863	19500.581	0.843	92.914	7.770
Soil-36-Baringo	36	Kenya	Baringo	Soil	15603.976	21.462	64873.287	7242.577	0.460	141.534	8.020
Soil-37-Ramogi	37	Kenya	Ramogi	Soil	3445.009	90.307	111491.048	3134.211	0.739	102.812	7.650
Soil-38-Ramogi	38	Kenya	Ramogi	Soil	3897.753	17.324	40963.028	1943.555	0.485	55.431	7.850
Soil-39-Ramogi	39	Kenya	Ramogi	Soil	4977.855	22.495	46519.668	2089.150	0.642	76.242	8.030
Soil-40-Ramogi	40	Kenya	Ramogi	Soil	7312.717	21.471	42824.909	2879.440	0.529	151.230	7.810
Soil-40-Ramogi	40	Kenya	Ramogi	Soil	4502.836	19.973	34752.422	2314.478	0.484	147.838	7.810
Soil-40-Ramogi	40	Kenya	Ramogi	Soil	4247.108	19.111	33541.860	2164.511	0.473	114.656	7.810
Soil-41-Ramogi	41	Kenya	Ramogi	Soil	6456.069	41.567	65167.213	5477.947	0.573	124.830	7.660
Soil-41-Ramogi	41	Kenya	Ramogi	Soil	3333.115	36.588	52486.947	4140.888	0.454	105.735	7.660
Soil-41-Ramogi	41	Kenya	Ramogi	Soil	3869.092	39.090	55856.388	4472.001	0.511	106.247	7.660
Soil-42-Ramogi	42	Kenya	Ramogi	Soil	2388.716	20.194	56476.478	1250.921	0.538	94.738	7.800
Soil-42-Ramogi	42	Kenya	Ramogi	Soil	2000.735	18.822	53155.408	1111.337	0.550	82.632	7.800
Soil-42-Ramogi	42	Kenya	Ramogi	Soil	1943.062	18.891	52604.588	1082.565	0.546	83.395	7.800
Soil-43-Ramogi	43	Kenya	Ramogi	Soil	1286.314	21.678	56481.594	1168.753	0.597	63.587	7.330
Soil-43-Ramogi	43	Kenya	Ramogi	Soil	1210.617	19.229	52164.767	1036.200	0.620	52.176	7.330
Soil-43-Ramogi	43	Kenya	Ramogi	Soil	1115.433	19.368	50470.998	1013.016	0.579	54.129	7.330
Soil-44-Ramogi	44	Kenya	Ramogi	Soil	2952.350	54.101	145879.292	1167.444	0.350	76.283	8.380
Soil-44-Ramogi	44	Kenya	Ramogi	Soil	2815.074	55.131	150846.563	1144.142	0.413	73.229	8.380
Soil-44-Ramogi	44	Kenya	Ramogi	Soil	2714.108	55.826	152976.751	1138.313	0.383	73.769	8.380

Sample_ID	Household_ID	Country	Locality	Category	Ca	Cu	Fe	Mg	Se	Zn	pH
Soil-45-Malindi	45	Kenya	Malindi	Soil	15169.168	7.038	16893.194	742.147	0.136	49.635	8.640
Soil-46-Malindi	46	Kenya	Malindi	Soil	26982.538	6.752	14279.892	1076.151	0.118	55.522	8.620
Soil-46-Malindi	46	Kenya	Malindi	Soil	26327.219	6.348	14976.864	1098.549	0.172	54.465	8.620
Soil-46-Malindi	46	Kenya	Malindi	Soil	25648.523	5.897	14128.629	989.688	0.154	50.383	8.620
Soil-47-Malindi	47	Kenya	Malindi	Soil	5589.809	6.148	19567.822	747.916	0.188	51.323	8.310
Soil-47-Malindi	47	Kenya	Malindi	Soil	4719.349	5.709	18152.527	655.673	0.200	43.169	8.310
Soil-47-Malindi	47	Kenya	Malindi	Soil	4876.874	5.047	17669.002	665.345	0.207	41.762	8.310
Soil-48-Malindi	48	Kenya	Malindi	Soil	4431.006	6.924	19311.851	667.101	0.167	61.449	8.440
Soil-48-Malindi	48	Kenya	Malindi	Soil	3702.558	4.942	15743.372	532.018	0.165	47.296	8.440
Soil-48-Malindi	48	Kenya	Malindi	Soil	3871.386	5.148	16889.889	572.719	0.170	49.658	8.440
Soil-49-Malindi	49	Kenya	Malindi	Soil	17818.952	8.178	22908.156	1007.756	0.178	67.295	8.450
Soil-49-Malindi	49	Kenya	Malindi	Soil	15867.317	6.892	20116.139	802.289	0.203	52.056	8.450
Soil-49-Malindi	49	Kenya	Malindi	Soil	15755.597	7.044	20271.333	836.739	0.209	53.120	8.450
Soil-50-Malindi	50	Kenya	Malindi	Soil	6984.888	7.407	18418.477	736.133	0.194	45.182	8.650
Soil-50-Malindi	50	Kenya	Malindi	Soil	6004.045	5.816	15439.135	647.351	0.209	32.406	8.650
Soil-50-Malindi	50	Kenya	Malindi	Soil	6125.516	6.802	16839.703	653.024	0.213	36.096	8.650
Soil-51-Malindi	51	Kenya	Malindi	Soil	2180.746	3.565	6395.591	316.170	0.243	66.631	8.440
Soil-52-Malindi	52	Kenya	Malindi	Soil	1589.340	2.892	7245.284	453.297	0.177	15.068	8.510
Soil-53-Malindi	53	Kenya	Malindi	Soil	651.092	1.529	4085.513	214.004	0.096	12.632	7.960
Soil-54-Malindi	54	Kenya	Malindi	Soil	834.181	2.146	5306.445	242.975	0.160	13.006	8.110
Soil-55-Malindi	55	Kenya	Malindi	Soil	1509.710	3.007	6317.486	358.858	0.239	34.862	8.360
Soil-56-Ukunda	56	Kenya	Ukunda	Soil	25606.797	6.112	7108.539	869.674	0.113	102.307	8.780
Soil-57-Ukunda	57	Kenya	Ukunda	Soil	14636.954	8.420	8035.247	843.560	0.114	115.146	7.810
Soil-58-Ukunda	58	Kenya	Ukunda	Soil	8408.638	6.513	7829.214	793.699	0.119	76.887	7.880
Soil-59-Ukunda	59	Kenya	Ukunda	Soil	17733.142	9.147	8141.791	887.839	0.169	113.213	7.830
Soil-60-Ukunda	60	Kenya	Ukunda	Soil	17907.032	8.750	8102.908	864.168	0.154	111.231	7.860
Soil-60-Ukunda	60	Kenya	Ukunda	Soil	17105.460	9.245	7837.718	803.560	0.173	107.704	7.860
Soil-60-Ukunda	60	Kenya	Ukunda	Soil	17729.114	9.661	8274.605	849.452	0.190	110.042	7.860
Soil-61-Ukunda	61	Kenya	Ukunda	Soil	4296.430	8.045	10800.235	488.226	0.255	92.696	7.800
Soil-61-Ukunda	61	Kenya	Ukunda	Soil	3379.819	6.781	8611.453	373.732	0.206	70.982	7.800

Sample_ID	Household_ID	Country	Locality	Category	Ca	Cu	Fe	Mg	Se	Zn	pH
Soil-61-Ukunda	61	Kenya	Ukunda	Soil	3035.863	6.395	7943.203	344.894	0.198	60.387	7.800
Soil-62-Ukunda	62	Kenya	Ukunda	Soil	2551.247	6.980	5722.441	385.279	0.167	58.224	7.600
Soil-62-Ukunda	62	Kenya	Ukunda	Soil	2319.920	6.257	5465.523	368.432	0.181	54.535	7.600
Soil-62-Ukunda	62	Kenya	Ukunda	Soil	2352.475	5.892	5325.654	358.318	0.184	50.746	7.600
ETS0001	ETH001	Ethiopia	Derashe	Soil	28077.077	42.739	72418.753	19375.443	0.191	94.741	8.520
ETS0001	ETH001	Ethiopia	Derashe	Soil	24242.471	37.680	64649.962	15801.780	0.223	76.465	8.520
ETS0001	ETH001	Ethiopia	Derashe	Soil	25538.599	41.005	69624.818	16054.472	0.231	83.258	8.520
ETS0002	ETH002	Ethiopia	Derashe	Soil	32588.013	41.365	94008.165	17497.654	0.388	122.281	8.360
ETS0002	ETH002	Ethiopia	Derashe	Soil	31348.368	40.101	92989.775	15538.428	0.465	112.186	8.360
ETS0002	ETH002	Ethiopia	Derashe	Soil	29895.219	39.301	90612.545	14474.274	0.445	107.830	8.360
ETS0003	ETH003	Ethiopia	Derashe	Soil	34560.184	35.441	106027.917	16206.405	0.271	133.718	8.500
ETS0003	ETH003	Ethiopia	Derashe	Soil	29831.800	32.367	96170.227	12028.575	0.326	113.476	8.500
ETS0003	ETH003	Ethiopia	Derashe	Soil	32277.974	33.097	101502.372	14550.317	0.353	117.562	8.500
ETS0004	ETH004	Ethiopia	Derashe	Soil	40577.502	31.601	78992.323	17776.011	0.146	93.878	8.640
ETS0004	ETH004	Ethiopia	Derashe	Soil	37597.474	29.982	75258.401	14319.739	0.187	80.151	8.640
ETS0004	ETH004	Ethiopia	Derashe	Soil	36884.573	30.399	74718.827	13671.390	0.184	78.838	8.640
ETS0005	ETH005	Ethiopia	Derashe	Soil	36223.491	30.475	77152.152	17336.855	0.132	86.983	8.670
ETS0005	ETH005	Ethiopia	Derashe	Soil	32459.939	29.498	72492.808	12560.341	0.167	74.948	8.670
ETS0005	ETH005	Ethiopia	Derashe	Soil	32042.167	28.928	71172.277	12880.410	0.158	73.329	8.670
ETS0006	ETH006	Ethiopia	Derashe	Soil	33189.425	30.957	76214.811	19021.016	0.329	96.525	8.460
ETS0007	ETH007	Ethiopia	Derashe	Soil	29942.888	31.191	83473.702	14387.441	0.352	106.641	8.560
ETS0008	ETH008	Ethiopia	Derashe	Soil	39399.055	31.220	82228.131	17364.918	0.206	100.552	8.620
ETS0009	ETH009	Ethiopia	Derashe	Soil	29162.279	33.498	67730.158	17735.337	0.229	100.715	8.280
ETS0010	ETH010	Ethiopia	Derashe	Soil	3748.715	30.729	34226.635	1399.504	0.218	41.849	7.640
ETS0011	ETH011	Ethiopia	Derashe	Soil	15045.164	22.956	27817.561	5537.352	0.213	51.653	8.380
ETS0012	ETH012	Ethiopia	Derashe	Soil	28969.429	45.387	45971.964	11813.571	0.225	74.186	8.190
ETS0013	ETH013	Ethiopia	Konso	Soil	25780.033	27.928	51189.400	5425.763	0.133	62.488	8.420
ETS0014	ETH014	Ethiopia	Konso	Soil	21492.383	36.170	75646.737	6704.319	0.231	83.676	7.980
ETS0015	ETH015	Ethiopia	Konso	Soil	26680.707	48.195	74978.552	7270.137	0.216	93.603	8.280
ETS0016	ETH016	Ethiopia	Konso	Soil	32158.264	42.468	56603.165	6441.715	0.154	68.605	7.820

Sample_ID	Household_ID	Country	Locality	Category	Ca	Cu	Fe	Mg	Se	Zn	pH
ETS0016	ETH016	Ethiopia	Konso	Soil	27165.397	36.514	46146.008	4466.989	0.138	128.398	7.820
ETS0016	ETH016	Ethiopia	Konso	Soil	26286.025	35.842	46164.173	4636.405	0.135	50.303	7.820
ETS0017	ETH017	Ethiopia	Konso	Soil	16124.159	2.450	30119.164	3169.047	0.222	60.782	7.260
ETS0017	ETH017	Ethiopia	Konso	Soil	12417.464	1.617	26518.788	2447.198	0.183	44.251	7.260
ETS0017	ETH017	Ethiopia	Konso	Soil	12794.706	1.573	24911.839	2407.815	0.172	43.577	7.260
ETS0018	ETH018	Ethiopia	Konso	Soil	7157.586	4.606	18519.328	2577.112	0.162	46.469	7.420
ETS0018	ETH018	Ethiopia	Konso	Soil	5606.968	4.264	15484.004	2122.780	0.167	36.499	7.420
ETS0018	ETH018	Ethiopia	Konso	Soil	5667.647	4.217	15402.303	2068.224	0.167	36.327	7.420
ETS0019	ETH019	Ethiopia	Konso	Soil	6902.044	4.633	22329.059	2235.491	0.184	47.489	6.180
ETS0019	ETH019	Ethiopia	Konso	Soil	5194.909	4.260	16511.619	1677.712	0.179	36.941	6.180
ETS0019	ETH019	Ethiopia	Konso	Soil	5503.666	4.039	17670.009	1801.648	0.181	36.331	6.180
ETS0020	ETH020	Ethiopia	Konso	Soil	6222.781	2.537	13215.345	1567.554	0.196	32.320	7.560
ETS0020	ETH020	Ethiopia	Konso	Soil	5704.667	1.981	11214.645	1362.222	0.184	21.768	7.560
ETS0020	ETH020	Ethiopia	Konso	Soil	4079.539	1.980	9025.022	1180.504	0.166	21.036	7.560
ETS0021	ETH021	Ethiopia	Konso	Soil	20676.597	33.812	75579.770	12680.809	0.442	134.215	7.530
ETS0022	ETH022	Ethiopia	Konso	Soil	24746.846	38.855	96930.589	13673.023	0.281	127.773	7.620
ETS0023	ETH023	Ethiopia	Konso	Soil	23256.685	39.681	86387.135	13842.646	0.381	159.458	7.200
ETS0024	ETH024	Ethiopia	Konso	Soil	22135.113	39.045	87964.940	12448.326	0.316	130.452	7.870
ETS0025	Eth-Haw-1	Ethiopia	Hawasa	Soil	6600.666	7.538	31863.055	3376.803	0.799	243.695	7.090
ETS0026	Eth-Haw-2	Ethiopia	Hawasa	Soil	22288.739	7.133	30231.446	3127.653	0.463	262.627	7.920
ETS0027	Eth-Haw-3	Ethiopia	Hawasa	Soil	14126.779	8.624	43262.131	6430.471	0.478	352.282	7.510
ETS0028	Eth-Haw-4	Ethiopia	Hawasa	Soil	4682.805	4.373	23331.785	1977.243	0.363	121.331	6.130
ETS0029	Eth-Haw-5	Ethiopia	Hawasa	Soil	11043.469	11.411	36593.130	4262.276	0.655	256.933	6.120
ETS0029	Eth-Haw-5	Ethiopia	Hawasa	Soil	9197.071	9.686	30845.012	3555.585	0.762	215.220	6.120
ETS0029	Eth-Haw-5	Ethiopia	Hawasa	Soil	9337.499	9.635	31268.337	3576.723	0.768	213.787	6.120
ETS0030	Eth-Haw-6	Ethiopia	Hawasa	Soil	21433.560	10.311	26894.236	2223.025	0.331	142.341	8.380
ETS0030	Eth-Haw-6	Ethiopia	Hawasa	Soil	20262.943	10.033	24113.296	1633.850	0.429	133.397	8.380
ETS0030	Eth-Haw-6	Ethiopia	Hawasa	Soil	17031.137	8.024	20214.434	1485.164	0.372	111.275	8.380
ETS0031	Eth-Haw-7	Ethiopia	Hawasa	Soil	41476.819	54.940	35329.822	6696.627	0.691	671.991	7.980
ETS0031	Eth-Haw-7	Ethiopia	Hawasa	Soil	38745.519	52.159	33907.850	6661.991	0.854	617.190	7.980

Sample_ID	Household_ID	Country	Locality	Category	Ca	Cu	Fe	Mg	Se	Zn	pH
ETS0031	Eth-Haw-7	Ethiopia	Hawasa	Soil	36927.895	50.019	32408.881	5515.269	0.809	592.011	7.980
ETS0032	Eth-Haw-8	Ethiopia	Hawasa	Soil	8360.773	8.262	39131.076	4853.149	0.664	228.923	7.660
ETS0032	Eth-Haw-8	Ethiopia	Hawasa	Soil	7511.685	6.496	35335.160	4247.408	0.819	192.815	7.660
ETS0032	Eth-Haw-8	Ethiopia	Hawasa	Soil	8254.888	7.579	39233.830	4728.343	0.845	222.891	7.660
ETS0033	Eth-Haw-9	Ethiopia	Hawasa	Soil	18568.843	11.505	63299.903	12709.599	0.531	168.868	8.040
ETS0033	Eth-Haw-9	Ethiopia	Hawasa	Soil	18014.407	8.581	62461.841	12371.054	0.695	156.220	8.040
ETS0033	Eth-Haw-9	Ethiopia	Hawasa	Soil	16067.416	8.026	58535.300	10245.405	0.640	146.543	8.040
