# Supplementary material for: Variation in the mineral element concentration of Moringa oleifera Lam. and M. stenopetala (Bak. f.) Cuf.: Role in human nutrition
Source: PLoS One. 2017 Apr 7;12(4):e0175503. doi: 10.1371/journal.pone.0175503 (PMC5384779; doi:10.1371/journal.pone.0175503)
Supplement: S38 Table — (PDF) [file pone.0175503.s038.pdf]

S38 Table. Raw data on soil iodine concentration (mg kg^{-1}) and sample details.

Sample_ID	Household_ID	Country	Locality	Category	Iodine
Soil-1-KIB	1	Kenya	Kibwezi	Soil	3.42869
Soil-1-KIB	1	Kenya	Kibwezi	Soil	3.96264
Soil-1-KIB	1	Kenya	Kibwezi	Soil	2.96113
Soil-2-KIB	2	Kenya	Kibwezi	Soil	1.79708
Soil-2-KIB	2	Kenya	Kibwezi	Soil	1.62209
Soil-2-KIB	2	Kenya	Kibwezi	Soil	1.67632
Soil-3-KIB	3	Kenya	Kibwezi	Soil	1.54943
Soil-3-KIB	3	Kenya	Kibwezi	Soil	1.32277
Soil-3-KIB	3	Kenya	Kibwezi	Soil	1.79132
Soil-4-KIB	4	Kenya	Kibwezi	Soil	0.56271
Soil-4-KIB	4	Kenya	Kibwezi	Soil	0.55665
Soil-4-KIB	4	Kenya	Kibwezi	Soil	0.55195
Soil-5-KIB	5	Kenya	Kibwezi	Soil	2.08241
Soil-5-KIB	5	Kenya	Kibwezi	Soil	2.06814
Soil-6-KIB	6	Kenya	Kibwezi	Soil	3.08071
Soil-6-KIB	6	Kenya	Kibwezi	Soil	2.87827
Soil-7-KIB	7	Kenya	Kibwezi	Soil	2.68990
Soil-7-KIB	7	Kenya	Kibwezi	Soil	2.42229
Soil-8-KIB	8	Kenya	Kibwezi	Soil	1.00154
Soil-8-KIB	8	Kenya	Kibwezi	Soil	0.93713
Soil-9-KIB	9	Kenya	Kibwezi	Soil	1.15337
Soil-9-KIB	9	Kenya	Kibwezi	Soil	1.06876
Soil-10-KIB	10	Kenya	Kibwezi	Soil	0.62183
Soil-10-KIB	10	Kenya	Kibwezi	Soil	0.77786
Soil-11-KIB	11	Kenya	Kibwezi	Soil	0.65942
Soil-11-KIB	11	Kenya	Kibwezi	Soil	0.73179
Soil-12-KIB	12	Kenya	Kibwezi	Soil	3.74158
Soil-12-KIB	12	Kenya	Kibwezi	Soil	3.60225

**Sample_ID**	**Household_ID**	**Country**	**Locality**	**Category**	**Iodine**
Soil-13-KIB	13	Kenya	Kibwezi	Soil	0.75745
Soil-13-KIB	13	Kenya	Kibwezi	Soil	0.80057
Soil-14-KIB	14	Kenya	Kibwezi	Soil	1.18900
Soil-14-KIB	14	Kenya	Kibwezi	Soil	1.32767
Soil-15-MBO	15	Kenya	Mbololo	Soil	0.74078
Soil-15-MBO	15	Kenya	Mbololo	Soil	0.68210
Soil-16-MBO	16	Kenya	Mbololo	Soil	2.28845
Soil-16-MBO	16	Kenya	Mbololo	Soil	2.15915
Soil-17-MBO	17	Kenya	Mbololo	Soil	1.12702
Soil-17-MBO	17	Kenya	Mbololo	Soil	1.07026
Soil-18-MBO	18	Kenya	Mbololo	Soil	2.41610
Soil-18-MBO	18	Kenya	Mbololo	Soil	1.99932
Soil-19-MBO	19	Kenya	Mbololo	Soil	1.79054
Soil-19-MBO	19	Kenya	Mbololo	Soil	1.31949
Soil-20-MBO	20	Kenya	Mbololo	Soil	0.91212
Soil-20-MBO	20	Kenya	Mbololo	Soil	0.86872
Soil-21-MBO	21	Kenya	Mbololo	Soil	0.70451
Soil-21-MBO	21	Kenya	Mbololo	Soil	0.82833
Soil-22-MBO	22	Kenya	Mbololo	Soil	1.14107
Soil-22-MBO	22	Kenya	Mbololo	Soil	1.24880
Soil-23-MBO	23	Kenya	Mbololo	Soil	2.26809
Soil-23-MBO	23	Kenya	Mbololo	Soil	1.97624
Soil-24-MBO	24	Kenya	Mbololo	Soil	2.47866
Soil-24-MBO	24	Kenya	Mbololo	Soil	3.06745
Soil-25-MBO	25	Kenya	Mbololo	Soil	1.23858
Soil-25-MBO	25	Kenya	Mbololo	Soil	1.56329
Soil-26-MBO	26	Kenya	Mbololo	Soil	0.82366
Soil-26-MBO	26	Kenya	Mbololo	Soil	0.58436
Soil-27-MBO	27	Kenya	Mbololo	Soil	1.79319
Soil-27-MBO	27	Kenya	Mbololo	Soil	1.27718

Sample_ID	Household_ID	Country	Locality	Category	Iodine
Soil-28-MBO	28	Kenya	Mbololo	Soil	1.20724
Soil-28-MBO	28	Kenya	Mbololo	Soil	1.14884
Soil-29-MBO	29	Kenya	Mbololo	Soil	1.83487
Soil-29-MBO	29	Kenya	Mbololo	Soil	2.03751
Soil-30-MBO	30	Kenya	Mbololo	Soil	0.66787
Soil-30-MBO	30	Kenya	Mbololo	Soil	0.62757
Soil-31-Baringo	31	Kenya	Baringo	Soil	1.69867
Soil-31-Baringo	31	Kenya	Baringo	Soil	1.21961
Soil-32-Baringo	32	Kenya	Baringo	Soil	1.13947
Soil-32-Baringo	32	Kenya	Baringo	Soil	0.98498
Soil-33-Baringo	33	Kenya	Baringo	Soil	0.25967
Soil-33-Baringo	33	Kenya	Baringo	Soil	0.33000
Soil-34-Baringo	34	Kenya	Baringo	Soil	0.09840
Soil-34-Baringo	34	Kenya	Baringo	Soil	0.08435
Soil-35-Baringo	35	Kenya	Baringo	Soil	1.65617
Soil-35-Baringo	35	Kenya	Baringo	Soil	1.76240
Soil-36-Baringo	36	Kenya	Baringo	Soil	0.96586
Soil-36-Baringo	36	Kenya	Baringo	Soil	0.98891
Soil-37-Ramogi	37	Kenya	Ramogi	Soil	4.39700
Soil-37-Ramogi	37	Kenya	Ramogi	Soil	4.73974
Soil-38-Ramogi	38	Kenya	Ramogi	Soil	1.72521
Soil-38-Ramogi	38	Kenya	Ramogi	Soil	1.63191
Soil-39-Ramogi	39	Kenya	Ramogi	Soil	2.39414
Soil-39-Ramogi	39	Kenya	Ramogi	Soil	2.27285
Soil-40-Ramogi	40	Kenya	Ramogi	Soil	3.54347
Soil-40-Ramogi	40	Kenya	Ramogi	Soil	3.04094
Soil-41-Ramogi	41	Kenya	Ramogi	Soil	7.00586
Soil-41-Ramogi	41	Kenya	Ramogi	Soil	7.14614
Soil-42-Ramogi	42	Kenya	Ramogi	Soil	2.41435
Soil-42-Ramogi	42	Kenya	Ramogi	Soil	2.07904

Sample_ID	Household_ID	Country	Locality	Category	Iodine
Soil-43-Ramogi	43	Kenya	Ramogi	Soil	3.01528
Soil-43-Ramogi	43	Kenya	Ramogi	Soil	5.19442
Soil-44-Ramogi	44	Kenya	Ramogi	Soil	0.60336
Soil-44-Ramogi	44	Kenya	Ramogi	Soil	0.56424
Soil-45-Malindi	45	Kenya	Malindi	Soil	1.77279
Soil-45-Malindi	45	Kenya	Malindi	Soil	1.49480
Soil-46-Malindi	46	Kenya	Malindi	Soil	0.74933
Soil-46-Malindi	46	Kenya	Malindi	Soil	0.58709
Soil-47-Malindi	47	Kenya	Malindi	Soil	1.25487
Soil-47-Malindi	47	Kenya	Malindi	Soil	1.10901
Soil-48-Malindi	48	Kenya	Malindi	Soil	0.74944
Soil-48-Malindi	48	Kenya	Malindi	Soil	0.86493
Soil-49-Malindi	49	Kenya	Malindi	Soil	0.95163
Soil-49-Malindi	49	Kenya	Malindi	Soil	0.90163
Soil-50-Malindi	50	Kenya	Malindi	Soil	1.68716
Soil-50-Malindi	50	Kenya	Malindi	Soil	1.61272
Soil-51-Malindi	51	Kenya	Malindi	Soil	0.79013
Soil-51-Malindi	51	Kenya	Malindi	Soil	0.72041
Soil-52-Malindi	52	Kenya	Malindi	Soil	0.74949
Soil-52-Malindi	52	Kenya	Malindi	Soil	0.76983
Soil-53-Malindi	53	Kenya	Malindi	Soil	1.11657
Soil-53-Malindi	53	Kenya	Malindi	Soil	0.35693
Soil-54-Malindi	54	Kenya	Malindi	Soil	0.58578
Soil-54-Malindi	54	Kenya	Malindi	Soil	0.64441
Soil-55-Malindi	55	Kenya	Malindi	Soil	0.73728
Soil-55-Malindi	55	Kenya	Malindi	Soil	0.79966
Soil-56-Ukunda	56	Kenya	Ukunda	Soil	1.45027
Soil-56-Ukunda	56	Kenya	Ukunda	Soil	1.45989
Soil-57-Ukunda	57	Kenya	Ukunda	Soil	0.71234
Soil-57-Ukunda	57	Kenya	Ukunda	Soil	0.75225

**Sample_ID**	**Household_ID**	**Country**	**Locality**	**Category**	**Iodine**
Soil-58-Ukunda	58	Kenya	Ukunda	Soil	0.92536
Soil-58-Ukunda	58	Kenya	Ukunda	Soil	0.92058
Soil-59-Ukunda	59	Kenya	Ukunda	Soil	1.34626
Soil-59-Ukunda	59	Kenya	Ukunda	Soil	1.41516
Soil-60-Ukunda	60	Kenya	Ukunda	Soil	1.34665
Soil-60-Ukunda	60	Kenya	Ukunda	Soil	1.20070
Soil-61-Ukunda	61	Kenya	Ukunda	Soil	0.87054
Soil-61-Ukunda	61	Kenya	Ukunda	Soil	0.86185
Soil-62-Ukunda	62	Kenya	Ukunda	Soil	0.35555
Soil-62-Ukunda	62	Kenya	Ukunda	Soil	0.39084
ETS0001	ETH001	Ethiopia	Derashe	Soil	0.93367
ETS0001	ETH001	Ethiopia	Derashe	Soil	0.91820
ETS0002	ETH002	Ethiopia	Derashe	Soil	0.79008
ETS0002	ETH002	Ethiopia	Derashe	Soil	0.79358
ETS0003	ETH003	Ethiopia	Derashe	Soil	0.30929
ETS0003	ETH003	Ethiopia	Derashe	Soil	0.32862
ETS0004	ETH004	Ethiopia	Derashe	Soil	0.23356
ETS0004	ETH004	Ethiopia	Derashe	Soil	0.23451
ETS0005	ETH005	Ethiopia	Derashe	Soil	0.19685
ETS0005	ETH005	Ethiopia	Derashe	Soil	0.19553
ETS0006	ETH006	Ethiopia	Derashe	Soil	1.28796
ETS0006	ETH006	Ethiopia	Derashe	Soil	1.37637
ETS0007	ETH007	Ethiopia	Derashe	Soil	2.32858
ETS0007	ETH007	Ethiopia	Derashe	Soil	2.33631
ETS0008	ETH008	Ethiopia	Derashe	Soil	0.68671
ETS0008	ETH008	Ethiopia	Derashe	Soil	0.72308
ETS0009	ETH009	Ethiopia	Derashe	Soil	1.24147
ETS0009	ETH009	Ethiopia	Derashe	Soil	1.34596
ETS0010	ETH010	Ethiopia	Derashe	Soil	1.08938
ETS0010	ETH010	Ethiopia	Derashe	Soil	1.05000

**Sample_ID**	**Household_ID**	**Country**	**Locality**	**Category**	**Iodine**
ETS0011	ETH011	Ethiopia	Derashe	Soil	0.41882
ETS0011	ETH011	Ethiopia	Derashe	Soil	0.42674
ETS0012	ETH012	Ethiopia	Derashe	Soil	0.58302
ETS0012	ETH012	Ethiopia	Derashe	Soil	0.58379
ETS0013	ETH013	Ethiopia	Konso	Soil	0.33683
ETS0013	ETH013	Ethiopia	Konso	Soil	0.36547
ETS0014	ETH014	Ethiopia	Konso	Soil	0.99244
ETS0014	ETH014	Ethiopia	Konso	Soil	0.91996
ETS0015	ETH015	Ethiopia	Konso	Soil	0.74530
ETS0015	ETH015	Ethiopia	Konso	Soil	0.73036
ETS0016	ETH016	Ethiopia	Konso	Soil	0.42527
ETS0016	ETH016	Ethiopia	Konso	Soil	0.41910
ETS0017	ETH017	Ethiopia	Konso	Soil	0.59623
ETS0017	ETH017	Ethiopia	Konso	Soil	0.63250
ETS0018	ETH018	Ethiopia	Konso	Soil	0.45492
ETS0018	ETH018	Ethiopia	Konso	Soil	0.45358
ETS0019	ETH019	Ethiopia	Konso	Soil	0.82968
ETS0019	ETH019	Ethiopia	Konso	Soil	0.93344
ETS0020	ETH020	Ethiopia	Konso	Soil	0.60158
ETS0020	ETH020	Ethiopia	Konso	Soil	0.57387
ETS0021	ETH021	Ethiopia	Konso	Soil	1.68150
ETS0021	ETH021	Ethiopia	Konso	Soil	1.47160
ETS0022	ETH022	Ethiopia	Konso	Soil	0.42335
ETS0022	ETH022	Ethiopia	Konso	Soil	0.40754
ETS0023	ETH023	Ethiopia	Konso	Soil	1.25912
ETS0023	ETH023	Ethiopia	Konso	Soil	1.30597
ETS0024	ETH024	Ethiopia	Konso	Soil	1.06265
ETS0024	ETH024	Ethiopia	Konso	Soil	0.88546
ETS0025	Eth-Haw-1	Ethiopia	Hawasa	Soil	0.91676
ETS0025	Eth-Haw-1	Ethiopia	Hawasa	Soil	0.96598

Sample_ID	Household_ID	Country	Locality	Category	Iodine
ETS0026	Eth-Haw-2	Ethiopia	Hawasa	Soil	0.72221
ETS0026	Eth-Haw-2	Ethiopia	Hawasa	Soil	0.82444
ETS0027	Eth-Haw-3	Ethiopia	Hawasa	Soil	0.71981
ETS0027	Eth-Haw-3	Ethiopia	Hawasa	Soil	0.66531
ETS0028	Eth-Haw-4	Ethiopia	Hawasa	Soil	0.55396
ETS0028	Eth-Haw-4	Ethiopia	Hawasa	Soil	0.73837
ETS0029	Eth-Haw-5	Ethiopia	Hawasa	Soil	1.50937
ETS0029	Eth-Haw-5	Ethiopia	Hawasa	Soil	1.34908
ETS0030	Eth-Haw-6	Ethiopia	Hawasa	Soil	0.68152
ETS0030	Eth-Haw-6	Ethiopia	Hawasa	Soil	0.65739
ETS0031	Eth-Haw-7	Ethiopia	Hawasa	Soil	0.93632
ETS0031	Eth-Haw-7	Ethiopia	Hawasa	Soil	1.11799
ETS0032	Eth-Haw-8	Ethiopia	Hawasa	Soil	1.96519
ETS0032	Eth-Haw-8	Ethiopia	Hawasa	Soil	1.94215
ETS0033	Eth-Haw-9	Ethiopia	Hawasa	Soil	1.81314
ETS0033	Eth-Haw-9	Ethiopia	Hawasa	Soil	2.28444
2711A	999	Reference	Reference	Soil	1.04520
2711A	999	Reference	Reference	Soil	1.05798
2711A	999	Reference	Reference	Soil	1.06357
