# Supplementary material for: Variation in the mineral element concentration of Moringa oleifera Lam. and M. stenopetala (Bak. f.) Cuf.: Role in human nutrition
Source: PLoS One. 2017 Apr 7;12(4):e0175503. doi: 10.1371/journal.pone.0175503 (PMC5384779; doi:10.1371/journal.pone.0175503)
Supplement: S39 Table — (PDF) [file pone.0175503.s039.pdf]

S39 Table. Raw data on phosphate-extractable soil selenium (Se-P) concentration (mg kg^{-1}) and sample details.

Sample_ID	Household_ID	Country	Locality	Category	Se-P
Soil-1-KIB	1	Kenya	Kibwezi	Soil	0.03561
Soil-1-KIB	1	Kenya	Kibwezi	Soil	0.03888
Soil-1-KIB	1	Kenya	Kibwezi	Soil	0.04173
Soil-2-KIB	2	Kenya	Kibwezi	Soil	0.03270
Soil-2-KIB	2	Kenya	Kibwezi	Soil	0.03375
Soil-2-KIB	2	Kenya	Kibwezi	Soil	0.03294
Soil-3-KIB	3	Kenya	Kibwezi	Soil	0.02248
Soil-3-KIB	3	Kenya	Kibwezi	Soil	0.02293
Soil-3-KIB	3	Kenya	Kibwezi	Soil	0.02381
Soil-4-KIB	4	Kenya	Kibwezi	Soil	0.02950
Soil-4-KIB	4	Kenya	Kibwezi	Soil	0.03205
Soil-4-KIB	4	Kenya	Kibwezi	Soil	0.03327
Soil-5-KIB	5	Kenya	Kibwezi	Soil	0.01961
Soil-5-KIB	5	Kenya	Kibwezi	Soil	0.02088
Soil-6-KIB	6	Kenya	Kibwezi	Soil	0.02751
Soil-6-KIB	6	Kenya	Kibwezi	Soil	0.02863
Soil-7-KIB	7	Kenya	Kibwezi	Soil	0.02718
Soil-7-KIB	7	Kenya	Kibwezi	Soil	0.02905
Soil-8-KIB	8	Kenya	Kibwezi	Soil	0.02511
Soil-8-KIB	8	Kenya	Kibwezi	Soil	0.02482
Soil-9-KIB	9	Kenya	Kibwezi	Soil	0.01564
Soil-9-KIB	9	Kenya	Kibwezi	Soil	0.01544
Soil-10-KIB	10	Kenya	Kibwezi	Soil	0.01785
Soil-10-KIB	10	Kenya	Kibwezi	Soil	0.01880
Soil-11-KIB	11	Kenya	Kibwezi	Soil	0.02168
Soil-11-KIB	11	Kenya	Kibwezi	Soil	0.02350

Sample_ID	Household_ID	Country	Locality	Category	Se-P
Soil-12-KIB	12	Kenya	Kibwezi	Soil	0.01914
Soil-12-KIB	12	Kenya	Kibwezi	Soil	0.01967
Soil-13-KIB	13	Kenya	Kibwezi	Soil	0.01756
Soil-13-KIB	13	Kenya	Kibwezi	Soil	0.01848
Soil-14-KIB	14	Kenya	Kibwezi	Soil	0.01561
Soil-14-KIB	14	Kenya	Kibwezi	Soil	0.01731
Soil-15-MBO	15	Kenya	Mbololo	Soil	0.02031
Soil-15-MBO	15	Kenya	Mbololo	Soil	0.02339
Soil-16-MBO	16	Kenya	Mbololo	Soil	0.02860
Soil-16-MBO	16	Kenya	Mbololo	Soil	0.02847
Soil-17-MBO	17	Kenya	Mbololo	Soil	0.01673
Soil-17-MBO	17	Kenya	Mbololo	Soil	0.01665
Soil-18-MBO	18	Kenya	Mbololo	Soil	0.01470
Soil-18-MBO	18	Kenya	Mbololo	Soil	0.01440
Soil-19-MBO	19	Kenya	Mbololo	Soil	0.02079
Soil-19-MBO	19	Kenya	Mbololo	Soil	0.02052
Soil-20-MBO	20	Kenya	Mbololo	Soil	0.01801
Soil-20-MBO	20	Kenya	Mbololo	Soil	0.01880
Soil-21-MBO	21	Kenya	Mbololo	Soil	0.01468
Soil-21-MBO	21	Kenya	Mbololo	Soil	0.01573
Soil-22-MBO	22	Kenya	Mbololo	Soil	0.01362
Soil-22-MBO	22	Kenya	Mbololo	Soil	0.01445
Soil-23-MBO	23	Kenya	Mbololo	Soil	0.02807
Soil-23-MBO	23	Kenya	Mbololo	Soil	0.02731
Soil-24-MBO	24	Kenya	Mbololo	Soil	0.02995
Soil-24-MBO	24	Kenya	Mbololo	Soil	0.02986
Soil-25-MBO	25	Kenya	Mbololo	Soil	0.01654
Soil-25-MBO	25	Kenya	Mbololo	Soil	0.01702
Soil-26-MBO	26	Kenya	Mbololo	Soil	0.01523
Soil-26-MBO	26	Kenya	Mbololo	Soil	0.01689

Sample_ID	Household_ID	Country	Locality	Category	Se-P
Soil-27-MBO	27	Kenya	Mbololo	Soil	0.01825
Soil-27-MBO	27	Kenya	Mbololo	Soil	0.02015
Soil-28-MBO	28	Kenya	Mbololo	Soil	0.01736
Soil-28-MBO	28	Kenya	Mbololo	Soil	0.01825
Soil-29-MBO	29	Kenya	Mbololo	Soil	0.02582
Soil-29-MBO	29	Kenya	Mbololo	Soil	0.02733
Soil-30-MBO	30	Kenya	Mbololo	Soil	0.01709
Soil-30-MBO	30	Kenya	Mbololo	Soil	0.01895
Soil-31-Baringo	31	Kenya	Baringo	Soil	0.03497
Soil-31-Baringo	31	Kenya	Baringo	Soil	0.03423
Soil-32-Baringo	32	Kenya	Baringo	Soil	0.03460
Soil-32-Baringo	32	Kenya	Baringo	Soil	0.03451
Soil-33-Baringo	33	Kenya	Baringo	Soil	0.01035
Soil-33-Baringo	33	Kenya	Baringo	Soil	0.01210
Soil-34-Baringo	34	Kenya	Baringo	Soil	0.00827
Soil-34-Baringo	34	Kenya	Baringo	Soil	0.00768
Soil-35-Baringo	35	Kenya	Baringo	Soil	0.04143
Soil-35-Baringo	35	Kenya	Baringo	Soil	0.04848
Soil-36-Baringo	36	Kenya	Baringo	Soil	0.01356
Soil-36-Baringo	36	Kenya	Baringo	Soil	0.01329
Soil-37-Ramogi	37	Kenya	Ramogi	Soil	0.01933
Soil-37-Ramogi	37	Kenya	Ramogi	Soil	0.02032
Soil-38-Ramogi	38	Kenya	Ramogi	Soil	0.01792
Soil-38-Ramogi	38	Kenya	Ramogi	Soil	0.01815
Soil-39-Ramogi	39	Kenya	Ramogi	Soil	0.01927
Soil-39-Ramogi	39	Kenya	Ramogi	Soil	0.02185
Soil-40-Ramogi	40	Kenya	Ramogi	Soil	0.01854
Soil-40-Ramogi	40	Kenya	Ramogi	Soil	0.01950
Soil-41-Ramogi	41	Kenya	Ramogi	Soil	0.01535
Soil-41-Ramogi	41	Kenya	Ramogi	Soil	0.01534

Sample_ID	Household_ID	Country	Locality	Category	Se-P
Soil-42-Ramogi	42	Kenya	Ramogi	Soil	0.01890
Soil-42-Ramogi	42	Kenya	Ramogi	Soil	0.02005
Soil-43-Ramogi	43	Kenya	Ramogi	Soil	0.01921
Soil-43-Ramogi	43	Kenya	Ramogi	Soil	0.02001
Soil-44-Ramogi	44	Kenya	Ramogi	Soil	0.00732
Soil-44-Ramogi	44	Kenya	Ramogi	Soil	0.00906
Soil-45-Malindi	45	Kenya	Malindi	Soil	0.01199
Soil-45-Malindi	45	Kenya	Malindi	Soil	0.01200
Soil-46-Malindi	46	Kenya	Malindi	Soil	0.01105
Soil-46-Malindi	46	Kenya	Malindi	Soil	0.01133
Soil-47-Malindi	47	Kenya	Malindi	Soil	0.02049
Soil-47-Malindi	47	Kenya	Malindi	Soil	0.01771
Soil-48-Malindi	48	Kenya	Malindi	Soil	0.01730
Soil-48-Malindi	48	Kenya	Malindi	Soil	0.01868
Soil-49-Malindi	49	Kenya	Malindi	Soil	0.01343
Soil-49-Malindi	49	Kenya	Malindi	Soil	0.01311
Soil-50-Malindi	50	Kenya	Malindi	Soil	0.01327
Soil-50-Malindi	50	Kenya	Malindi	Soil	0.01326
Soil-51-Malindi	51	Kenya	Malindi	Soil	0.02905
Soil-51-Malindi	51	Kenya	Malindi	Soil	0.03001
Soil-52-Malindi	52	Kenya	Malindi	Soil	0.02102
Soil-52-Malindi	52	Kenya	Malindi	Soil	0.02097
Soil-53-Malindi	53	Kenya	Malindi	Soil	0.01295
Soil-53-Malindi	53	Kenya	Malindi	Soil	0.01282
Soil-54-Malindi	54	Kenya	Malindi	Soil	0.02474
Soil-54-Malindi	54	Kenya	Malindi	Soil	0.02475
Soil-55-Malindi	55	Kenya	Malindi	Soil	0.02961
Soil-55-Malindi	55	Kenya	Malindi	Soil	0.02992
Soil-56-Ukunda	56	Kenya	Ukunda	Soil	0.01544
Soil-56-Ukunda	56	Kenya	Ukunda	Soil	0.01407

Sample_ID	Household_ID	Country	Locality	Category	Se-P
Soil-57-Ukunda	57	Kenya	Ukunda	Soil	0.00964
Soil-57-Ukunda	57	Kenya	Ukunda	Soil	0.00931
Soil-58-Ukunda	58	Kenya	Ukunda	Soil	0.01265
Soil-58-Ukunda	58	Kenya	Ukunda	Soil	0.01246
Soil-59-Ukunda	59	Kenya	Ukunda	Soil	0.01597
Soil-59-Ukunda	59	Kenya	Ukunda	Soil	0.01776
Soil-60-Ukunda	60	Kenya	Ukunda	Soil	0.01587
Soil-60-Ukunda	60	Kenya	Ukunda	Soil	0.01635
Soil-61-Ukunda	61	Kenya	Ukunda	Soil	0.01238
Soil-61-Ukunda	61	Kenya	Ukunda	Soil	0.01269
Soil-62-Ukunda	62	Kenya	Ukunda	Soil	0.01342
Soil-62-Ukunda	62	Kenya	Ukunda	Soil	0.01250
ETS0001	ETH001	Ethiopia	Derashe	Soil	0.00370
ETS0001	ETH001	Ethiopia	Derashe	Soil	0.00309
ETS0002	ETH002	Ethiopia	Derashe	Soil	0.00991
ETS0002	ETH002	Ethiopia	Derashe	Soil	0.00893
ETS0003	ETH003	Ethiopia	Derashe	Soil	0.00605
ETS0003	ETH003	Ethiopia	Derashe	Soil	0.00595
ETS0004	ETH004	Ethiopia	Derashe	Soil	0.00164
ETS0004	ETH004	Ethiopia	Derashe	Soil	0.00147
ETS0005	ETH005	Ethiopia	Derashe	Soil	0.00130
ETS0005	ETH005	Ethiopia	Derashe	Soil	0.00115
ETS0006	ETH006	Ethiopia	Derashe	Soil	0.00597
ETS0006	ETH006	Ethiopia	Derashe	Soil	0.00590
ETS0007	ETH007	Ethiopia	Derashe	Soil	0.00735
ETS0007	ETH007	Ethiopia	Derashe	Soil	0.00734
ETS0008	ETH008	Ethiopia	Derashe	Soil	0.00289
ETS0008	ETH008	Ethiopia	Derashe	Soil	0.00279
ETS0009	ETH009	Ethiopia	Derashe	Soil	0.00530
ETS0009	ETH009	Ethiopia	Derashe	Soil	0.00545

Sample_ID	Household_ID	Country	Locality	Category	Se-P
ETS0010	ETH010	Ethiopia	Derashe	Soil	0.01321
ETS0010	ETH010	Ethiopia	Derashe	Soil	0.01416
ETS0011	ETH011	Ethiopia	Derashe	Soil	0.00425
ETS0011	ETH011	Ethiopia	Derashe	Soil	0.00466
ETS0012	ETH012	Ethiopia	Derashe	Soil	0.00841
ETS0012	ETH012	Ethiopia	Derashe	Soil	0.00773
ETS0013	ETH013	Ethiopia	Konso	Soil	0.00189
ETS0013	ETH013	Ethiopia	Konso	Soil	0.00186
ETS0014	ETH014	Ethiopia	Konso	Soil	0.00183
ETS0014	ETH014	Ethiopia	Konso	Soil	0.00178
ETS0015	ETH015	Ethiopia	Konso	Soil	0.00289
ETS0015	ETH015	Ethiopia	Konso	Soil	0.00240
ETS0016	ETH016	Ethiopia	Konso	Soil	0.00183
ETS0016	ETH016	Ethiopia	Konso	Soil	0.00196
ETS0017	ETH017	Ethiopia	Konso	Soil	0.00500
ETS0017	ETH017	Ethiopia	Konso	Soil	0.00572
ETS0018	ETH018	Ethiopia	Konso	Soil	0.00658
ETS0018	ETH018	Ethiopia	Konso	Soil	0.00694
ETS0019	ETH019	Ethiopia	Konso	Soil	0.00701
ETS0019	ETH019	Ethiopia	Konso	Soil	0.00707
ETS0020	ETH020	Ethiopia	Konso	Soil	0.00740
ETS0020	ETH020	Ethiopia	Konso	Soil	0.00666
ETS0021	ETH021	Ethiopia	Konso	Soil	0.00782
ETS0021	ETH021	Ethiopia	Konso	Soil	0.00708
ETS0022	ETH022	Ethiopia	Konso	Soil	0.00187
ETS0022	ETH022	Ethiopia	Konso	Soil	0.00165
ETS0023	ETH023	Ethiopia	Konso	Soil	0.00520
ETS0023	ETH023	Ethiopia	Konso	Soil	0.00497
ETS0024	ETH024	Ethiopia	Konso	Soil	0.00508
ETS0024	ETH024	Ethiopia	Konso	Soil	0.00502

Sample_ID	Household_ID	Country	Locality	Category	Se-P
ETS0025	Eth-Haw-1	Ethiopia	Hawasa	Soil	0.01893
ETS0025	Eth-Haw-1	Ethiopia	Hawasa	Soil	0.01899
ETS0026	Eth-Haw-2	Ethiopia	Hawasa	Soil	0.00817
ETS0026	Eth-Haw-2	Ethiopia	Hawasa	Soil	0.00811
ETS0027	Eth-Haw-3	Ethiopia	Hawasa	Soil	0.00678
ETS0027	Eth-Haw-3	Ethiopia	Hawasa	Soil	0.00730
ETS0028	Eth-Haw-4	Ethiopia	Hawasa	Soil	0.00679
ETS0028	Eth-Haw-4	Ethiopia	Hawasa	Soil	0.00741
ETS0029	Eth-Haw-5	Ethiopia	Hawasa	Soil	0.01324
ETS0029	Eth-Haw-5	Ethiopia	Hawasa	Soil	0.01292
ETS0030	Eth-Haw-6	Ethiopia	Hawasa	Soil	0.00550
ETS0030	Eth-Haw-6	Ethiopia	Hawasa	Soil	0.00483
ETS0031	Eth-Haw-7	Ethiopia	Hawasa	Soil	0.01664
ETS0031	Eth-Haw-7	Ethiopia	Hawasa	Soil	0.01751
ETS0032	Eth-Haw-8	Ethiopia	Hawasa	Soil	0.01541
ETS0032	Eth-Haw-8	Ethiopia	Hawasa	Soil	0.01503
ETS0033	Eth-Haw-9	Ethiopia	Hawasa	Soil	0.00995
ETS0033	Eth-Haw-9	Ethiopia	Hawasa	Soil	0.00962
2711A	999	Reference	Reference	Soil	0.13309
2711A	999	Reference	Reference	Soil	0.13636
2711A	999	Reference	Reference	Soil	0.13831
