# Supplementary material for: Variation in the mineral element concentration of Moringa oleifera Lam. and M. stenopetala (Bak. f.) Cuf.: Role in human nutrition
Source: PLoS One. 2017 Apr 7;12(4):e0175503. doi: 10.1371/journal.pone.0175503 (PMC5384779; doi:10.1371/journal.pone.0175503)
Supplement: S40 Table — The figures below the yellow diagonal are correlation coefficients and those above the diagonal are p values. ** Correlation is significant at the 0.01 level (2-tailed). * Correlation is significant at the 0.05 level (2-tailed). N = 18 (PDF) [file pone.0175503.s040.pdf]

S40 Table. Correlation between elemental concentrations of MO edible parts (flower, immature pod, leaf and seed). ** Correlation is significant at the 0.01 level (2-tailed). * Correlation is significant at the 0.05 level (2-tailed). N = 18.

	Ca_Flower	Cu_Flower	Fe_Flower	Mg_Flower	Se_Flower	Zn_Flower	Ca_Seed	Cu_Seed	Fe_Seed	Mg_Seed	Se_Seed	Zn_Seed	Ca_Leaf	Cu_Leaf	Fe_Leaf	Mg_Leaf	Se_Leaf	Zn_Leaf	Ca_Immature pod	Cu_Immature pod	Fe_Immature pod	Mg_Immature pod	Se_Immature pod	Zn_Immature pod
Ca_Flower																								
Cu_Flower	0.096																							
Fe_Flower	0.259	0.608**																						
Mg_Flower	0.818**	0.261	0.36																					
Se_Flower	0.424	-0.284	-0.238	0.42																				
Zn_Flower	0.079	0.647**	0.284	0.284	-0.063																			
Ca_Seed	-0.164	-0.525*	-0.315	-0.015	0.098	-0.447																		
Cu_Seed	0.317	0.278	0.22	0.585*	0.189	0.127	-0.342																	
Fe_Seed	-0.141	0.732**	0.364	0.007	-0.323	0.391	-0.42	0.292																
Mg_Seed	0.069	0.401	-0.022	0.104	-0.063	0.480*	-0.389	0.102	0.075															
Se_Seed	0.465	-0.292	-0.203	0.560*	0.785**	-0.032	0.259	0.22	-0.397	-0.053														
Zn_Seed	0.018	0.785**	0.482*	0.331	-0.065	0.385	-0.154	0.346	0.719**	0.075	-0.055													
Ca_Leaf	0.35	-0.484*	-0.571*	0.288	0.226	-0.249	0.459	0.034	-0.428	0.015	0.434	-0.416												
Cu_Leaf	0.102	0.713**	0.505*	0.16	-0.309	0.251	-0.288	0.135	0.393	0.214	-0.317	0.467	-0.42											
Fe_Leaf	-0.03	-0.073	0.164	-0.315	-0.428	-0.102	0.129	-0.697**	-0.354	0.23	-0.278	-0.34	-0.005	0.11										
Mg_Leaf	0.408	-0.304	-0.15	0.562*	0.375	-0.084	0.515*	0.094	-0.482*	-0.042	0.579*	-0.079	0.583*	-0.16	0.02									
Se_Leaf	0.408	-0.296	-0.17	0.422	0.926**	-0.036	0.034	0.337	-0.187	-0.232	0.730**	-0.069	0.201	-0.412	-0.579*	0.29								
Zn_Leaf	0.028	0.472*	0.583*	0.212	0.096	0.608**	-0.234	0.003	0.344	-0.168	0.129	0.406	-0.484*	0.321	-0.119	-0.096	0.176							
Ca_Immature pod	0.746**	0.131	0.143	0.542*	0.348	-0.032	-0.247	0.255	0.106	0.135	0.278	0.015	0.356	-0.038	-0.046	0.28	0.366	-0.119						
Cu_Immature pod	-0.051	0.639**	0.412	-0.061	-0.307	0.129	-0.106	-0.189	0.42	0.187	-0.408	0.428	-0.416	0.777**	0.278	-0.253	-0.424	0.187	0.104					
Fe_Immature pod	-0.003	0.255	0.43	-0.137	-0.224	-0.09	0.053	-0.404	0.226	0.156	-0.414	0.104	-0.315	0.379	0.531*	-0.108	-0.333	0.053	0.234	.628**				
Mg_Immature pod	0.593**	0.179	0.148	0.523*	0.104	-0.139	-0.034	0.236	0.156	0.162	0.131	0.115	0.37	0.205	0.02	0.422	0.053	-0.232	0.856**	0.3	0.401			
Se_Immature pod	0.527*	-0.247	-0.195	0.523*	0.938**	-0.036	0.112	0.267	-0.191	-0.137	0.769**	0.015	0.243	-0.358	-0.498*	0.366	0.930**	0.059	0.434	-0.342	-0.22	0.185		
Zn_Immature pod	-0.24	0.507*	0.187	-0.251	-0.527*	0.218	-0.16	-0.218	0.558*	0.335	-0.678**	0.253	-0.354	0.519*	0.228	-0.414	-0.583*	-0.051	0.02	.767**	.655**	0.255	-0.463	
