# Supplementary material for: Variation in the mineral element concentration of Moringa oleifera Lam. and M. stenopetala (Bak. f.) Cuf.: Role in human nutrition
Source: PLoS One. 2017 Apr 7;12(4):e0175503. doi: 10.1371/journal.pone.0175503 (PMC5384779; doi:10.1371/journal.pone.0175503)
Supplement: S41 Table — * Correlation is significant at the 0.05 level (2-tailed). N = 6. (PDF) [file pone.0175503.s041.pdf]

S41 Table. Correlation between the elemental composition of MO and amaranth leaves. * Correlation is significant at the 0.05 level (2-tailed). N = 6

	Ca_MO	Cu_MO	Fe_MO	Mg_MO	Se_MO	Zn_MO	Ca_AM	Cu_AM	Fe-AM	Mg_AM	Se_AM	Zn_AM
Ca_MO												
Cu_MO	-0.257											
Fe_MO	-0.314	0.6										
Mg_MO	0.029	-0.086	0.543									
Se_MO	0.486	0.143	-0.371	-0.2								
Zn_MO	0.086	-0.029	-0.086	-0.657	0.029							
Ca_AM	-0.657	-0.086	-0.143	-0.371	-0.714	0.029						
Cu_AM	0.086	-0.029	0.371	-0.086	-0.657	0.543	0.257					
Fe-AM	-0.657	0.429	0.829*	0.6	-0.6	-0.429	0.257	0.143				
Mg_AM	-0.371	0.771	0.371	0.143	0.029	-0.6	0.143	-0.371	0.543			
Se_AM	-0.086	-0.771	-0.829*	-0.371	0.086	0.143	0.314	-0.314	-0.543	-0.543		
Zn_AM	0.029	-0.029	0.714	0.543	-0.371	0.257	-0.314	0.6	0.429	-0.371	-0.429	
