# Supplementary material for: Variation in the mineral element concentration of Moringa oleifera Lam. and M. stenopetala (Bak. f.) Cuf.: Role in human nutrition
Source: PLoS One. 2017 Apr 7;12(4):e0175503. doi: 10.1371/journal.pone.0175503 (PMC5384779; doi:10.1371/journal.pone.0175503)
Supplement: S42 Table — ** Correlation is significant at the 0.05 level (2-tailed). N = 4. (PDF) [file pone.0175503.s042.pdf]

S42 Table. Correlation between the elemental composition of MO and brassica (BO) leaves. ** Correlation is significant at the 0.05 level (2-tailed). N = 3.

	Ca_MO	Cu_MO	Fe_MO	Mg_MO	Se_MO	Zn_MO	Ca_BO	Cu_BO	Fe_BO	MG_BO	Se_BO	Zn_BO
Ca_MO												
Cu_MO	-0.4											
Fe_MO	0.6	-0.4										
Mg_MO	0.8	-0.8	0.8									
Se_MO	0.2	0.8	-0.2	-0.4								
Zn_MO	0.2	0.8	-0.2	-0.4	1.000**							
Ca_BO	-1.000**	0.4	-0.6	-0.8	-0.2	-0.2						
Cu_BO	-1.000**	0.4	-0.6	-0.8	-0.2	-0.2	1.000**					
Fe_BO	-0.8	0	-0.8	-0.6	-0.4	-0.4	0.8	0.8				
MG_BO	-1.000**	0.4	-0.6	-0.8	-0.2	-0.2	1.000**	1.000**	0.8			
Se_BO	0.4	0.6	0.4	0	0.8	0.8	-0.4	-0.4	-0.8	-0.4		
Zn_BO	1.000**	-0.4	0.6	0.8	0.2	0.2	-1.000**	-1.000**	-0.8	-1.000**	0.4	
