# Supplementary material for: Variation in the mineral element concentration of Moringa oleifera Lam. and M. stenopetala (Bak. f.) Cuf.: Role in human nutrition
Source: PLoS One. 2017 Apr 7;12(4):e0175503. doi: 10.1371/journal.pone.0175503 (PMC5384779; doi:10.1371/journal.pone.0175503)
Supplement: S43 Table — ** Correlation is significant at the 0.05 level (2-tailed). N = 3. (PDF) [file pone.0175503.s043.pdf]

S43 Table. Correlation between the elemental composition of amaranth (AM) and brassica (BO) leaves. ** Correlation is significant at the 0.05 level (2-tailed). N = 3.

	Ca_AM	Cu_AM	Fe-AM	Mg_AM	Se_AM	Zn_AM	Ca_BO	Cu_BO	Fe_BO	MG_BO	Se_BO	Zn_BO
Ca_AM	1											
Cu_AM	-0.5	1										
Fe-AM	0.5	0.5	1									
Mg_AM	0.5	-1.000**	-0.5	1								
Se_AM	1.000**	-0.5	0.5	0.5	1							
Zn_AM	-0.5	1.000**	0.5	-1.000**	-0.5	1						
Ca_BO	1.000**	-0.5	0.5	0.5	1.000**	-0.5	1					
Cu_BO	1.000**	-0.5	0.5	0.5	1.000**	-0.5	1.000**	1				
Fe_BO	1.000**	-0.5	0.5	0.5	1.000**	-0.5	1.000**	1.000**	1			
MG_BO	1.000**	-0.5	0.5	0.5	1.000**	-0.5	1.000**	1.000**	1.000**	1		
Se_BO	-1.000**	0.5	-0.5	-0.5	-1.000**	0.5	-1.000**	-1.000**	-1.000**	-1.000**	1	
Zn_BO	-1.000**	0.5	-0.5	-0.5	-1.000**	0.5	-1.000**	-1.000**	-1.000**	-1.000**	1.000**	1
